# Long-read assembly of the *Brassica napus* reference genome Darmor-bzh

**DOI:** 10.1093/gigascience/giaa137

**Published:** 2020-12-15

**Authors:** Mathieu Rousseau-Gueutin, Caroline Belser, Corinne Da Silva, Gautier Richard, Benjamin Istace, Corinne Cruaud, Cyril Falentin, Franz Boideau, Julien Boutte, Regine Delourme, Gwenaëlle Deniot, Stefan Engelen, Julie Ferreira de Carvalho, Arnaud Lemainque, Loeiz Maillet, Jérôme Morice, Patrick Wincker, France Denoeud, Anne-Marie Chèvre, Jean-Marc Aury

**Affiliations:** IGEPP, INRAE, Institut Agro, Université de Rennes, Domaine de la Motte, 35653 Le Rheu, France; Génomique Métabolique, Genoscope, Institut François Jacob, CEA, CNRS, Univ Evry, Université Paris-Saclay, 2 rue Gaston Crémieux, 91057 Evry, France; Génomique Métabolique, Genoscope, Institut François Jacob, CEA, CNRS, Univ Evry, Université Paris-Saclay, 2 rue Gaston Crémieux, 91057 Evry, France; IGEPP, INRAE, Institut Agro, Université de Rennes, Domaine de la Motte, 35653 Le Rheu, France; Génomique Métabolique, Genoscope, Institut François Jacob, CEA, CNRS, Univ Evry, Université Paris-Saclay, 2 rue Gaston Crémieux, 91057 Evry, France; Genoscope, Institut François Jacob, Commissariat à l'Energie Atomique (CEA), Université Paris-Saclay, 2 rue Gaston Crémieux, 91057 Evry, France; IGEPP, INRAE, Institut Agro, Université de Rennes, Domaine de la Motte, 35653 Le Rheu, France; IGEPP, INRAE, Institut Agro, Université de Rennes, Domaine de la Motte, 35653 Le Rheu, France; IGEPP, INRAE, Institut Agro, Université de Rennes, Domaine de la Motte, 35653 Le Rheu, France; IGEPP, INRAE, Institut Agro, Université de Rennes, Domaine de la Motte, 35653 Le Rheu, France; IGEPP, INRAE, Institut Agro, Université de Rennes, Domaine de la Motte, 35653 Le Rheu, France; Génomique Métabolique, Genoscope, Institut François Jacob, CEA, CNRS, Univ Evry, Université Paris-Saclay, 2 rue Gaston Crémieux, 91057 Evry, France; IGEPP, INRAE, Institut Agro, Université de Rennes, Domaine de la Motte, 35653 Le Rheu, France; Genoscope, Institut François Jacob, Commissariat à l'Energie Atomique (CEA), Université Paris-Saclay, 2 rue Gaston Crémieux, 91057 Evry, France; IGEPP, INRAE, Institut Agro, Université de Rennes, Domaine de la Motte, 35653 Le Rheu, France; IGEPP, INRAE, Institut Agro, Université de Rennes, Domaine de la Motte, 35653 Le Rheu, France; Génomique Métabolique, Genoscope, Institut François Jacob, CEA, CNRS, Univ Evry, Université Paris-Saclay, 2 rue Gaston Crémieux, 91057 Evry, France; Génomique Métabolique, Genoscope, Institut François Jacob, CEA, CNRS, Univ Evry, Université Paris-Saclay, 2 rue Gaston Crémieux, 91057 Evry, France; IGEPP, INRAE, Institut Agro, Université de Rennes, Domaine de la Motte, 35653 Le Rheu, France; Génomique Métabolique, Genoscope, Institut François Jacob, CEA, CNRS, Univ Evry, Université Paris-Saclay, 2 rue Gaston Crémieux, 91057 Evry, France

**Keywords:** oilseed rape, *Brassica*, Darmor-bzh, nanopore sequencing, optical mapping, assembly, chromosome-scale, direct RNA

## Abstract

**Background:**

The combination of long reads and long-range information to produce genome assemblies is now accepted as a common standard. This strategy not only allows access to the gene catalogue of a given species but also reveals the architecture and organization of chromosomes, including complex regions such as telomeres and centromeres. The *Brassica* genus is not exempt, and many assemblies based on long reads are now available. The reference genome for *Brassica napus*, Darmor-bzh, which was published in 2014, was produced using short reads and its contiguity was extremely low compared with current assemblies of the *Brassica* genus.

**Findings:**

Herein, we report the new long-read assembly of Darmor-bzh genome (*Brassica napus*) generated by combining long-read sequencing data and optical and genetic maps. Using the PromethION device and 6 flowcells, we generated ∼16 million long reads representing 93× coverage and, more importantly, 6× with reads longer than 100 kb. This ultralong-read dataset allows us to generate one of the most contiguous and complete assemblies of a *Brassica* genome to date (contig N50 > 10 Mb). In addition, we exploited all the advantages of the nanopore technology to detect modified bases and sequence transcriptomic data using direct RNA to annotate the genome and focus on resistance genes.

**Conclusion:**

Using these cutting-edge technologies, and in particular by relying on all the advantages of the nanopore technology, we provide the most contiguous *Brassica napus* assembly, a resource that will be valuable to the *Brassica* community for crop improvement and will facilitate the rapid selection of agronomically important traits.

## Background

Recently, Pacific Biosciences (PacBio) and Oxford Nanopore Technologies (ONT) sequencing technologies were commercialized with the promise of sequencing long DNA fragments (on the order of kilobases to megabases). The introduction of these long-read technologies significantly increased the quality in terms of contiguity and completeness of genome assemblies [1–3]. Although it is possible to assemble chromosomes into a single contig in the case of simple genomes [4], the complexity of plant genomes is such that these methods are still insufficient to obtain the chromosome architecture. The resulting contigs need to be ordered and oriented with long-range information based on chromatin interactions or optical maps. This combined strategy is now a common standard.

*Brassica* crops include important vegetables for human nutrition and for production of vegetable oil. It comprises several well-studied species that belong to the triangle of U: 3 diploid species, *Brassica rapa* (AA), *Brassica nigra* (BB), and *Brassica oleracea* (CC); and 3 allotetraploid hybrid species, *Brassica juncea* (AABB), *Brassica napus* (AACC), and *Brassica carinata* (BBCC). As *Brassica* species underwent several polyploidy events, the genomes of the diploid and allopolyploid species are important models to shed light on the immediate and long-term effect of polyploidy on the structural and the functional evolutionary dynamic of duplicated genes and genomes. These events had a significant role in their diversification; indeed the variability between 2 morphotypes of the same *Brassica* species can be high, underlining the importance of having a collection of high-quality assemblies for the *Brassica* genus and more generally for all species.

To date, several genomes of the *Brassica* genus have been sequenced using long reads and brought to the chromosome scale. The first 2, *B. rapa* Z1 and *B. oleracea* HDEM, were published in 2018 and generated using ONT reads and optical maps [2, 4]. At the beginning of 2020, 2 genomes of *B. rapa* (Chiifu) and *B. oleracea* (D134) were released and sequenced using PacBio long reads [5, 6]. A first long-read assembly based on ONT of the other diploid species, *B. nigra* NI100, has been available since the beginning of 2020 [7]. The earlier availability of the diploid genomes may be explained by their smaller genome size (<600 Mb) compared with their tetraploid derivatives. The first genome of a tetraploid plant was published in 2014 [8] and was sequenced using a short-read strategy (referred here as *B. napus* Darmor-bzh v5), and although a great resource for the scientific community, this genome remains very fragmented and incomplete. In 2017, the pseudomolecules of Darmor-bzh were improved [9] using genotyping by sequencing data (referred to here as Darmor-bzh v8), and a new gene prediction was made. Unfortunately, this annotation was not used by the *Brassica* community because it was considered incomplete [10]. As an illustration, only 89.6% of the core brassicale genes were found in the annotation compared to the 97.7% of the previous version (Table 1). In the first months of 2020, 9 *B. napus* genomes based on PacBio sequencing were published [11, 12], and 3 of them were organized using long-range information. These genomes have a better continuity than the current reference (Darmor-bzh), but interestingly their N50 at the contig level is lower than that of the *Brassica* genomes that have been generated using ONT sequencing (Tables 2 and 3). Here, we report the genome sequence of the *B. napus* (NCBI:txid3708) reference Darmor-bzh (referred here as Darmor-bzh v10), produced using ONT and Illumina reads supplemented by optical and genetic maps. In addition to chromosome architecture, genes were predicted by sequencing native RNA molecules using the ONT PromethION with a focus on resistance genes. The contiguity and gene completion of this new genome assembly are among the highest in the *Brassica* genus (Table 3 and Supplementary Table S9).

**Table 1.** Statistics of the Darmor-bzh *B. napus* assemblies

**Version**	Technology	Contig N50 (L50)	No. of contigs	Cumulative size (Mb)	No. of genes	Number of anchored bases in Mb (% of the assembly)	Number of anchored [ACGT] bases in Mb	BUSCO scores
10	ONT	11,486,274 (24)	505	924	108,190	867 (93.84%)	849	C: 98.6% S: 7.0% D: 91.6% F: 0.1% M: 1.3%
8	454	37,644 (5,517)	44,818	850	80,382	799 (93.96%)	690	C: 89.6% S: 18.0% D: 71.6% F: 3.4% M: 7.0%
5	454	37,644 (5,517)	44,837	850	101,040	645 (75.90%)	553	C: 97.7% S: 9.9% D: 87.8% F: 0.5% M: 1.8%

BUSCO scores are calculated for n = 4,596. C: complete; S: complete and single-copy; D: complete and duplicated; F: fragmented; M: missing.

**Table 2.** General information about the available *Brassica* long reads and older Darmor-bzh assemblies

Species and subgenome	Genotype	Principal sequencing technology	Release year	Reference
*B. rapa* A	Z1	ONT	2018	Belser et al. [2]
	Chiifu	PacBio	2018	Zhang et al. [58]
*B. nigra* B	NI100	ONT	2020	Perumal et al. [7]
*B. oleracea* C	HDEM	ONT	2018	Belser et al. [2]
	D134	PacBio	2020	Honghao et al. [5]
*B. napus* AC	Darmor-bzh v5	454	2014	Chalhoub et al. [8]
	Darmor-bzh v8	454	2017	Bayer et al. [9, 12]
	Darmor-bzh v10	ONT	2020	This study
	Westar	PacBio	2020	Song et al. [11]
	Express617	PacBio	2020	Lee et al. [12]
	Tapidor3	PacBio	2020	Song et al. [11]
	Shengli3	PacBio	2020	Song et al. [11]
	QuintaA	PacBio	2020	Song et al. [11]
	No2127	PacBio	2020	Song et al. [11]
	GanganF73	PacBio	2020	Song et al. [11]
	Zheyou73	PacBio	2020	Song et al. [11]
	Zs11	PacBio	2020	Song et al. [11]

**Table 3.** Statistics of the *Brassica* long-read assemblies, ordered by contig N50 value

Species	Genotype	Technology	Contig N50 (L50)	No. contigs	Maximum contig size	No. scaffolds	Cumulative size (Mb)	No. of genes	Complete BUSCO (%)
*B. nigra*	NI100	ONT	11,504,526 (13)	187	32,581,047	58	506	59,851	97.2
*B. napus*	Darmor-bzh	ONT	11,486,274 (24)	505	46,624,681	237	924	108,190	98.6
*B. oleracea*	HDEM	ONT	9,491,203 (19)	264	26,712,175	129	555	61,279	96.2
*B. rapa*	Z1	ONT	5,519,976 (17)	627	22,127,468	304	402	46,721	96.7
*B. oleracea*	D134	PacBio	3,591,417 (43)	229	15,061,548	9	530	43,868	95.1
*B. napus*	Westar	PacBio	3,130,520 (93)	5,602	15,044,602	3,458	1,008	100,194	98.3
*B. napus*	Express617	PacBio	3,002,211 (78)	2,092	17,406,538	1,431	925	99,481	98.8
*B. napus*	Tapidor3	PacBio	2,855,025 (104)	5,318	11,486,821	3,566	1,014	105,409	98.4
*B.napus*	Shengli3	PacBio	2,825,656 (104)	6,024	14,590,814	3,802	1,002	103,920	98.1
*B. napus*	QuintaA	PacBio	2,801,289 (104)	5,835	14,779,276	3,722	1,004	98,755	98.4
*B. napus*	No2127	PacBio	2,704,645 (98)	5,787	12,981,515	3,733	**1,012**	105,894	98.0
*B. napus*	GanganF73	PacBio	2,696,026 (98)	6,887	18,404,683	4,930	**1,032**	106,567	98.4
*B. napus*	Zheyou73	PacBio	2,103,870 (126)	7,399	11,351,519	4,990	1,016	105,842	97.9
*B. napus*	Zs11	PacBio	1,506,624 (186)	8,773	10,640,036	3,332	**1,010**	107,233	98.5
*B. rapa*	Chiifu	PacBio	1,386,548 (71)	1,507	9,417,752	1,099	353	60,609	99.6

BUSCO completeness is calculated for n = 4,596. Full BUSCO scores are available in Supplementary Table S9.

### DNA extraction and sequencing

#### Plant material

*B. napus* cv. Darmor-bzh is a French winter variety, freely available in Plant Genetic Resources BrACySol (Rennes, France). Darmor-bzh seeds were sown in Fertiss blocks (Fertil, France). Plantlets were grown under 16 h light/8 h night in a greenhouse at 20°C for 10–12 days of culture. Prior to harvest, plants were either dark treated during 5 days for DNA extraction or not treated after the 10–12 days of culture for RNA extraction.

#### DNA and RNA extractions

*B. napus* Darmor-bzh DNA extractions for nanopore sequencing were prepared from 1 cm^2^ of first young leaves from which the midribs were removed. Samples were placed on aluminium foil on ice. A 2.5-g quantity of each genotype was ground with mortar and pestle in liquid nitrogen for 1 minute. Ground materials were homogenized with 10 mL pre-heated CF lysis buffer (MACHEREY-NAGEL GmbH & Co. KG, Germany) supplemented with 4 mg proteinase K in 50-mL tubes containing phase-lock gel and incubated for 45 min at 56°C. A 10-mL quantity of saturated phenol (25:24:1) was added to samples, and tubes were placed on a rotator at 40 rpm for 10 min to get a fine emulsion. Samples were centrifuged for 24 min at 4,500 *g* (Acc3/Dec3), and aqueous phases were poured in a new 50-mL tube containing phase-lock gel. Then 10 mL of chloroform-octanol (24:1) were added to each sample and tubes were placed on a rotator at 40 rpm for 10 min to get a fine emulsion. Samples were centrifuged for 24 min at 4,500*g* (Acc3/Dec3) and aqueous phases were poured in a new 50-mL tube. DNA was precipitated out by adding 4 mL of NaCl 5 M and 30 mL of cold 100% isopropanol. After 3 h at 4°C, DNA was hooked out in 1 piece with a hook by melting a glass capillary in a blue flame. DNA pellets were submerged in a 50-mL tube containing 70% ethanol and transferred in a new tube to evaporate the remaining isopropanol at 37°C (oven). Dried DNA was resuspended with a 3-mL TE 10/1 buffer. Quality of extraction was evaluated using a 2200 TapeStation System (Agilent Technologies, Santa Clara, CA, USA) with a Genomic DNA kit.

Total RNA was extracted from 2.5 g *B. napus* leaf and root tissues using LiCl modified protocol [13]. RNA was controlled using a Bioanalyzer Chip (Agilent RNA 6000 Pico Kit) and quantified using Qubit (RNA HS Assay Kit).

#### Illumina sequencing

DNA (1.5 μg) was sonicated to a 100–1,500-bp size range using a Covaris E220 sonicator (Covaris, Woburn, MA, USA). Fragments (1 µg) were end-repaired and 3′-adenylated and Illumina adapters were then added using the Kapa Hyper Prep Kit (KapaBiosystems, Wilmington, MA, USA). Ligation products were purified with AMPure XP beads (Beckman Coulter Genomics, Danvers, MA, USA). Libraries were then quantified by qPCR using the KAPA Library Quantification Kit for Illumina Libraries (KapaBiosystems, Wilmington, MA, USA), and library profiles were assessed using a DNA High Sensitivity LabChip kit on an Agilent Bioanalyzer (Agilent Technologies, Santa Clara, CA, USA). Libraries were sequenced on an Illumina HiSeq2500 instrument (Illumina, San Diego, CA, USA) using 250 base-length read chemistry in a paired-end mode.

After the Illumina sequencing, an in-house quality control process was applied to the reads that passed the Illumina quality filters as described by Alberti et al. [14]. These trimming and removal steps were achieved using Fastxtend tools [15]. This processing resulted in high-quality data and improvement of the subsequent analyses (Supplementary Table S1).

#### Nanopore sequencing (PromethION)

The libraries were prepared according to the protocol Genomic DNA by ligation (SQK-LSK108 and SQK-LSK109 Kit)—PromethION provided by ONT. Genomic DNA was first repaired and end-prepped with the NEBNext FFPE Repair Mix (New England Biolabs [NEB], Ipswich, MA, USA) and the NEBNext® Ultra™ II End Repair/dA-Tailing Module (NEB). DNA was then purified with AMPure XP beads (Beckman Coulter, Brea, CA, USA) and sequencing adapters provided by ONT were ligated using Concentrated T4 DNA Ligase 2,000,000 U/mL (NEB). After purification with AMPure XP beads (Beckman Coulter) using Dilution Buffer (ONT) and Wash Buffer (ONT), the library was mixed with the Sequencing Buffer (ONT) and the Library Loading Bead (ONT) and loaded on the PromethION Flow Cells (R9.4.1). Output reads were base-called using the Guppy base caller (version 1.4.3) with the “fast” configuration and default parameters.

No data cleaning was performed; the nanopore long reads were used raw for all the assemblies (Supplementary Table S1). A taxonomical assignation was performed using Centrifuge (Centrifuge Classifier, RRID:SCR_016665) version 1.0.3 (nr database from August 2018, and the following parameters: –mm -p 5 -x nt_Aug2018 -q -k 15 –min-hitlen 26 –host-taxids 3708 –exclude-taxids 32644,28384,12908,77133) [16] for each dataset to detect potential contaminations. On each run, ∼65% of the reads were unassigned and almost 35%were assigned to the *Brassica* genus. In addition, we detected a small proportion (<0.6%) of reads corresponding to various metazoa.

#### Direct RNA sequencing (PromethION)

Ribosomal RNA was depleted using the Ribo-Zero rRNA Removal Kit (Plant Leaf). RNA libraries were created from a mix of 960  ng messenger RNA according to the ONT protocol “Direct RNA sequencing” (SQK-RNA002). We performed the optional reverse transcription (RT) step to improve throughput, but the complementary DNA strand was not sequenced. The nanopore library was sequenced using the PromethION on a single R9.4.1 flowcell and base-called using the Guppy base caller (version 3.2.8) with the “hac” configuration and default parameters (Supplementary Table S11).

#### Optical maps

*B. napus* Darmor-bzh DNA extractions for nanopore sequencing were prepared from 1 cm^2^ of first young leaves from which the midribs were removed. Samples were placed on aluminium foil on ice. A quantity of 5 g of each genotype was ground with mortar and pestle in liquid nitrogen for 2 minutes. Ground materials were homogenized in 50 mL NIBTM (10 mM Tris-HCl pH 8.0, 10 mM EDTA pH 8.0, 80 mM KCl, 0.5 M sucrose, 1 mM spermine tetrahydrochloride, 1 mM spermidine trihydrochloride, 2% [w/v] PVP40; pH was adjusted to 9.4 and solution was filtered through 0.22 µm [NIB] and supplemented with 0.5% TritonX-100 [NIBT] and 7.5% 2-mercaptoethanol [NIBTM]). Nuclei suspensions were filtered through cheesecloth and Miracloth and centrifuged at 1,500*g* for 20 min at 4°C. Pellets were suspended in 1 mL NIBTM and adjusted to 20 mL with NIBTM. Nuclei suspensions were filtered again through cheesecloth and Miracloth and centrifuged at 57*g* for 2 min at 4°C. Supernatants were kept and centrifuged at 1,500*g* for 20 min at 4°C. Pellets were suspended in 1 mL NIBT and adjusted to 20 mL with NIBT. To wash the pellets, the last steps were repeated 3 times with 50 mL of NIBT and a final time with 50 mL of NIB. Pellets were suspended in residual NIB (∼200 µL), transferred in a 1.5-mL tube, and centrifuged at 1,500*g* for 2 min at 4°C. Nuclei were suspended in cell suspension buffer from CHEF Genomic DNA Plug Kits (Bio-Rad) and melted agarose 2% from the same kit was added to reach a 0.75% agarose plug concentration. Plug lysis and DNA retrieval were performed as recommended by Bionano Genomics.

The NLRS labeling (BspQI) protocol was performed according to Bionano with 600 ng of DNA. The DLS labeling (DLE-1) was performed with 750 ng of DNA. Loading of the chip was performed as recommended by Bionano Genomics.

#### *B. napus* Darmor-bzh genetic map

An integrated *B. napus* genetic map was obtained using the BioMercator V4.2 program [17] from 3 different genetic maps [18]: a cross between Darmor-bzh and Yudal (doubled haploid population with 248 lines), a cross between Darmor and Bristol (F2 population with 291 plants), and a cross between Darmor and Samouraï (doubled haploid population with 129 lines). These populations were genotyped using the Illumina 8K, 20K, and 60K arrays [8,18–20] and genetic maps were constructed using CarthaGene software [21].

### Genome assembly

#### Genome size estimation

The *B. napus* genome size was estimated using flow cytometry in a publication from Johnston et al. [22] that reported a haploid genome size of 1,132 Mb. In addition, we launched Genomescope (GenomeScope, RRID:SCR_017014) version 1 using Illumina reads and a *k-*mer value of 31 and obtained an estimated genome size of 862 Mb (Fig. S1) and a very low heterozygosity rate (0.0526%). The results are highly discordant; however, the genotype used for the flow cytometry experiment is not known. In addition, we observed a high divergence in terms of assembly size between the 10 *B. napus* assemblies (Table 3) and a significant variation in genome sizes among different accessions of *B. rapa* has been recently described [23].

#### Long-read genome assembly

We used 3 different assemblers: Redbean v2.3 (WTDBG, RRID:SCR_017225) (git commit 3d51d7e) [24], SMARTdenovo (SMARTdenovo, RRID:SCR_017622) (git commit 5cc1356) [25], and Flye v2.4.2 (Flye, RRID:SCR_017016) (git commit 5c12b69) [26] with all nanopore reads or subsets of reads composed of either the longest reads or those selected by the Filtlong [27] software (v0.2.0, git commit cf65a48) with default parameters (Supplementary Table S2) because it has been proven that it could be beneficial for the assembly phase to downsample the read coverage [3]. We used the following options as input to SMARTdenovo: “-c 1” to generate a consensus sequence, “-J 5000” to remove sequences smaller than 5 kb, and “-k 17” to use 17-mers as advised by developers in the case of large genomes.

Then, we selected 1 of the assemblies based not only on contiguity metrics such as N50 but also cumulative size. The Flye (longest reads) and SMARTdenovo (all reads) assemblies were very similar in terms of contiguity (N50 and N90), but we decided to keep the Flye assembly because its cumulative size was higher. The Flye assembler using the longest reads resulted in a contig N50 of 10.0 Mb and a cumulative size of 937.9 Mb.

Because nanopore reads contain systematic error in homopolymeric regions, we polished the consensus of the selected assembly 3 times with nanopore reads as input to the Racon [28] software (Racon, RRID:SCR_017642) (v1.3.2, git commit 5e2ecb7) and then 3 additional times using Illumina reads as input to the Pilon tool (Pilon, RRID:SCR_014731) (v1.2.3, git commit be801ec) [29], as recommended in a previous study [3]. Both tools were used with default parameters (Supplementary Table S3, Figs S2 and S3).

#### Long-range genome assembly

The genome map assemblies of *B. napus* Darmor-bzh have been generated using the Bionano Solve Pipeline version 3.3 and Bionano Access version 1.3 (Supplementary Tables S4–S6). The assemblies were performed using the parameters “non haplotype without extend and split” and “add Pre-assembly.” This parameter allows a rough assembly to be obtained that can be used as reference for a second assembly. We filtered out molecules smaller than 180 kb and molecules with <9 labeling sites (Supplementary Tables S4 and S5). The Bionano scaffolding workflow was launched with the nanopore contigs and the 2 Bionano maps (DLE and BspQI). As already reported [2], we found in several cases that the nanopore contigs were overlapping (based on the optical map) and this overlap was not managed by the hybrid scaffolding procedure. We corrected these negative gaps using the BiSCoT software [30] with default parameters (Supplementary Table S7 and Fig. S3).

#### Validation and anchoring of the *B. napus* Darmor-bzh assembly

**Figure 1.**
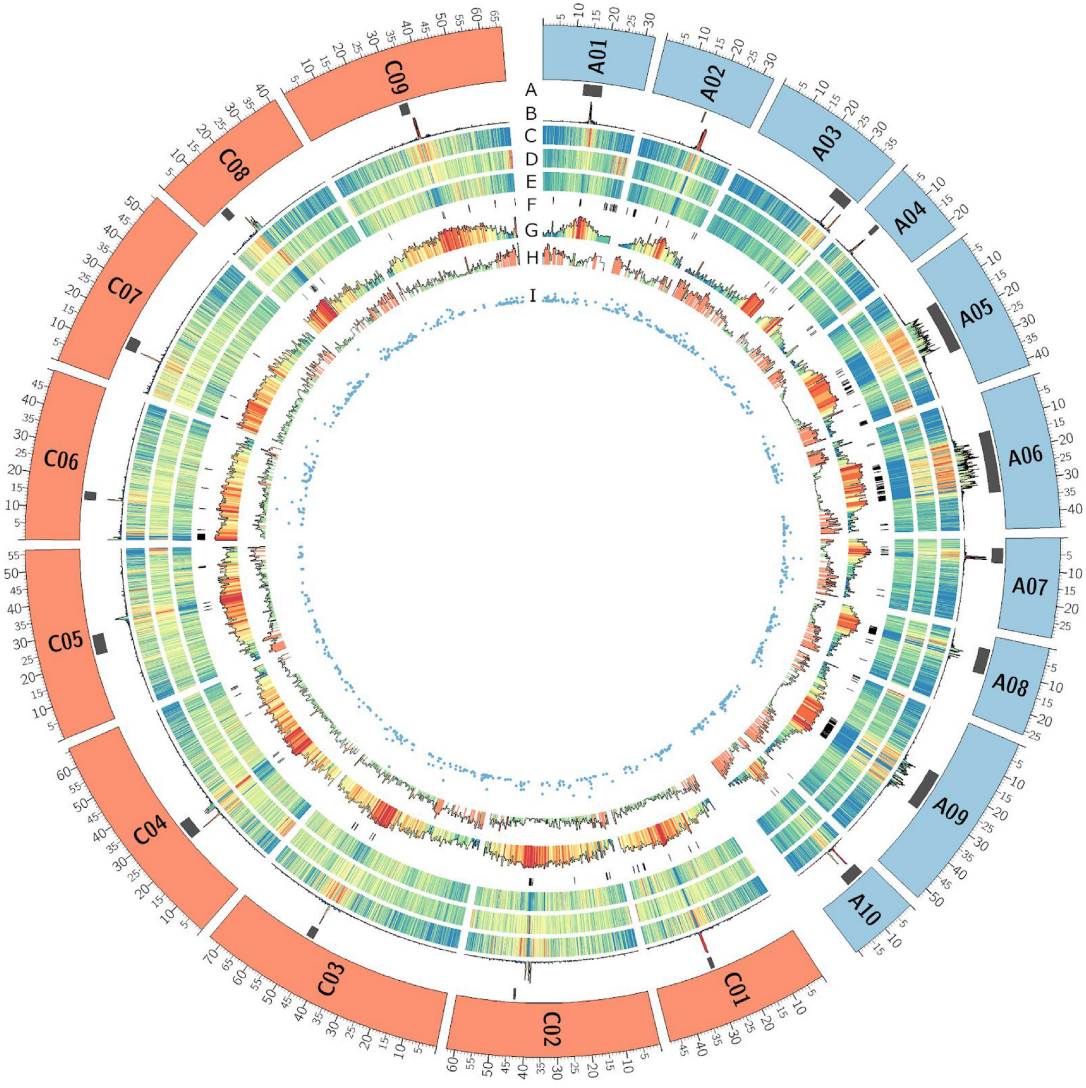
Genome overview of the 19 chromosomes of *B. napus* Darmor-bzh v10 (the 10 A chromosomes are in blue and the 9 C chromosomes in orange). A, Localization of centromere from flanking markers identified by Mason et al. [47]. B, Density of repeated pericentromere-specific sequences of *Brassica* allowing more precise localization of centromeres. C, Density of Gypsy elements. D, Density of Copia elements. E, Density of DNA transposon elements. F, Black boxes represent gaps in the Darmor-bzh assembly. G, Density of methylated CpG. H, Gene density. I, Scatter plots of RGAs density. All densities are calculated in 100-kb windows; blue and red colors in density plots indicate lower and higher values, respectively.

A total of 39,495 markers deriving from the Illumina 8K, 20K, and 60K arrays were genetically mapped on the integrated map, totaling 2777.7 cM. The sequence contexts of all single-nucleotide polymorphism markers that were genetically mapped were blasted against our *B. napus* Darmor-bzh assembly to validate the quality of our assembly and to help order and orient the scaffolds. Of these 39,495 genetically mapped markers, 36,391 were physically anchored on the final Darmor-bzh assembly (Fig. 1 and Supplementary Table S8). The genetic and physical positions were discordant for only 618 markers (0.02%) due to an inaccurate position on the genetic map (variation of a few centimorgans in almost all cases).

#### Identification of large misassembled inverted regions

**Figure 2.**
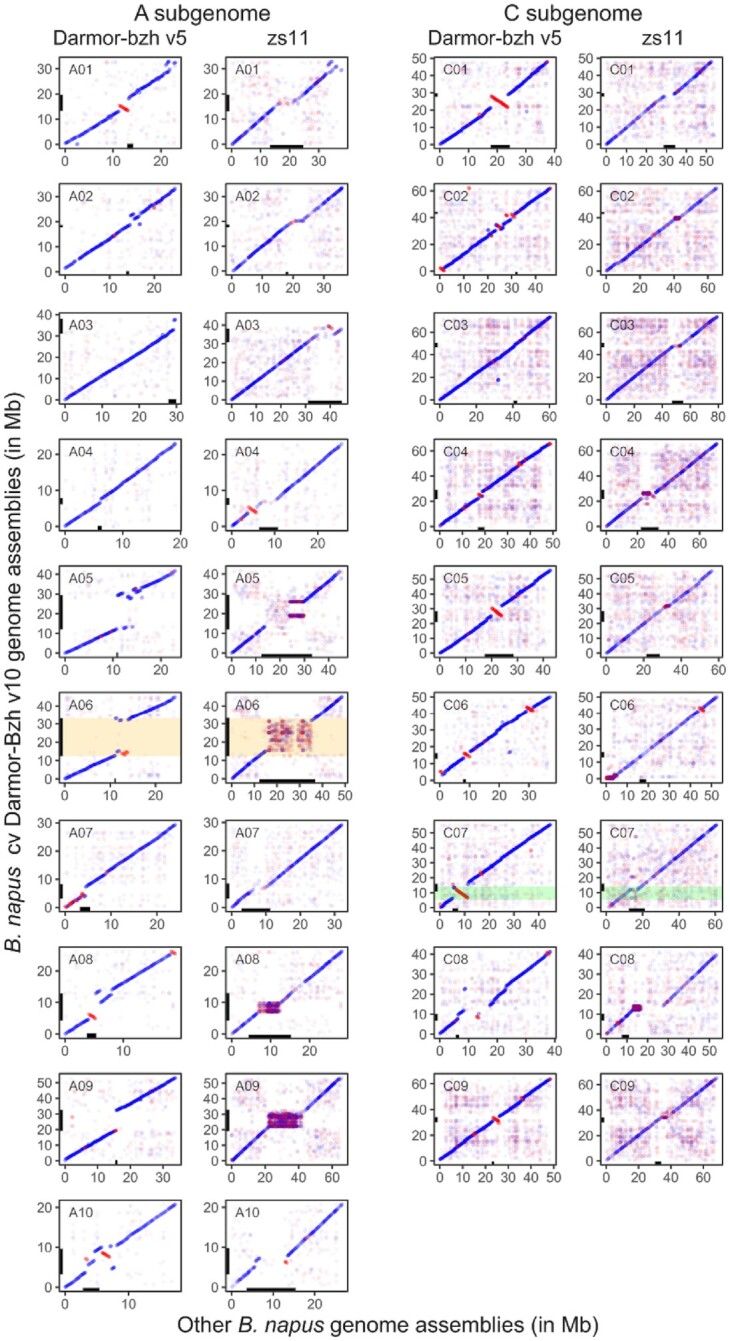
Genome-wide alignments of *B. napus* Darmor-bzh v10 (Y axis) with Darmor-bzh v5 and ZS11 genome assemblies (X axis). Each dot corresponds to syntenic regions of the genomes that are aligning with high confidence. Blue dots correspond to regions aligned in the correct orientation (forward strand) while red dots represent regions aligned in an inverted orientation (reverse strand). (Peri)centromeric regions are represented by black boxes on the X and Y axes. Some (peri)centromere flanking markers were not found in Zs11 owing to polymorphisms. The new assembly of a centromeric region in Darmor-bzh v10 compared to Darmor-bzh v5 is highlighted in orange for the A06 chromosome. An example of a large misassembled inverted region whose orientation has been corrected within the new Darmor-bzh v10 genome assembly is highlighted in green for the chromosome C07.

To compare this new *B. napus* Darmor-bzh assembly (v10) to the first published version (v5) as well as to the recent *B. napus* ZS11 genome assembly [11], we performed whole-chromosome alignments using nucmer from Mummer (MUMmer, RRID:SCR_018171) version 3.23 [31], followed by automatic plotting using ggplot (ggplot2, RRID:SCR_014601) version 3.3.0 in R (R Project for Statistical Computing, RRID:SCR_001905) version 4.0.0 [32]. Nucmer was used with default parameters, followed by delta-filter with the “-i 98 -l 1000" parameters. Using the output of nucmer and delta-filter described above, we subsequently identified large misassembled inverted (LMI) regions between the v5 and v10 genome assemblies. We used show-coords from Mummer with the parameters “-H -d -r -T," followed by a specific method to extract LMIs. Because the show-coords output consists in many small alignments from which we need to infer LMIs, we used an iterative process based on merging the coordinates of small consecutive inversions in larger ones, followed by filtering out the small inversions. The parameters were validated by visually and manually checking the resulting LMI coordinates against the chromosome-by-chromosome alignment scatter plots (Fig. 2). More precisely, we extracted LMIs from the show-coords output, followed by “bedtools merge -d 100 000" using bedtools (BEDTools, RRID:SCR_006646) version 2.29.2 [33] to merge the coordinates of inversions separated by ≤100 kb, then we selected inversions of size superior to 100 kb, subsequently merged the inversions separated by 300 kb with bedtools, selected inversions >700 kb, and finally merged inversions separated by 1 Mb with bedtools.

### Genome annotation

#### Detection of modified bases

The nanopore technology has the advantage of reading native DNA molecules and can distinguish modified from natural bases because they affect the electrical current flowing through the pore differently [34]. The nanopore reads were mapped using minimap2 [35] (Minimap2, RRID:SCR_018550) (version 2.17-r941 with the “-x map-ont” parameter). We called the 5-methylcytosine (5mC) in the CpG context of the whole genome of *B. napus* Darmor-bzh and computed frequencies using nanopolish version 0.12.5 with a minimum log-likelihood threshold of 2. CpG positions showing a frequency >50% and covered by ≥4 reads were considered methylated. The proportion of methylated CpG per 100 kb across the genome is shown in Fig. 1.

#### Correction of direct RNA reads

Direct RNA reads were corrected using TALC (version 1.0) [36] with default parameters (except -k = 21) and Illumina reads from leaf and root samples of *B. napus* downloaded from the EBI database (ERX397788 and ERX397800). Before the correction step, raw reads were masked using DustMasker (version 1.0.0 from the BLAST 2.10.0 package) [37], and we kept reads with ≥150 unmasked positions and ≥75% of unmasked bases. From 10,416,515 input reads, we retained 9,099,437 nanopore reads and TALC successfully corrected 8,523,238 reads (93.7%) with a mean length of 645 bp (Supplementary Table S11).

#### Transposable element annotation

Transposable elements (TEs) were annotated using RepeatMasker (RepeatMasker, RRID:SCR_012954) (version 4.1.0 with default parameters) [38] and TE libraries from [8]. We masked nearly 54% of the genome, and long terminal repeat (LTR) Copia and Gypsy elements are the most abundant (15.5% and 13.0%, respectively). The 3 Darmor-bzh assembly releases and the 14 other *Brassica* genome assemblies were masked using the same procedure (Table 5).

#### Gene prediction

Gene prediction was performed using several proteomes: 8 from other genotypes of *B. napus* [11] (Westar, Zs11, QuintaA, Zheyou73, N02127, GanganF73, Tapidor3, and Shengli3), *Arabidopsis thaliana* (UP000006548), the *B. napus* pan-annotation [39], resistance gene analogs (RGAs) from *Brassica* [40], and the 2014 annotation of Darmor-bzh [8]. Regions of low complexity in genomic sequences were masked with the DustMasker algorithms (version 1.0.0 from the blast 2.10.0 package) [37]. Proteomes were then aligned on the genome in a 2-step strategy. First, BLAT [41] (BLAT, RRID:SCR_011919) (version 36 with default parameter) was used to quickly localize corresponding putative regions of these proteins on the genome. The best match and the matches with a score ≥90% of the best match score have been retained. Second, alignments were refined using Genewise [42] (GeneWise, RRID:SCR_015054) (version 2.2.0 default parameters), which is more accurate for detecting intron/exon boundaries. Alignments were kept if >75% of the length of the protein was aligned on the genome.

In addition, we used the error-corrected direct RNA reads produced using the PromethION device. As already reported [43], the detection of splicing sites using raw reads is difficult. For example, on a subset of 1,000 reads, the proportion of GT-AG splicing sites is 6% lower in the raw reads compared to the corrected reads (Supplementary Table S12). Error-corrected reads were aligned on the genome in a 2-step strategy. First, BLAT (version 36 with default parameters) was used to quickly localize corresponding putative regions of these RNA reads on the genome. The best match for each read was retained and a second alignment was performed using Est2Genome [44] (version 5.2 default parameters) to detect exon boundaries. From the 8,523,238 corrected reads, we retained 4,651,118 alignments that covered ≥80% of the read with ≥95% identity. To reduce the redundancy, we clustered the alignments on the genomic sequence using bedtools (version v2.29.2–17-ga9dc5335) [33] and kept for each cluster the alignment with the highest score. The 70,904 resulting alignments (mean identity of 97.5% and 4.07 exons per alignment) were used as input for the gene prediction.

All the transcriptomic and protein alignments were combined using Gmove (Gmove, RRID:SCR_019132) [45], which is an easy-to-use predictor with no need of a pre-calibration step. Briefly, putative exons and introns, extracted from alignments, were used to build a graph, where nodes and edges represent, respectively, exons and introns. Gmove extracts all paths from the graph and searches open reading frames that are consistent with the protein evidence. Finally, we decided to exclude single-exon genes predicted by RNA-Seq only and composed of >80% of untranslated regions. Following this pipeline, we predicted 108,190 genes with 4.55 exons per gene on average. Quality of the gene prediction was estimated using the single-copy orthologous gene analysis from BUSCO v4 (BUSCO, RRID:SCR_015008) [46] with Brassicales version 10, which contains 4,596 genes (Table 1 and Supplementary Fig. S3).

#### Correspondence between the genes of the v5 and v10 assemblies

We calculated a correspondence table between the predicted genes on the v5 and v10 assemblies. To that purpose, we aligned all the v5 proteins against all the v10 proteins using diamond (DIAMOND, RRID:SCR_016071) (version 0.9.24 with the following parameters: “–more-sensitive -e 0.00001”). The best reciprocal hits were selected if they came from the same chromosome in both assemblies, and used as anchors. We then enriched the anchored genes using synteny and by filtering hits on the basis of percent identity (>80%) and sequence coverage (>80% of the target or the query). Using this strategy (M1), we were able to find a correspondence for 73,560 of the 101,040 genes of the v5 assembly (72.8%). Owing to the importance of such information for the Brassica community, we decided to use a second method (M2) to increase the number of genes with a correspondence. For this latter method, we first performed a reciprocal BlastP (NCBI BLAST, RRID:SCR_004870) (blastp v2.9.0 with the parameters “-evalue 1e-20”) between the v5 and v10 proteins, then only conserved the blast hits with a minimum percentage of identity of 95%, an alignment length of 50%, and if they were on the same chromosome (scaffolds accepted). Using this second method, we identified approximately the same number of genes with a correspondence (72,314 v5 proteins). When comparing the results from these 2 methods, we observed that 85% were similar to both methods (62,514), ∼14% were found in only 1 method (11,032 and 9,800 specific to the first and second method, respectively), and 0.6% were discordant (450). In this article, we decided to provide the most complete correspondence table that derives from both analyses (82,441 proteins after removal of the 450 discordant results). In this supplementary table (Supplementary Table S18), we indicate which method(s) (M1 and/or M2) allowed us to identify the correspondence between v5 and v10 proteins. Any updates to this table will be available at Genoscope [60].

#### Localization and annotation of pericentromeric regions

We retrieved the markers flanking each centromere of *B. napu*s chromosomes [47] and aligned them against the different assembly versions of *B. napus* Darmor-bzh (and also Zs11) to determine the improvement of the highly repeated pericentromeric regions. The (peri)centromere localization was also searched using 6 (peri)centromere-specific repeat sequences of *Brassica* (CentBr1, CentBr2, CRB, PCRBr, TR238, and TR805) and by BLASTn [48] (version 2.9.0, with e-value 10–6) against the Darmor-bzh v10 genome sequence. The length and number of genes present in the different versions were compared.

#### Annotation of the RGAs

We identified the RGAs in Darmor-bzh v5 and v10 gene annotations using RGAugury (commit 57d58f887ad8c70e5d4c619a3d5e207158822819) [49] and the following command: “RGAugury.pl -p {peptides.fa} -e 1e-5 -c 10 -pfx {output}." The latest versions of all the databases and tools used by RGAugury were downloaded as of April 2020 following the installation instructions provided in the RGAugury online repository [50].

Briefly, this pipeline lets us identify different major RGA families (NBS encoding; RLP: membrane-associated receptor-like proteins; RLK: surface-localized receptor-like protein kinases). On the basis of the presence or absence of some domain structures (CC: coiled-coil; TIR: Toll/interleukin 1 receptor; NB-ARC: nucleotide binding site activity regulated cytoskeleton; LRR: leucine-rich repeat), the NBS-encoding proteins were subdivided into 8 different categories [51]. Finally, all the putative RGA proteins identified in Darmor-bzh v5 were blasted against those identified in this new assembly to establish a correspondence between these 2 versions, but also to identify newly annotated RGA genes in Darmor-bzh v10.

### An improved version of the Darmor-bzh genome assembly

#### Comparison with existing assemblies and annotations

**Figure 3.**
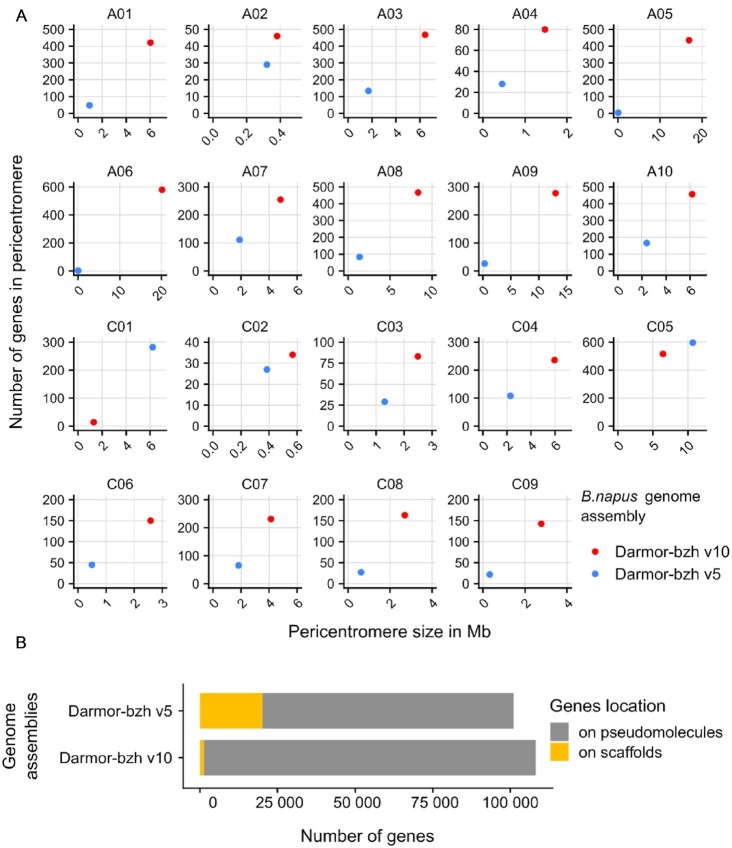
Improvements of the *B. napus* Darmor-bzh v10 genome assembly and annotation compared to Darmor-bzh v5 by the use of long-read sequencing. A, Genome-wide pericentromere size (in Mb) and gene content using centromere flanking markers (Mason et al. [47], refer to methods). The Darmor-bzh v10 genome assembly now harbors larger pericentromeres with more annotated genes than in the previous genome. B, Repartition of gene locations on chromosome and unplaced scaffolds.

The 2 published releases of Darmor-bzh [8, 9] were generated using 454 and Illumina reads and have a low contiguity (Table 1). These fragmented assemblies were difficult to organize at the chromosome level, and as a result only 553 and 690 Mb of sequences were, respectively, anchored on the 19 chromosomes. In comparison, the 19 chromosomes of the ONT assembly contain 849 Mb. The gene completion (BUSCO score) of the first release and of this one is similar (97.7% and 98.6%), showing that long-read assemblies mainly affect the repetitive compartment of the genome. However, 98.8% of the genes are now placed on their respective chromosomes rather than on unplaced scaffolds; in comparison only 80% of the predicted genes were located on pseudomolecules in Darmor-bzh v5 (Fig. 3B). These improvements will help the *Brassica* community to identify the genes underlying agronomic traits of interest found using quantitative genetics.

We compared the short- and long-read assemblies and observed large newly assembled regions comprising centromeric sequences (Fig. 2, highlighted in orange on chromosome A06). By aligning the flanking markers of *B. napus* centromeres [47], we were able to identify the positions of the approximative pericentromeric regions in Darmor-bzh v5 and the present Darmor-bzh v10 assembly (Supplementary Table S14 and Fig. 1). Thereafter, we compared the lengths and gene contents of these regions between Darmor-bzh v5 and v10 (Fig. 3A). On average, we observed an increase of sequence assembly in pericentromeric regions by 80-fold, with some extreme cases such as the A06 pericentromere, which is 1,180 times larger in Darmor-bzh v10 compared to Darmor-bzh v5. Concerning pericentromeric gene content, we show an average increase of 24.4-fold, with the A06 pericentromere showing 290 times more genes in Darmor-bzh v10 compared to Darmor-bzh v5. There were only 2 exceptions (C01 and C05), where we identified a decrease in pericentromere size and gene content in Darmor-bzh v10 compared to Darmor-bzh v5. However, these results may in fact be attributable to misassembled inverted regions in Darmor-bzh v5, which changed the position of the markers on which our detection of pericentromeric regions is based. Indeed, comparison of the different *B. napus* Darmor-bzh assemblies allowed to notably identify 27 LMIs (>700 kb) in Darmor-bzh v5 that are now correctly assembled in the Darmor-bzh v10 assembly. These misassembled regions were validated using the new ZS11 assembly [11] as a control (Fig. 2, highlighted in green on the C07 chromosome). The 27 detected LMIs total 3,494 genes and 45 Mb in Darmor-bzh v10, with the largest located on chromosome C01, measuring 6 Mb and encompassing 276 genes. We provide a list of the LMI regions detected in Darmor-bzh v5 and corrected in Darmor-bzh v10, alongside the corresponding genes in these regions (Supplementary Table S15).

All these analyses highlight the improvements of the Darmor-bzh v10 genome assembly thanks to the use of ONT long-read sequencing. Overall, this high-quality genome assembly of *B. napus* Darmor-bzh will be particularly useful to the *Brassica* community to decipher genes underlying agricultural traits of interest.

#### Comparison of available *Brassica* genome assemblies

We downloaded the 14 available *Brassica* long-read assemblies (and annotations) and computed usual metrics. For each assembly, contigs were generated by splitting sequences at each gap and the gene completion was evaluated on predicted genes using BUSCO [46] and the conserved genes from the Brassicales database (N = 4,596 genes). Not surprisingly the gene content is high, between 97.9% and 98.8% for all 10 *B. napus* assemblies (Table 3 and Supplementary Table S9), and is lower for the diploid species (due to the presence of a single genome), except for *B. rapa* Chiifu, which has a surprising number of genes (>60,000). Likewise, the repetitive content of these 10 genomes is similar, between 54% for Darmor-bzh and 60% for no2127. Concerning the diploid genomes and as already reported [52], *B. oleracea* (C) genome assemblies contain more repetitive elements than the *B. rapa* (A) and *B. nigra* (B) genome assemblies (Table 5).

The main observed differences are the contig length, which affects the number of gaps in each assembly (from 268 gaps for Darmor-bzh to 5,460 gaps for Zs11), and the number of anchored bases (from 765 Mb for Express617 to 961 Mb for Zs11). We further investigated these 2 differences, which we believe to be related to the technologies used.

**Figure 4.**
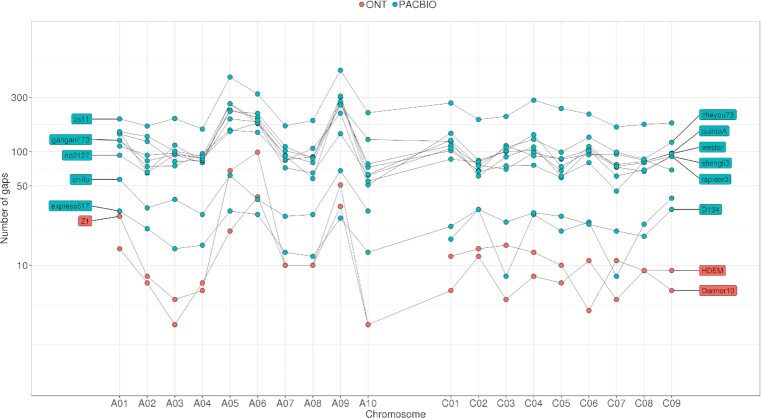
Distribution of the number of gaps per chromosome in *Brassica* genomes. Each dot represents the number of gaps in a given chromosome and genome assembly. PacBio assemblies are in blue and ONT assemblies in orange.

**Figure 5.**
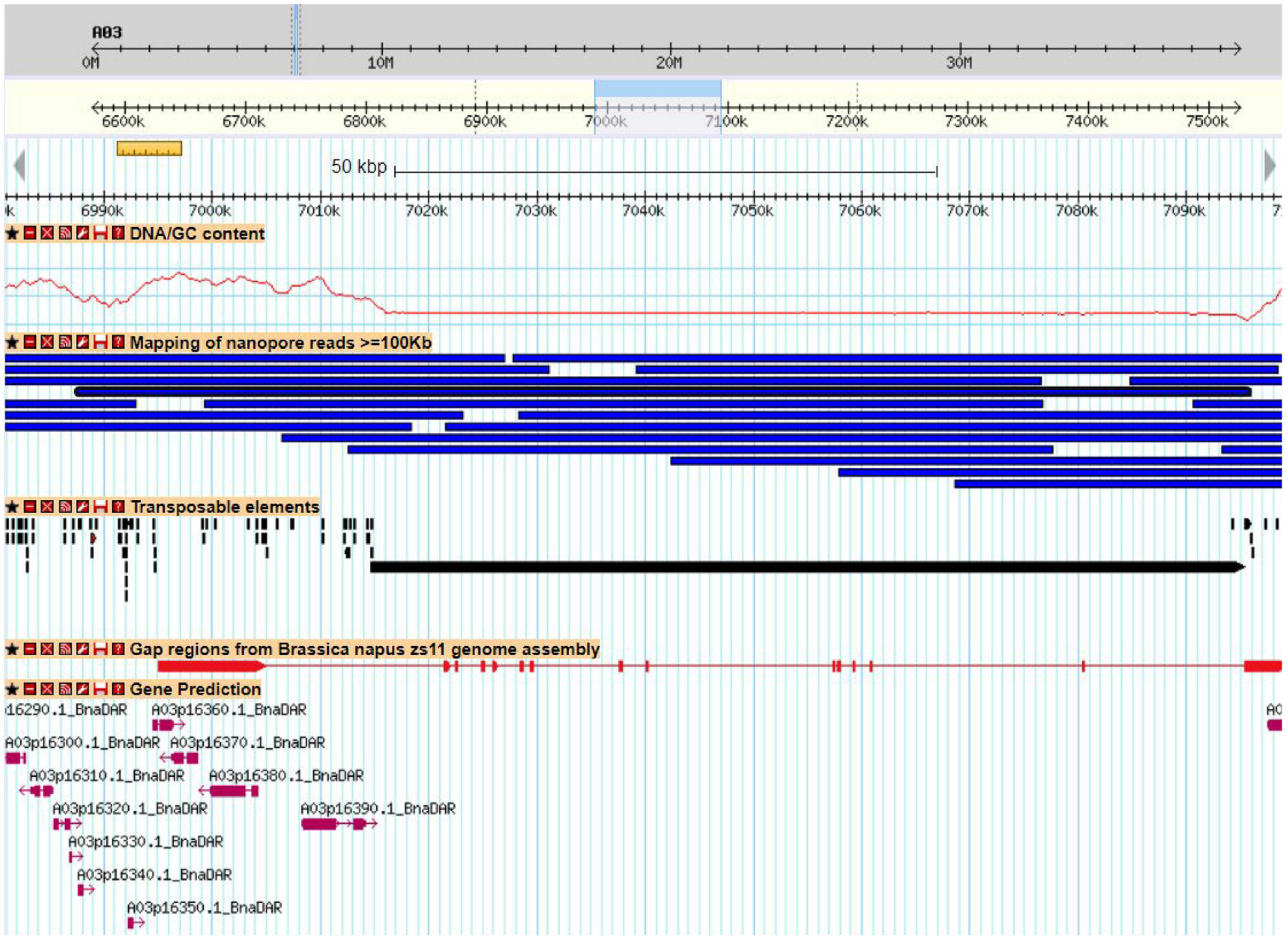
Example of a genomic region of *B. napus* Darmor-bzh assembly that corresponds to a region that contain a gap in the Zs11 genome assembly. First track represents the GC content. ONT reads (longer than 100 kb) are in the second track (blue boxes), and a 115-kb read that spanned the whole region is surrounded by a black box. Transposable elements are shown as black boxes, and the region absent from Zs11 contains a large LINE element (80,707 bp). The alignment of the Zs11 sequence (20 kb around the 500-bp gap) is represented by red boxes, with thin red lines representing missing sequences in the Zs11 genome assembly that are present in Darmor-bzh v10 (gaps). Predicted genes are in the last track, in purple.

We observed a significant difference in contiguity when comparing ONT and PacBio assemblies. The 11 *Brassica* genomes sequenced using PacBio have a contig N50 between 1.4 and 3.6 Mb whereas the 4 ONT assemblies have a contig N50 >5.5 Mb. The ability of the nanopore technology to sequence large fragments of DNA appears to be an advantage for assembling complex genomes. In the case of Darmor-bzh, we were able to obtain >50,000 reads longer than 100 kb (representing 6× of coverage). This dataset allowed us to generate a contiguous assembly with only 268 gaps. As a comparison, we found 5,460 gaps in the PacBio assembly of the Zs11 genotype. This difference is observed in all the A and C genomes and subgenomes that have been sequenced using long reads and may be directly related to the longer length of ONT reads (Fig. 4). Although the number of gaps is higher in the PacBio assemblies, the total number of N's is lower at least for the assemblies that have been organized with Hi-C data. Indeed, the Hi-C pipelines generally order contigs and add a fixed gap size between 2 contigs. We investigated the 500-bp gaps in the Zs11 assembly and aligned their flanking regions on the Darmor-bzh assembly using blat (version 36 with default parameters) [41]. We applied stringent criteria (alignment on the same chromosome, score >4,000, alignment of ≥1 kb of each of the flanking regions, and alignment covering <100 kb on the Darmor-bzh assembly) and we found a location for 367 of the 5,460 gaps of Zs11. These 367 regions covered >19 Mb of the Darmor-bzh assembly with a mean size of 52 kb. Even if these regions could be different between the 2 genotypes, we examined their content in TEs and found a high proportion of bases annotated as repetitive elements (82.9%) and a different distribution of the classes of elements compared to the whole genome (Supplementary Table S13). We observed a higher proportion of Copia elements, but especially LINE and satellite elements. For example, we observed large LINE elements (>50 kb) as shown in Fig. 5, where ultralong nanopore reads covered this element and avoided a contig breakage in this area.

Interestingly, A chromosomes seem more difficult to assemble (Fig. 4). Even if A genome is smaller than C genome and contains fewer TEs (Table 5), the number of contigs (and consequently the number of gaps) in A genome, or subgenome, is twice the number of contigs in C genome, or subgenome (gap track on Figs 1 and 4). Chromosomes A05, A06, and A09 appear to be the most challenging to assemble owing to the high content of TEs in their centromeric regions. They alone contain 43% of the gaps (and 51% of the undetermined bases) present on the 19 chromosomes. However, these chromosomes have been shown to be highly variable in length across *B. rapa* genotypes [23].

The second difference observed between all these assemblies is the proportion of anchored sequence, which is higher for the genome assembly that has been organized using Hi-C data (95.0%, Zs11) although it is the most fragmented (Supplementary Table S10). Indeed, the organization of small contigs is complicated using optical or genetic maps because it requires a sufficient number of restriction sites or markers. This aspect probably explains the small proportion of the Express617 assembly (82.7%) that has been anchored on the 19 chromosomes. The other genomes have been organized using comparative genomics (synteny with the Zs11 assembly). Although it is a convenient method, it may generate false organization and the proportion of anchored bases is variable, from 89.1% to 92.7%, depending on the conservation between the 2 genomes. We think that if the assembly is highly contiguous, optical maps have the important advantage of estimating the size of gaps, which remains a limitation when using Hi-C data.

### Sequencing of native RNA molecules

The sequencing of RNA is traditionally performed using the Illumina technology, where the RNA molecules are isolated and then reverse transcribed to complementary DNA, which is more stable and allows amplification to be performed prior to the sequencing. The ONT technology is the first to propose the sequencing of native RNA molecules without the RT step. One of the main advantages is a better quantification of gene expression when compared to methods that require RT [43]. Moreover, the sequencing of native RNA molecules preserves modified bases and theoretically allows them to be detected [53, 54]. Here, we sequenced a mix of leaf and root samples using the PromethION device and generated 10,416,515 reads with a mean size of 559 bp. This dataset was corrected using TALC as described previously and corrected reads were used in the gene prediction workflow (Supplementary Table S11).

#### Alternative splicing events

**Figure 6.**
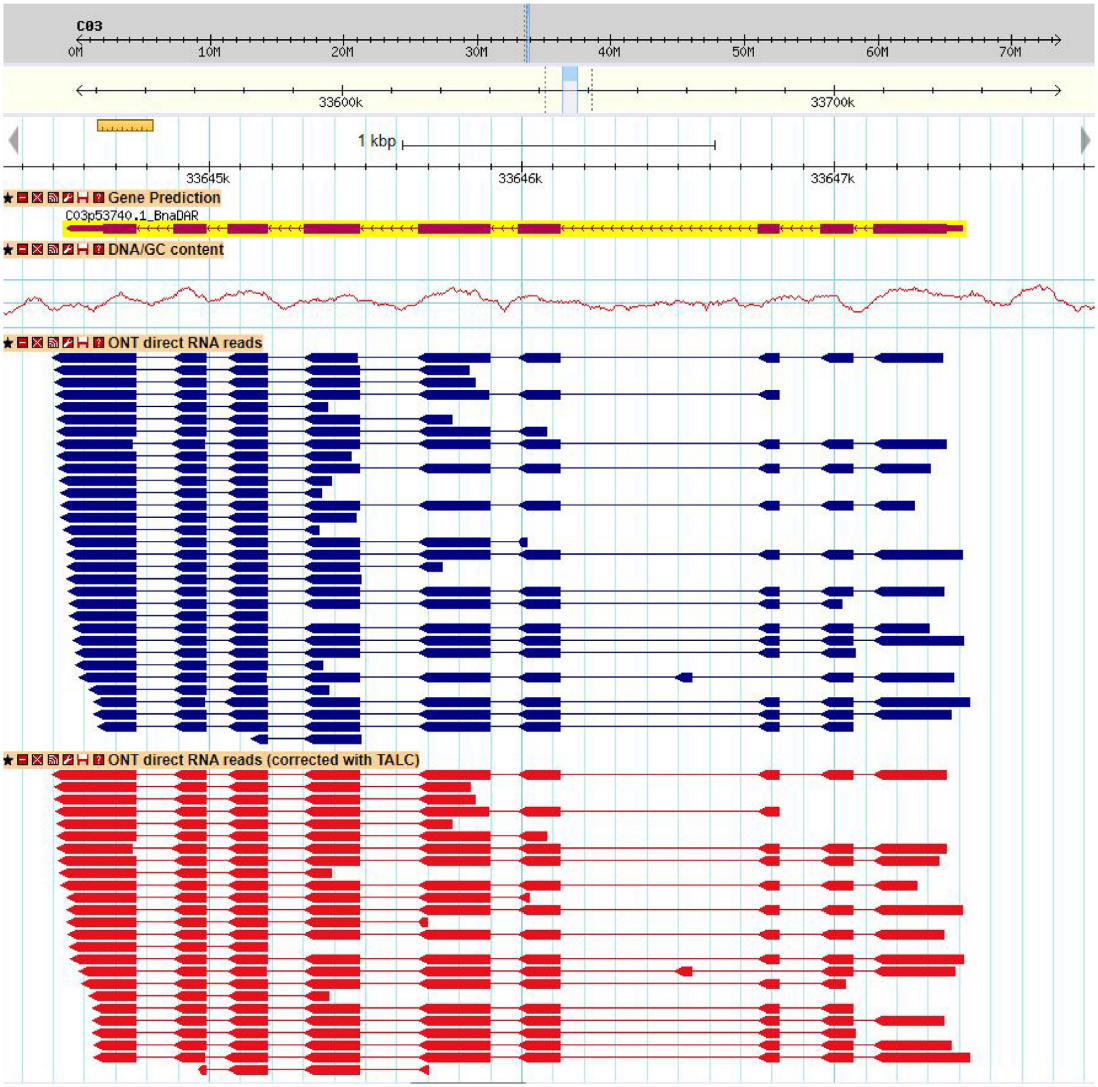
Example of a splicing event detected using long reads. The exon in the third intron of the gene prediction (C03p53740.1_Bna_DAR, Gene prediction track) is mutually exclusive with the third exon. This alternative splice form is detected by a single nanopore read (blue track) and maintained by the TALC error correction (red track).

Independently, we detected alternative splicing events, as skipping exons and intron retentions. Aligning noisy reads makes it difficult to accurately detect splice sites and therefore 5′ and 3′ alternative splice sites. Raw reads were aligned on the genome in a 2-step strategy. First, BLAT (version 36 with default parameters) [41] was used to quickly localize corresponding putative regions of these RNA reads on the genome. The best match for each read was selected and a second alignment was performed using Est2Genome [43] (version 5.2 with default parameters). Alignments with an identity percent >90% were retained, and we used bedtools to extract intron retention and skipping exons. We searched for predicted introns that were entirely covered by an exon of a nanopore read. Using this conservative approach, we were able to identify 30,397 events distributed across 18,204 genes (16.8%). Chalhoub et al. [8] detected an intron retention in 29% of the 101,040 annotated genes. Using the same approach and a coverage of ≥10 Illumina reads, we found intron retention in 21.2% of the genes. This proportion increased to 24.4% and 30.3% if lower coverage was used (8 and 5, respectively). We compared the intron retention predicted by Nanopore and Illumina reads and found only 56% in common and therefore 44% of the events are specific to Nanopore reads. However, using lower coverage for prediction with Illumina data increased the proportion of common events (62% with coverage >8 and 73% with coverage >5), indicating that Nanopore reads can also detect rare events. However, the smaller number of events detected using the Nanopore reads may indicate that sequencing 10M reads is not sufficient and there is a need to sequence the RNA sample more deeply. In addition, we detected skipping exons (reads with 1 exon that is entirely covered by an intron, smaller than 3 kb to avoid mapping errors) in 3% of the 108,190 annotated genes. For example, by inspecting mutually exclusive exons, we were able to find an already described event that is conserved between *Brassica* and *A. thaliana* [55] (Fig. 6). It is obvious that the sequencing of RNA using long reads will allow detection of co-occurrence of splicing events, which is difficult if not impossible to do with short reads (Supplementary Fig. S4).

#### Comparison of the resistance gene analogs catalogs

**Figure 7.**
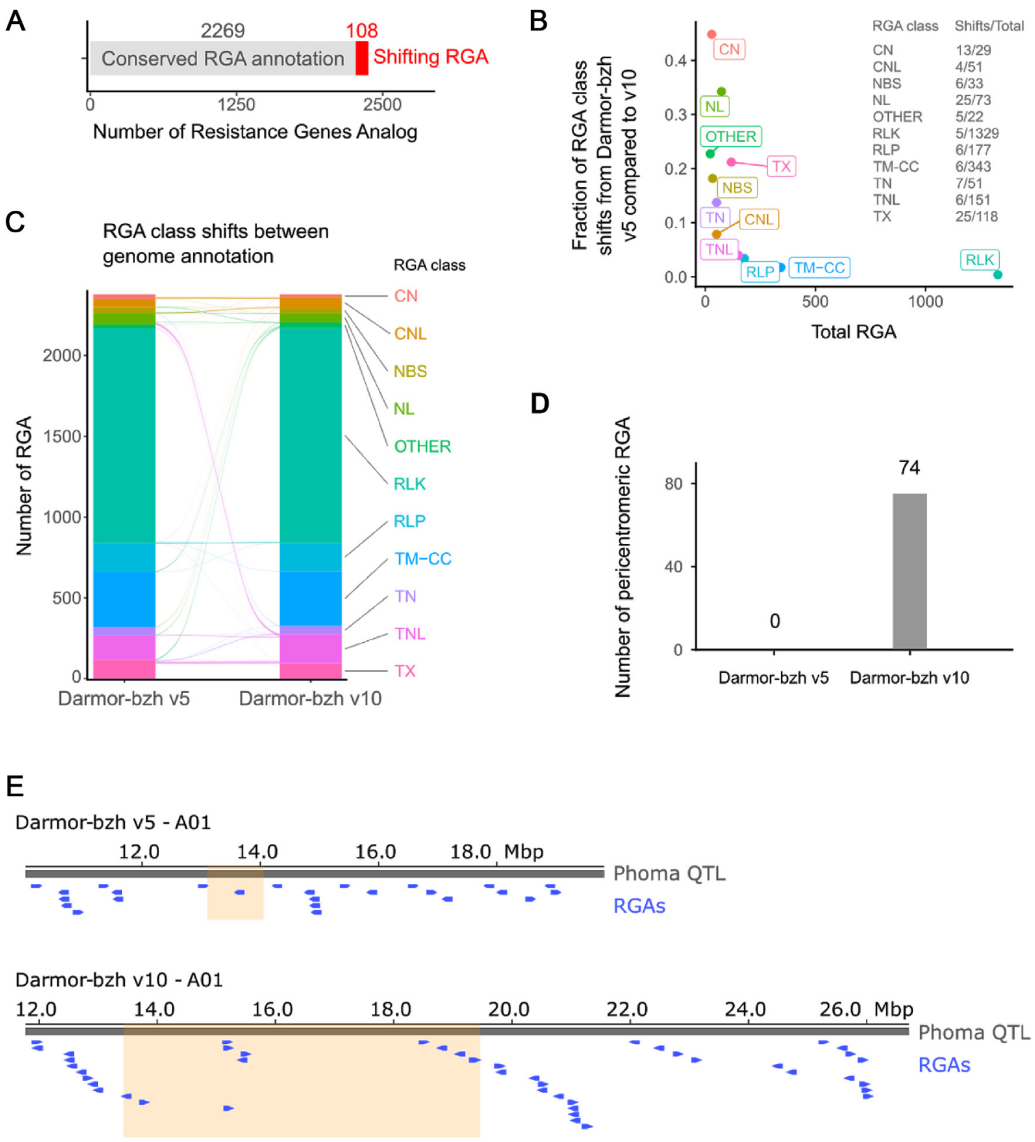
Resistance gene analogs (RGA) annotation improvement in *B. napus* Darmor-bzh v10 using long-read direct RNA sequencing. A, Conservation of the RGA categories between Darmor-bzh v5 and v10 genome annotations. Shifting RGAs are genes whose sequence is matching but that have been annotated in a different RGA class by RGAugury between the v5 and v10 genome annotations. Such shifting RGAs only represent a small fraction of the annotated RGAs. B, Total number of RGAs plotted against the ratio of shifting RGAs over total RGAs by RGA class. CN and NL RGAs are the categories whose annotation shifted the most between Darmor-bzh v5 and v10. A detail of the raw data used to compute the ratio is available on the right part of the plot. C. Detail of the RGA class shifts between Darmor-bzh v5 and v10 made using ggalluvial [56]. The size of the arcs is proportional to the number of shifting RGAs from 1 RGA class to another between the 2 annotations. E, Genome browser snapshot using pyGenomeTracks [57] of the phoma canker resistance QTL (grey) in Darmor-bzh v5 (top) and Darmor-bzh v10 (bottom). RGA genes (blue) and pericentromeric regions (orange) are displayed.

Using the RGAugury pipeline [47], we annotated 2,788 and 2,952 resistance gene analogs (RGAs) in the gene catalogs of Darmor-bzh v5 and v10, respectively (Supplementary Tables S16 and S17). In Darmor-bzh v5 only 83.5% of the RGAs were located on anchored scaffolds, while we identify 99.4% of the RGAs on chromosomes in Darmor-bzh v10. On average 153 ± 46 RGAs are located on each pseudomolecule in Darmor-bzh v10 compared to 122 ± 33 in Darmor-bzh v5 (rounded mean ± standard deviation). Overall, more RGAs were annotated in the Darmor-bzh v10 annotations in almost all categories, thanks to the long-read sequencing. Most of the RGAs found in Darmor-bzh v5 were conserved in Darmor-bzh v10 (81.4%, Table 4 and Fig. 7A). However, we observed that 108 RGAs' annotation shifted from 1 RGA class to another between the 2 versions owing to the identification of additional domains in these proteins (Fig. 7A). Between the Darmor-bzh v5 and Darmor-bzh v10 annotations, the CN and NL RGA classes shifted the most to CNL (CC, NB-ARC, and LRR domains, Fig. 7B and C), which display 1 additional domain compared with CN (CC and NB-ARC domains) or NL (NB-ARC and LRR domains) RGAs. In addition, we identified dozens of examples where a few adjacent RGA genes in Darmor-bzh v5 now correspond to a single gene in Darmor-bzh v10.

**Table 4.** Resistance gene analogs categories and repartition between chromosomes and unplaced scaffolds in *B. napus* Darmor-bzh v5 and v10 genome assemblies

RGA Location	RGA Class											Total
	NBS	CNL	TNL	CN	TN	NL	TX	Others	RLP	RLK	TM-CC	
Darmor-bzh v5 RGA												
Genome-wide	37	53	164	33	60	80	143	24	274	1,502	418	2,788
Pseudomolecules	27	45	144	27	54	67	112	22	231	1,259	340	2,328
Scaffolds	10	8	20	6	6	13	31	2	43	243	78	460
Darmor-bzh v10 RGA												
Genome-wide	45	71	189	22	58	85	128	29	285	1,569	471	2,952
Pseudomolecules	45	71	184	22	57	84	128	29	282	1,561	471	2,934
Scaffolds	0	0	5	0	1	1	0	0	3	8	0	18

**Table 5.** Repetitive element content of the *Brassica* long-read assemblies (10 polyploid *B. napus*, 2 diploid *B. rapa*, 1 diploid *B. nigra*, and 2 diploid *B. oleracea* genomes)

	Total length (Mb)	Masked proportion (%)	LTR Copia (%)	LTR Gypsy (%)	DNA CMC-EnSpm (%)	LINE (%)	RC Helitron (%)	DNA TcMar (%)	DNA MULE-MuDR (%)
Darmor-bzh	924	53.86	15.55	13.05	6.66	6.37	5.26	2.05	1.93
Westar	1,008	58.91	16.88	13.38	6.26	6.92	4.87	1.92	1.77
Express617	925	56.32	15.92	12.46	6.54	6.63	5.22	2.05	1.88
Tapidor3	1,014	59.01	16.49	13.53	6.17	7.05	4.86	1.91	1.76
Shengli3	1,002	58.50	16.15	13.16	6.16	6.97	4.86	1.92	1.78
QuintaA	1,004	58.60	16.40	13.23	6.29	7.03	4.93	1.93	1.80
No2127	1,012	59.85	17.08	13.74	6.23	7.02	4.77	1.91	1.72
GanganF73	1,034	58.76	16.60	13.48	6.23	6.86	4.82	1.91	1.75
Zheyou73	1,016	59.06	16.49	13.59	6.28	7.04	4.87	1.91	1.79
Zs11	1,010	58.62	16.59	13.37	6.36	6.92	4.88	1.95	1.78
Z1	402	46.95	13.22	13.23	2.88	5.16	4.37	1.39	1.53
Chiifu	353	48.46	12.89	13.18	3.29	5.60	5.15	1.63	1.82
NI100	506	53.70	16.51	22.59	4.08	4.93	3.17	1.42	1.79
D134	530	57.88	15.61	13.23	9.16	6.24	5.62	2.56	2.08
HDEM	555	57.14	16.06	12.68	8.74	6.31	5.42	2.46	1.99

Because pericentromeric region assembly was greatly improved in Darmor-bzh v10, we compared the number of RGAs in such regions between the 2 assemblies and found 74 RGAs in Darmor-bzh v10 compared with no RGAs in Darmor-bzh v5. To further highlight the interest of assembling such regions using ONT technologies, we compared the size and number (including RGA) of a resistance quantitative trait locus (QTL) to phoma stem canker overlapping the A01 centromere [47, 50]. In this new assembly, we were able to identify 45 candidate RGAs compared to 29 in Darmor-bzh v5 (Fig. 7E). This finding opens new ways to understand the mechanism underlying this QTL.

## Conclusions

In this study, we generated the most contiguous *B. napus* genome assembly to date thanks to ONT long reads and Bionano optical maps. In addition, the ONT dataset allows the detection of modified bases, such as 5mC, without any specific library preparation. We have shown that the generation of ultra-long reads is a game-changer for assembling complex regions, composed of TEs, common in plant genomes. Consequently, the combination of ultralong reads and optical maps is today a method of choice for generating assemblies of complex genomes. It is important to keep in mind that nanopore technology is in constant evolution, in particular base-calling software, which makes it possible to improve an assembly starting from existing data. In addition, we have predicted genes in this new assembly using direct RNA sequencing and have used this original dataset to detect splicing events and show that this technology can be used to discover complex events, such as the co-occurence of events as mutually exclusive exons. This improved version of the *B. napus* Darmor-bzh reference genome and annotation will be valuable for the *Brassica* community, which has now been working with the short-read version for almost 6 years, particularly to decipher genes underlying agricultural traits of interest.

## Additional Files

Supplementary Figure S1. Kmer spectrum of *Brassica napus* Darmor-bzh.

Supplementary Figure S2. KAT analysis of the *Brassica napus* Darmor-bzh assembly.

Supplementary Figure S3. Schematic diagram summarising the genome assembly, scaffolding and annotation steps.

Supplementary Figure S4. Example of a difficult to detect transcript isoform with short-reads.

Supplementary Table S1. Statistics of the sequencing dataset for *B. napus* Darmor-bzh.

Supplementary Table S2. *B. napus* Darmor-bzh ONT assembly statistics.

Supplementary Table S3. Iterative polishing of the Flye assembly.

Supplementary Table S4. *B. napus* Darmor-bzh bionano dataset (raw molecules).

Supplementary Table S5. *B. napus* Darmor-bzh bionano dataset (filtered molecules).

Supplementary Table S6. *B. napus* Darmor-bzh optical maps.

Supplementary Table S7. *B. napus* Darmor-bzh hybrid scaffolding and polishing.

Supplementary Table S8. *B. napus* Darmor-bzh chromosomal organization.

Supplementary Table S9. Statistics of the *Brassica* long-reads assemblies.

Supplementary Table S10. Statistics of the *B. napus* long-reads assemblies.

Supplementary Table S11. Statistics of direct RNA nanopore reads.

Supplementary Table S12. Mapping of a sample of 1,000 reads using blat and est2genome.

Supplementary Table S13. Repetitive content of the *B. napus* Darmor-bzh genome assembly compared to the regions present in Darmor-bzh but absent from *B. napus* zs11.

Supplementary Table S14. *B. napus* Darmor-bzh pericentromeric regions.

Supplementary Table S15. *B. napus* Darmor-bzh large misassembled regions.

Supplementary Table S16. *B. napus* Darmor-bzh v5 RGA positions.

Supplementary Table S17. *B. napus* Darmor-bzh v10 RGA positions.

Supplementary Table S18. *B. napus* Darmor-bzh v5 v10 correspondance.

## Abbreviations

5mC, C5-methylcytosine; bp: base pairs; BUSCO: Benchmarking Universal Single-Copy Orthologs; Hi-C: high-throughput chromosome conformation capture; kb: kilobase pairs; LINE, long interspersed nuclear element; LMI: large misassembled inverted; LTR: long terminal repeat; Mb: megabase pairs; NBS: nucleotide binding site; NCBI: National Center for Biotechnology Information; NEB: New England Biolabs; ONT: Oxford Nanopore Technologies; PacBio, Pacific Biosciences; QTL: quantitative trait locus; RGA: resistance gene analogs; RT, reverse transcribed; TALC: Transcript-level Aware Long Read Correction.

## Data Availability

Darmor-bzh is a French winter variety, freely available in Plant Genetic Resources BrACySol (Rennes, France). All supporting data and materials are available in the *GigaScience* GigaDB database [59]. The Illumina and PromethION sequencing data and the Bionano optical maps are available in the European Nucleotide Archive under the projects PRJEB39416 and PRJEB39508. The genome assembly, gene predictions, and a genome browser are freely available from the Genoscope website [60].

## Competing Interests

J.M.A. received travel and accommodation expenses to speak at Oxford Nanopore Technologies conferences. J.M.A. and C.B. received accommodation expenses to speak at Bionano Genomics user meetings. The authors declare that they have no other competing interests.

## Funding

This work was supported by Genoscope, the Commissariat à l'Energie Atomique et aux Énergies Alternatives (CEA), and France Génomique (ANR-10-INBS-09–08). This project has received funding from the European Union's Horizon 2020 research and innovation programme under the Marie Sklodowska-Curie grant agreement No. 791 908 (J. Ferreira de-Carvalho post-doc salary), from the “‘Région Bretagne”’ and INRAE (Biology and Plant Breeding Department) for F. Boideau Ph.D. salary, and from the “Région Bretagne” for G. Richard post-doc salary (ref 0311/SAD18012/00 057661).

## Authors' Contributions

C.F., G.D., and C.C. extracted the DNA and the RNA. C.C. and A.L. optimized and performed the sequencing and the optical maps. C.F., R.D., and F.B. generated the different genetic maps for *Brassica napus*. B.I., C.B., and J.M.A. performed the genome assembly. C.F., J.M., J.F.d.C., F.B., M.R.G., C.B., and J.M.A. performed the anchoring of the *B. napus* scaffolds. S.E. performed the detection of modified bases. C.D.S. performed the error correction of direct RNA reads and the detection of alternative splicing events. J.M.A. and C.D.S. performed the gene prediction of the genome assembly. G.R., M.R.G., J.B., L.M., B.I., C.B., C.D.S., and J.M.A. performed the bioinformatic analyses. M.R.G., G.R., B.I., C.B., and J.M.A. wrote the manuscript. M.R.G., A.M.C., F.D., P.W., and J.M.A. supervised the study.

## Supplementary Material

giaa137_GIGA-D-20-00217_Original_Submission

giaa137_GIGA-D-20-00217_Revision_1

giaa137_GIGA-D-20-00217_Revision_2

giaa137_Response_to_Reviewer_Comments_Original_Submission

giaa137_Response_to_Reviewer_Comments_Revision_1

giaa137_Reviewer_1_Report_Original_Submission Huey Tyng Lee -- 8/24/2020 Reviewed

giaa137_Reviewer_2_Report_Original_Submission John Hamilton -- 8/24/2020 Reviewed

giaa137_Reviewer_2_Report_Revision_1 John Hamilton -- 10/4/2020 Reviewed

giaa137_Reviewer_3_Report_Original_Submission Valentine Murigneux, M.Sc. -- 9/1/2020 Reviewed

giaa137_Reviewer_3_Report_Revision_1 Valentine Murigneux, M.Sc. -- 9/26/2020 Reviewed

giaa137_Supplemental_Files
