# Supplementary material for: Long-read assembly of the Brassica napus reference genome Darmor-bzh
Source: Gigascience. 2020 Dec 15;9(12):giaa137. doi: 10.1093/gigascience/giaa137 (PMC7736779; doi:10.1093/gigascience/giaa137)
Supplement: giaa137_GIGA-D-20-00217_Revision_1 [file giaa137_giga-d-20-00217_revision_1.pdf]

GigaScience

Long-reads assembly of the Brassica napus reference genome, Darmor-bzh

--Manuscript Draft--

**Manuscript Number:**	GIGA-D-20-00217R1
**Full Title:**	Long-reads assembly of the Brassica napus reference genome, Darmor-bzh
**Article Type:**	Data Note
**Funding Information:**	Commissariat à l'Énergie Atomique et aux Énergies Alternatives — Not applicable<br>Agence Nationale de la Recherche (ANR-10-INBS-09-08) — Not applicable
**Abstract:**	Background: The combination of long-reads and long-range information to produce genome assemblies is now accepted as a common standard. This strategy not only allows access to the gene catalogue of a given species but also reveals the architecture and organisation of chromosomes, including complex regions like telomeres and centromeres. The Brassica genus is not exempt and many assemblies based on long reads are now available. The reference genome for Brassica napus, Darmor-bzh, which was published in 2014, has been produced using short-reads and its contiguity was extremely low if compared to current assemblies of the Brassica genus. Findings:Here, we report the new long-reads assembly of Darmor-bzh genome (Brassica napus) generated by combining long-reads sequencing data, optical and genetic maps. Using the PromethION device and six flowcells, we generated about 16M long-reads representing 93X coverage and more importantly 6X with reads longer than 100Kb. This ultralong-reads dataset allows us to generate one of the most contiguous and complete assembly of a Brassica genome to date (contigs N50 > 10Mb). In addition, we exploited all the advantages of the nanopore technology to detect modified bases and sequence transcriptomic data using direct RNA to annotate the genome and focus on resistance genes. Conclusion: Using these cutting edge technologies, and in particular by relying on all the advantages of the nanopore technology, we provide the most contiguous Brassica napus assembly, a resource that will be valuable for the Brassica community for crop improvement and will facilitate the rapid selection of agronomically important traits.<br>Keywords: oilseed rape, Brassica, Darmor-bzh, nanopore sequencing, optical mapping, assembly, chromosome-scale, direct RNA
**Corresponding Author:**	Jean-Marc Aury<br><br>FRANCE
**Corresponding Author Secondary Information:**	
**Corresponding Author's Institution:**	
**Corresponding Author's Secondary Institution:**	
**First Author:**	Mathieu Rousseau-Gueutin
**First Author Secondary Information:**	
**Order of Authors:**	Mathieu Rousseau-Gueutin<br>Caroline Belser<br>Corinne Da Silva<br>Gautier Richard<br>Benjamin Istace<br>Corinne Cruaud<br>Cyril Falentin<br>Franz Boideau<br>Julien Boutte

Powered by Editorial Manager® and ProduXion Manager® from Aries Systems Corporation

	Regine Delourme
	Gwenaëlle Deniot
	Stefan Engelen
	Julie Ferreira-de-Carvalho
	Arnaud Lemainque
	Loeiz Maillet
	Jérôme Morice
	Patrick Wincker
	France Denoeud
	Anne-Marie Chèvre
	Jean-Marc Aury
**Order of Authors Secondary Information:**	
**Response to Reviewers:**	First of all, we would like to thank the three reviewers for their meticulous reading of the manuscript. We have taken into account their comments, and believe that this new version of the manuscript is now of better quality. During the first submission, we put the article on the BioRxiv server and received some feedback from the scientific community, especially regarding the correspondence between genes of the v5 and v10 assemblies. We have decided to provide a correspondence table with this manuscript (Table S18) and have described our method and results in a dedicated section of the manuscript.<br>Reviewer #1:<br>The authors described a new Brassica napus genome assembly and compared it to other Brassica genomes, particularly including the older version of the same genotype. The comparisons in terms of gaps, anchored markers, anchored BUSCOs, gene annotation and RGA not only show solid evidence of improvement, but also provide the Brassica community with important information to transit from the older assembly version.<br><br>The manuscript is well-written with logical flow of methods and conclusions. I particularly enjoyed the emphasis on detailed explanation of technical methods, including choice of parameters and filtering criteria, because they enable readers to easily decide whether the results make sense. It also shows that the analyses were done with careful thoughts and thorough optimization.<br><br>Below are some minor comments, as well as suggestions that may or may not be addressed by the authors:<br><br>1.How well do the Illumina reads (ERX397788 and ERX397800) align to the Darmor v10, since they originated from data of a few years ago? This information is not crucial because 1) they were not directly used for gene prediction, and 2) TALC-corrected reads mapped better, but could be useful to judge the correctness of TALC outcome.<br>We aligned the Illumina reads on the v5 and v10 genome assemblies and observed a higher proportion of aligned reads on the v10 genome: 89.15% vs 88.42% for ERX397788 and 89.62% vs 88.51% for ERX397800. The difference is slight but in favor of the v10 assembly.<br><br>2.Line "These misassembled regions were validated using the new ZS11 assembly as a control (Figure 2, highlighted in purple on the C07 chromosome)": it looks like green instead?<br>We replaced purple with green in the text.<br><br>3.Figure 2: positions where pericentromeric-flanking markers aligned could be indicated, to further highlight the additionally assembled/inversed regions.<br>We have added in Figure 2 the alignment positions of pericentromeric-flanking markers.<br><br>4.Are alternatively spliced transcripts provided? File "BnapusDarmor-

Powered by Editorial Manager® and ProduXion Manager® from Aries Systems Corporation

bzh_annotation.gff" only contains primary transcripts.
The alternative transcripts were not predicted, at each loci a single transcript was annotated. However, we have now added requested files that contain alternative splicing events (intron retention and exon skipping) in the GigaDB repository.

5.Table S14 and S15: column names are missing
Column names of Tables S14 and S15 have been added.

6.Table S1: accession numbers are missing (probably pending, just a reminder)
Accession numbers have now been added to Table S1.

Reviewer #2:
In this Data Note, Rousseau-Gueutin et al. describe a new genome assembly and annotation of the allotetraploid Brassica napus Darmor-bzh. The assembly is based on Nanopore sequencing combined with an Bionano optical map. Previous assemblies are based on older technologies and issues with assembly and annotation quality make an upgraded reference genome and annotation a welcome and valuable resource for the Brassica research community.

I do have a number of comments, however, my copy of the manuscript does not have line numbers so I will refer to page numbers in the text.

Comments:
Pg 3 "Background" - the manuscript starts off with a well written background on the relationships of the Brassica genomes and a review of the genome sequencing of Brassica genomes, and status and issues of the B. napus Darmor-bzh v5 and v8 assemblies.
Thanks for your positive feedback.

Pg 5 "These trimming and removal steps were achieved using in-house-designed software based on the FastX package" - since the software is open source and in Github, just use its name (fastxtend) and parameters and version or commit id used for the analysis
We have changed the text accordingly and replaced “... were achieved using in-house-designed software based on the FastX package” with “... were achieved using Fastxtend tools”.

Pg 6 "A taxonomical assignation was performed using Centrifuge [16] for each dataset to detect potential contaminations." - needs more information (version, parameters, database) and report the results from the centrifuge run.
We added the centrifuge version, parameters and database information, as well as an overview of the result in the corresponding section : “On each run, approximately 65% of the reads were unassigned and almost 35% were assigned to the Brassica genus. In addition, we detected a small proportion (<0.6%) of reads corresponding to various metazoa.”

Pg 7 "In order to get the best possible genome assembly" -
I found this paragraph odd, especially in the context of a data note. There is no rationale for why these three assemblers were chosen other than "to get the best possible genome assembly" and of course, I could immediately think of another 6 assemblers you could have tried for achieve this goal. Flye is a fine choice, the results are excellent, so I would recommend modifying this paragraph and modifying the table to just report the assembly of the final contigs with Flye
We agree with the reviewer’s comment regarding the formulation “to get the best possible genome assembly”. We changed the sentence and replaced it with “We used three different assemblers…”. However, we have decided to keep the results of the three assemblers in supplementary data as we believe it may be useful for readers and especially for people working on genome assembly.

Pg7 "As nanopore reads contain systematic error" - I'm surprised that the assembly was not also polished with a tool that specializes in polishing nanopore assemblies such as Nanopolish or Medaka, especially as Nanopolish was used for the calling of 5mC bases from the raw WGS reads.
By the time we did the assembly years ago, nanopolish was too slow to work on large

genomes and medaka was at an early stage of development. That’s why we decided to use classic polishing tools like Racon and Pilon.

Pg 9 "Detection of modified bases" - This section showcases the calling from 5mCs from raw nanopore reads, however the results seem only to summarized as a track on Figure 1. This is fine, but the raw data for the track generated with what I assume is nanopolish call-methylation and the data filtered as described should be included in the data repository accompanying the paper
We added two files to the GigaDB repository. The first is the raw file, a tabular file with eleven columns that contain the nanopolish results for each nanopore read. The second is a tabular file with four columns used for generating the graph in Figure 1.

"We analysed the electric signal to detect the" - odd phrasing, rewrite to state the method used
We agree with the reviewer and decided to delete this sentence which was not informative.

Pg 10 - Gene Prediction - the gene annotation utilized a range of protein evidence along with the direct RNA reads aligned to the genome (using some venerable "old school" aligners!). Gene models were created using gmove, a tool I was not familiar with, but appears to be similar in function to EVM or MAKER to generate a consensus gene model. I'd like the authors to comment on why a de novo approach to annotation was not taken and why the direct RNA sequencing was not leveraged to predict isoforms.
Indeed, genewise and est2genome are “old school” aligners, but we agree that they are still very efficient in terms of alignment quality. We decided not to use a de novo approach, as the number of biological resources was high and specific genes could have been annotated using RNA-Seq from the same genotype. We did not predict the isoforms because we were concerned that the corrected nanopore reads might contain unreal isoforms. We believe that isoform prediction using long noisy reads is not mature enough, but this will undoubtedly be an important point to exploit while relying on future methods.

Pg 11 - "Brassica (CentBr1, CentBr2, CRB, PCRBr, TR238 and TR805) which were blasted" - Changed 'blasted' to 'searched'
We have modified the text accordingly.

Pg 12 - Comparison with existing assemblies and annotations
Throughout the manuscript, BUSCO results are not reported using the summary string recommended by the BUSCO developers. Also, the short_summary.*.txt BUSCO results file for the genome and annotation is missing from the data repo. As B. napus is an allotetraploid, I would like to see how many BUSCOs are complete and duplicated vs single.
This is a good point, we have added the full summary string in Table 1 for the three genomes of Darmor-bzh. However, since Table 3 is already dense, we prefered to create a new table in the supplementary Information for these additional results (Table S9). In addition, we have added all the BUSCO short_summary.*.txt in the GigaDB repository.

Pg 15 Alternative splicing events - The data describing the IR and skipped exon events in the results is not present in the data repo and should be added.
We have added requested files that contain alternative splicing events (intron retention and exon skipping) in the GigaDB repository.

General comment - Gigascience has set a high bar for reporting software tool versions and command line parameters to promote reproducibility. This information is missing throughout the manuscript and needs to be added. Data referred to in the results should also be added to the data repo accompanying the paper.
We have tried to be exhaustive, and we have added the version and parameters for each software we have used. In addition, the GigaDB repository now contains all the results generated for this publication.

Powered by Editorial Manager® and ProduXion Manager® from Aries Systems Corporation

Reviewer #3:
The authors present an improved version of the Darmor-bzh (Brassica napus) reference genome and annotation using a combination of long-reads sequencing data, optical and genetic maps. They report a detailed comparison of the new Darmor-bzh v10 genome assembly and annotation with previous releases of the same genome (evaluating contiguity, gene and repeat content, Resistance Genes Analogs) as well as with the other available Brassica assemblies. While they provide a valuable resource for the plant community, I have several major and minor comments.

Major comments
1) The assembly size of Darmor-bzh v10 reported in this paper (924 Mb) is larger than the previous releases v5 and v8 (850 Mb). Since the real size of the genome is unknown, it would be interesting to provide a genome size estimation based on k-mer analysis of Illumina reads or flow cytometry. The authors did not report the genome size parameter used to assemble the reads with Redbean and Flye. In Supplementary Table 1, they computed the coverage based on an estimated genome size of 1.2 Gb but it is unclear why they chose this value.
We chose the value of 1.2Gb because the genome size was first estimated at 1,132 Mb by Johnston et al. (https://doi.org/10.1093/aob/mci016) using flow cytometry. However, the k-mer spectrum (k=31) provides a lower estimate of 862Mb. We have added a paragraph that gives information on genome size estimation in the “Genome assembly” section.

2) Could you please provide more details about the criteria used to select the 'best' assembly? On page 7, the authors report that: 'Then, we selected the 'best' assembly based not only on contiguity metrics such as N50 but also cumulative size. The Flye assembler using the longest reads produced the most contiguous assembly and lead to a contig N50 of 10.0Mb.' My understanding is that the Flye assembly using the longest reads (ie only 30X instead of the 93X of data generated) was selected as the 'best' assembly because of its higher contig N50 and maybe also because of its cumulative size? (if yes, what criteria were used for the cumulative size?). Is that correct?
Yes you are right, the Flye (longest reads) and smartdenovo (all reads) assemblies were very similar in terms of contiguity (N50 and N90), but even though the N50 was slightly better using smartdenovo, we decided to keep the Flye assembly as its cumulative size was higher. We have now changed the text to explain our choice.

- The criteria to decide on a best assembly could include other metrics such as gene content and k-mer spectra. Have you estimated the BUSCO completeness for the different assemblers presented in Supplementary Table 2? A comparison of the k-mer spectrum of the assembly to the k-mer spectrum of the Illumina reads could be presented as well.
In general, the BUSCO score is a good metric for comparing assemblies, but in the case of unpolished assemblies the results are difficult to interpret. We added the BUSCO scores in Table S2, and the completeness scores were between 28.3% and 44.3% for wtdbg2, 54.2% and 90.9% for smartdenovo and 76.7% and 78.7% for Flye. In this case, the metrics reflect the accuracy of the polishing step of each assembler. For the same reason, the KAT plot is biased due to the consensus accuracy of each assembler. We have added the KAT plot for the raw and polished Flye assemblies in Figure S2.

- With Smartdenovo, adding more coverage resulted in a larger assembly with a higher contig N50. The authors reported in Supplementary Table 2 that the Flye assembly using all the reads "crashed". It would be interesting to see the Flye assembly results using 50 or 60X coverage, if the full dataset cannot be assembled. The Flye parameter --asm-coverage could be used to reduce the memory requirements of the program during the initial contig extension stage.
Indeed, the input dataset can be sampled in different ways, but generating numerous assemblies is time consuming. When we started the project we decided to generate three subsets of reads and use three different assemblers. Out of curiosity we ran the flye assembler with 50X of ONT reads, and got an assembly with a contig N50 of 9.4Mb (31 contigs), a contig N90 of 321Kb (209 contigs) and a cumulative size of 963Mb. So even though the cumulative size is higher, the N90 is twice as low as the selected assembly.

Powered by Editorial Manager® and ProduXion Manager® from Aries Systems Corporation

3) Long read sequencing technologies are a powerful tool to study alternative splicing and this is an area of active development. The 'Alternative splicing events' section could include additional analysis or discussion.
We first generated the direct RNA dataset to improve gene prediction and not to examine splicing events although this type of data opens up new perspectives in this area. Here we have pooled RNAs from two different tissues (leaf and root) and have not generated replicates which limits the applicative interest of this dataset.

- Have you tried to assess the confidence in the splicing events detected using the ONT long reads? It could be interested to try an alternative method or to include a comparison with the splicing events identified with the Illumina short reads.
To date, there is no mature software available that deals with ONT reads to detect splicing events. However, we have used the illumina dataset and run the same type of analysis to compare the intron retention detected by the two technologies. We find a good overlap between the two methods by allowing a low coverage of Illumina reads (>5). We have modified the corresponding section.

- The reported intron retention percentage in Darmor-Bzh v10 (16%) is much smaller than what was detected in Darmor-Bzh v5 (62%, Chalhoub et al., 2014). Intron retention detection is challenging but this could be investigated or discussed.
In Chalhoub et al., 62% refers to the proportion of IR events among the gene having splicing events (48%), showing that IR events are in the majority. The correct numbers are 29% and 16%, and the smaller difference can be explained by the lower sequencing depth of the nanopore dataset. We have added the points described above in the corresponding section.

- Could you provide the list of splicing events as a supplementary table?
We have added requested files that contain alternative splicing events (intron retention and exon skipping) in the GigaDB repository.

Minor comments
- It would be helpful to provide a schematic diagram summarising the genome assembly, scaffolding and annotation steps and including all the datasets and tools used to obtain the final v10 assembly.
We have added as a Supplementary Figure (Figure S3), a schematic diagram describing the different steps for obtaining the final assembly and the gene prediction.

- p3: The GBS abbreviation should be explained.
We have modified the text accordingly.

- p5-6: Nanopore sequencing sections. Which flow cell type was used?
For genomic and RNA sequencing, we used R9.4.1 flowcells. This information has been added to the nanopore sequencing sections.

- p6: Which basecalling tool was used ? Please also indicate the version and parameters used.
Genomic reads were basecalled using the guppy basecaller (version 1.4.3) with the ‘fast’ configuration and default parameters. RNA-Seq reads were basecalled using the guppy basecaller (version 3.2.8) with the ‘hac’ configuration and default parameters. We have added the missing information in the corresponding sections.

- p7: Long reads genome assembly section. Which version of each assembler and polishing tool were used?
We have added the assemblers’ version in the corresponding section.

- p7: Is there a reason for choosing to perform three iterations of Racon and three iterations of Pilon? For the selected assembly, it would be interesting to report the assembly metrics after each round of polishing including genome completeness estimation.
This is an interesting remark, we have followed the observation reported on the first plant genome assembled with nanopore data (https://dx.doi.org/10.1105%2Ftpc.17.00521). This study recommended performing three iterations of Racon, followed by three iterations of Pilon. We ran the BUSCO

Powered by Editorial Manager® and ProduXion Manager® from Aries Systems Corporation

analysis on each intermediate assembly to look at the gain of each iteration. We have now added these results to Table S3.

- p8: "As already reported, we found in several cases that the nanopore contigs were overlapping (based on the optical map)". Please include a citation of where this has been already reported.
We now cite the following article : https://doi.org/10.1038/s41477-018-0289-4.

- p8-10: Please also include the versions used for the tools mentioned ie nucmer, TALC etc.
We have added the software's versions in the corresponding section.

- p8-9: Large Misassembled Inverted regions section: Is the script to identify the LMI provided?
We have added the script (get_LMIs.sh) to the GigaDB repository.

- p13: "(Figure 2, highlighted in purple on the C07 chromosome)". I cant see any purple in Fig2 in the C07 chromosome plot, do you mean green?
We replaced purple with green in the text.

- p13: 'the proportion of anchored sequence (from 765 Mb for Express617 to 961 Mb for Zs11)'. The term proportion is mentioned but the reported numbers correspond to sizes.
We changed the text, and replaced 'the proportion of anchored bases' with 'the number of anchored bases'.

- p13: "The eleven Brassica genomes sequenced using PACBIO have a contig N50 between 1.4Mb and 3.6Mb whereas all the ONT assemblies have a contig N50 higher than 5.5Mb." I would replace "all the ONT" by "the four ONT" assemblies.
We replaced "all the ONT" with "the four ONT" assemblies.

- p14: A word is missing in the sentence "As a comparison, we found 5,460 in the PACBIO assembly of the Zs11 genotype."
We have added the missing word "gaps".

- p14, p16. Interestingly, the authors reported an example of a large transposable element resolved thanks to ONT ultra long reads (as compared to a gap in the PacBio+HiC assembly). They also assembled a filtered subset of reads ('Filtlong reads' including 2X >100 kb). Have you tried to assemble the ONT data without including the ultra long reads at all (ie 6X >100 kb)? It would be interesting to see the impact of those ultra-long reads on the overall assembly quality, especially because it could be challenging to generate ultra long reads and this is a specificity of the ONT technology.
As recommended we ran an assembly using Flye but without the ultra-long reads, and got an 894Mb assembly composed of 4,590 contigs, with a contig N50 of 9.3Mb and N90 of 349Kb. The N50 is still very high, but the assembly contains almost 3 times the number of contigs of the assembly obtained with ultra-long reads (4,590 vs 1,594). The assembly at the scaffold level will necessarily contain a higher number of gaps indicating the importance of long-reads.

- page 15 "From the genomic alignments of raw reads, with an identity percent higher than 90%". Which strategy was used to align the reads ?
Alignments were performed with Est2Genome, we have added the following description in the corresponding section : "Raw reads were aligned on the genome in a two-steps strategy. First, BLAT (version 36 with default parameters) was used to fastly localize corresponding putative regions of these RNA reads on the genome. The best match for each read was selected and a second alignment was performed using Est2Genome (version 5.2 with default parameters)."

Tables
- p26-27. Tables 1 and 2. I would add a column in those tables to include the reference paper and the corresponding release date for each assembly.
Table 1 and 2 are already dense, we preferred to create a new table that includes global information like the reference paper and the release date.

Powered by Editorial Manager® and ProduXion Manager® from Aries Systems Corporation

	Supplementary Tables<br>- Supplementary Table 1. The accession numbers are missing.<br>Accession numbers have now been added to Table S1.<br><br>- Supplementary Table 2:<br>-The authors reported that the Flye assembly using all the reads "crashed". Was it a memory issue due to the computing resources used?<br>No it was not a memory issue. In our experience, the earlier versions of Flye were not very stable and assemblies often crashed for no easily identifiable reasons. Flye is now much more stable and crashes are very rare thanks to the many updates that its authors have made.<br><br>-I would add the BUSCO completeness estimation for each of those assemblies.<br>Generally the BUSCO score is a great metric to compare assemblies, but in the case of unpolished assemblies the results are difficult to interpret. We added the BUSCO scores in Table S2, and the completeness scores were between 28.3% and 44.3% for wtdbg2, 54.2% and 90.9% for smartdenovo and 76.7% and 78.7% for Flye. In this case, the metrics reflect the accuracy of the polishing step of each assembler.<br><br>- Supplementary Table 8. Could you please add in the legend that 'Comp' refers to Comparative genomics (Long-range technology column)?<br>We added the following sentence in the legend : “ In the long-range technology column, “Comp” refers to comparative genomics meaning that contigs have been organized using synteny with an existing assembly at the chromosome-level .”
**Additional Information:**	
**Question**	**Response**
Are you submitting this manuscript to a special series or article collection?	No
**Experimental design and statistics**<br><br>Full details of the experimental design and statistical methods used should be given in the Methods section, as detailed in our Minimum Standards Reporting Checklist. Information essential to interpreting the data presented should be made available in the figure legends.<br><br>Have you included all the information requested in your manuscript?	Yes
**Resources**<br><br>A description of all resources used, including antibodies, cell lines, animals and software tools, with enough information to allow them to be uniquely identified, should be included in the Methods section. Authors are strongly encouraged to cite Research Resource Identifiers (RRIDs) for antibodies, model organisms and tools, where possible.	Yes

Have you included the information requested as detailed in our Minimum Standards Reporting Checklist?	
**Availability of data and materials**<br><br>All datasets and code on which the conclusions of the paper rely must be either included in your submission or deposited in publicly available repositories (where available and ethically appropriate), referencing such data using a unique identifier in the references and in the "Availability of Data and Materials" section of your manuscript.<br><br>Have you have met the above requirement as detailed in our Minimum Standards Reporting Checklist?	Yes

Manuscript

Click here to access/download;Manuscript;BrassicaNapus_genome_MainTe

Long-reads assembly of the *Brassica napus* reference genome, Darmor-bzh

Mathieu Rousseau-Gueutin[1,+,*], Caroline Belser[2,+], Corinne Da Silva[2,+], Gautier Richard[1,+], Benjamin Istace[2,+], Corinne Cruaud[3,+], Cyril Falentin[1,+], Franz Boideau[1], Julien Boutte[1], Regine Delourme[1], Gwenaëlle Deniot[1], Stefan Engelen[2], Julie Ferreira de Carvalho[1], Arnaud Lemainque[3], Loeiz Maillet[1], Jérôme Morice[1], Patrick Wincker[2], France Denoeud[2], Anne-Marie Chèvre[1], Jean-Marc Aury[2,*]

[+] Equal contribution

[*] Corresponding author

[1] IGEPP, INRAE, Institut Agro, Univ Rennes, 35653, Le Rheu, France

[2] Génomique Métabolique, Genoscope, Institut François Jacob, CEA, CNRS, Univ Evry, Université Paris-Saclay, 91057 Evry, France

[3] Genoscope, Institut François Jacob, Commissariat à l'Energie Atomique (CEA), Université Paris-Saclay, Evry, France

Abstract

Background:

The combination of long-reads and long-range information to produce genome assemblies is now accepted as a common standard. This strategy not only allows access to the gene catalogue of a given species but also reveals the architecture and organisation of chromosomes, including complex regions like telomeres and centromeres. The *Brassica* genus is not exempt and many assemblies based on long reads are now available. The reference genome for *Brassica napus*, Darmor-bzh, which was published in 2014, has been produced using short-reads and its contiguity was extremely low if compared to current assemblies of the *Brassica* genus.

Findings:

Here, we report the new long-reads assembly of Darmor-bzh genome (*Brassica napus*) generated by combining long-reads sequencing data, optical and genetic maps. Using the PromethION device and six flowcells, we generated about 16M long-reads representing 93X coverage and more importantly 6X with reads longer than 100Kb. This ultralong-reads dataset allows us to generate one of the most contiguous and complete assembly of a *Brassica* genome to date (contigs N50 > 10Mb). In addition, we exploited all the advantages of the nanopore technology to detect modified bases and sequence transcriptomic data using direct RNA to annotate the genome and focus on resistance genes.

Conclusion:

Using these cutting edge technologies, and in particular by relying on all the advantages of the nanopore technology, we provide the most contiguous *Brassica napus* assembly, a resource that will be valuable for the *Brassica* community for crop improvement and will facilitate the rapid selection of agronomically important traits.

Keywords: oilseed rape, *Brassica*, Darmor-bzh, nanopore sequencing, optical mapping, assembly, chromosome-scale, direct RNA

Background

Recently, Pacific Biosciences (PACBIO) and Oxford Nanopore (ONT) sequencing technologies were commercialized with the promise to sequence long DNA fragments (kilobases to megabases order). The introduction of these long-read technologies increased significantly the quality in terms of contiguity and completeness of genome assemblies [1–3]. Although it is possible to assemble chromosomes into a single contig in the case of simple genomes [4], the complexity of plant genomes is such that these methods are still insufficient to obtain the chromosome architecture. The resulting contigs need to be ordered and oriented with long-range information based on chromatin interactions or optical maps. This combined strategy is now a common standard.

Brassica crops include important vegetables for human nutrition and for production of vegetable oil. It is composed of several well studied species that belong to the U's triangle: three diploid species *B. rapa* (AA), *B. nigra* (BB) and *B. oleracea* (CC) and three allotetraploid hybrid species *B. juncea* (AABB), *B. napus* (AACC) and *B. carinata* (BBCC). As *Brassica* species underwent several polyploidy events, the genomes of the diploid and allopolyploid species are important models to understand the immediate and long term impact of polyploidy on the structural and the functional evolutionary dynamic of duplicated genes and genomes. These events had a significant role in their diversification, indeed the variability between two morphotypes of the same *Brassica* species can be high, underlining the importance of having a collection of high quality assemblies for the *Brassica* genus and more generally for all species.

To date, several genomes of the *Brassica* genus have been sequenced using long-reads and brought to the chromosome scale. The two first ones, *B. rapa* Z1 and *B. oleracea* HDEM, were published in 2018 and generated using ONT reads and optical maps [2,4]. At the beginning of 2020, two genomes of *B. rapa* (Chiifu) and *B. oleracea* (D134) have been released and sequenced using PACBIO long reads [5,6]. A first long-reads assembly based on ONT of the other diploid species, *B. nigra* NI100 is available since the beginning of 2020 [7]. The availability of the diploid genomes first may be explained by their smaller genome size (<600Mb) compared to their tetraploid derivatives. The first genome of a tetraploid plant has been published in 2014 [8] and was sequenced using a short-reads strategy (referred here as *B. napus* Darmor-bzh v5), and although being a great resource for the scientific community, this genome remains very fragmented and incomplete. In 2017, the pseudomolecules of Darmor-bzh were improved [9] using genotyping by sequencing (GBS) data (referred here as Darmor-bzh v8), and a new gene prediction was made. Unfortunately,

this annotation was not used by the *Brassica* community as it was considered incomplete [10]. As an illustration, only 89.6% of the core brassicale genes were found in the annotation compared to the 97.7% of the previous version (Table 1). In the first months of 2020, nine *B. napus* genomes based on PACBIO sequencing were published [11,12], and three of them were organized using long-range information. These genomes have a better continuity than the current reference (Darmor-bzh), but interestingly their N50 at the contig level is lower than the *Brassica* genomes that have been generated using ONT sequencing (Tables 2 and 3). Here, we report the genome sequence of the *B. napus* reference Darmor-bzh (referred here as Darmor-bzh v10), produced using ONT and Illumina reads supplemented by optical and genetic maps. In addition to chromosomes architecture, genes were predicted by sequencing native RNA molecules using the ONT PromethION with a focus on resistance genes. The contiguity and gene completion of this new genome assembly is among the highest in the *Brassica* genus (Tables 3 and S9).

DNA extraction and sequencing

Plant material

B. napus cv. Darmor-bzh is a French winter variety, freely available in Plant Genetic Resources BrACySol (Rennes, France). Darmor-bzh seeds were sown in Fertiss blocks (Fertil, France). Plantlets were grown under 16h light/8h night in a greenhouse at 20°C for 10 to 12 days of culture. Prior to harvest, plants were either dark treated during 5 days for DNA extraction or not treated after the 10 to 12 days of culture for RNA extraction.

DNA and RNA extractions

B. napus Darmor-bzh DNA extractions for nanopore sequencing were prepared from 1cm^2 of first young leaves from which the midribs were removed. Samples were placed on an aluminium foil on ice. 2.5g of each genotype were N_2 ground with mortar and pestle during 1 minute. Ground materials were homogenized with a 10ml pre-heated CF lysis buffer (MACHEREY-NAGEL GmbH & Co. KG) supplemented with 4mg proteinase K in 50ml tubes containing phase-lock gel and incubated for 45min at 56°C. 10ml of saturated phenol (25:24:1) was added to samples and tubes were placed on a rotator at 40 rpm for 10min to get a fine emulsion. Samples were centrifuged for 24min at 4500g (Acc3/Dec3) and aqueous phases were poured in a new 50ml tube containing phase-lock gel. 10ml of chloroform-octanol (24:1) were added to each sample and tubes were placed on a rotator at 40 rpm for 10min to get a fine emulsion. Samples were centrifuged for 24min at 4500g

(Acc3/Dec3) and aqueous phases were poured in a new 50ml tube. DNA precipitation was done by adding 4ml of NaCl 5M and 30ml of cold 100% isopropanol. After 3 hours at 4°C, DNA was hooked out in one-piece with a hook by melting a glass capillary in a blue flame. DNA pellets were submerged in a 50 ml tube containing 70% ethanol and transferred in a new tube to evaporate remaining isopropanol at 37°C (oven). Dried DNA was resuspended with a 3ml TE 10/1 buffer. Quality of extraction was evaluated using a 2200 TapeStation System (Agilent Technologies) with a Genomic DNA kit.

Total RNA was extracted from 2.5g *B. napus* leaf and root tissues using LiCl modified protocol [13]. RNA was controlled using a Bioanalyzer Chip (Agilent RNA 6000 Pico Kit) and quantified using Qubit (RNA HS Assay Kit).

Illumina Sequencing

DNA (1.5µg) was sonicated to a 100- to 1500-bp size range using a Covaris E220 sonicator (Covaris, Woburn, MA, USA). Fragments (1µg) were end-repaired, 3′-adenylated and Illumina adapters were then added using the Kapa Hyper Prep Kit (KapaBiosystems, Wilmington, MA, USA). Ligation products were purified with AMPure XP beads (Beckman Coulter Genomics, Danvers, MA, USA). Libraries were then quantified by qPCR using the KAPA Library Quantification Kit for Illumina Libraries (KapaBiosystems), and library profiles were assessed using a DNA High Sensitivity LabChip kit on an Agilent Bioanalyzer (Agilent Technologies, Santa Clara, CA, USA). Libraries were sequenced on an Illumina HiSeq2500 instrument (Illumina, San Diego, CA, USA) using 250 base-length read chemistry in a paired-end mode.

After the Illumina sequencing, an in-house quality control process was applied to the reads that passed the Illumina quality filters as described in [14]. These trimming and removal steps were achieved using Fastxtend tools [15]. This processing resulted in high-quality data and improvement of the subsequent analyses (Table S1).

Nanopore Sequencing (PromethION)

The libraries were prepared according to the protocol Genomic DNA by ligation (SQK-LSK108 and SQK-LSK109 Kit) – PromethION’ provided by Oxford Nanopore Technologies. Genomic DNA was first repaired and End-prepped with the NEBNext FFPE Repair Mix (New England Biolabs, Ipswich, MA, USA) and the NEBNext® Ultra™ II End Repair/dA-Tailing Module (NEB). DNA was then purified with AMPure XP beads (Beckman Coulter, Brea, CA, USA) and sequencing adapters provided by Oxford Nanopore Technologies were ligated using Concentrated T4 DNA Ligase 2M U/ml (NEB). After

purification with AMPure XP beads (Beckman Coulter) using Dilution Buffer (ONT) and Wash Buffer (ONT), the library was mixed with the Sequencing Buffer (ONT) and the Library Loading Bead (ONT), and loaded on the PromethION Flow Cells (R9.4.1). Output reads were basecalled using the Guppy basecaller (version 1.4.3) with the 'fast' configuration and default parameters.

No data cleaning was performed, the nanopore long reads were used raw for all the assemblies (Table S1). A taxonomical assignation was performed using Centrifuge (version 1.0.3, nr database from August 2018, and the following parameters --mm -p 5 -x nt_Aug2018 -q -k 15 --min-hitlen 26 --host-taxids 3708 --exclude-taxids 32644,28384,12908,77133) [16] for each dataset to detect potential contaminations. On each run, approximately 65% of the reads were unassigned and almost 35% were assigned to the *Brassica* genus. In addition, we detected a small proportion (<0.6%) of reads corresponding to various metazoa.

Direct RNA sequencing (PromethION)

rRNA was depleted using the Ribo-Zero rRNA Removal Kit (Plant Leaf). RNA libraries were performed from a mix of 960 ng mRNA according to the ONT protocol "Direct RNA sequencing" (SQK-RNA002). We performed the optional reverse transcription step to improve throughput, but cDNA strand was not sequenced. The nanopore library was sequenced using the PromethION on a single R9.4.1 flowcell and basecalled using the Guppy basecaller (version 3.2.8) with the 'hac' configuration and default parameters.(Table S11).

Optical Maps

B. napus Darmor-bzh DNA extractions for nanopore sequencing were prepared from 1cm^2 of first young leaves from which the midribs were removed. Samples were placed on an aluminium foil on ice. 5g of each genotype were N2 ground with mortar and pestle during 2 minutes. Ground materials were homogenized in 50ml NIBTM (10mM Tris-HCl pH 8.0, 10mM EDTA pH 8.0, 80mM KCl, 0.5M Sucrose, 1mM Spermine tetrahydrochloride, 1mM spermidine trihydrochloride, 2% (w/v) PVP40, pH was adjusted to 9.4 and solution was filtered through 0.22µm (NIB) and supplemented with 0.5% TritonX-100 (NIBT) and 7.5% 2-Mercaptoethanol (NIBTM). Nuclei suspensions were filtered through CheeseCloth and Mira Cloth and centrifuged at 1500g during 20min at 4°C. Pellets were suspended in 1ml NIBTM and adjusted to 20ml with NIBTM. Nuclei suspensions were filtered again through CheeseCloth and Mira Cloth and centrifuged at 57g during 2min at 4°C. Supernatants were

kept and centrifuged at 1500g during 20min at 4°C. Pellets were suspended in 1ml NIBT and adjusted to 20ml with NIBT. In order to wash the pellets, the last steps were repeated 3 times with 50ml of NIBT and a final time with 50ml of NIB. Pellets were suspended in residual NIB (approximately 200µl), transferred in a 1.5ml tube and centrifuged at 1500g during 2min at 4°C. Nuclei were suspended in cell suspension buffer from CHEF Genomic DNA Plug Kits (Bio-Rad) and melted agarose 2% from the same kit was added to reach a 0.75% agarose plug concentration. Plug lysis and DNA retrieval were performed as recommended by Bionano Genomics.

The NLRS labeling (BspQI) protocol was performed according to Bionano with 600ng of DNA. The DLS labeling (DLE-1), was performed with 750ng of DNA. Loading of the chip was performed as recommended by Bionano Genomics.

B. napus Darmor-bzh genetic map

An integrated *B. napus* genetic map was obtained using BioMercator V4.2 program [17] from three different genetic maps [18] : a cross between Darmor-bzh and Yudal (DH population with 248 lines), a cross between Darmor and Bristol (F2 population with 291 plants) and a cross between Darmor and Samouraï (DH population with 129 lines). These populations were genotyped using the Illumina 8K, 20K and 60K arrays [8,18–20] and genetic maps were constructed using CarthaGene software [21].

Genome assembly

Genome size estimation

The *Brassica napus* genome size was estimated using flow cytometry in a publication from Johnston et al. [22] that reported an haploid genome size of 1,132Mb. In addition, we launched Genomescope version 1 using Illumina reads and a kmer value of 31, and obtained an estimated genome size of 862Mb (Figure S1) and a very low heterozygosity rate (0.0526%). The results are highly discordant, however the genotype used for the flow cytometry experiment is not known. In addition, we observed a high divergence in terms of assembly size between the ten *B. napus* assemblies (Table 3) and a significant variation in genome sizes among different accessions of *B. rapa* has been recently described [23].

Long reads genome assembly

We used three different assemblers: Redbean v2.3 (git commit 3d51d7e) [24], SMARTdenovo (git commit 5cc1356) [25] and Flye v2.4.2 (git commit 5c12b69) [26] with all nanopore reads or subsets of reads composed of either the longest reads or those selected by the Filtlong [27] software (v0.2.0, git commit cf65a48) with default parameters (Table S2), as it has been proven that it could be beneficial for the assembly phase to downsample the reads coverage [3]. We used the following options as input to SMARTdenovo : “-c 1” to generate a consensus sequence, “-J 5000” to remove sequences smaller than 5kb and “-k 17” to use 17-mers as it is advised by developers in the case of large genomes.

Then, we selected the one of the assemblies based not only on contiguity metrics such as N50 but also cumulative size. The Flye (longest reads) and SMARTdenovo (all reads) assemblies were very similar in terms of contiguity (N50 and N90), but we decided to keep the Flye assembly as its cumulative size was higher. The Flye assembler using the longest reads resulted in a contig N50 of 10.0Mb and a cumulative size of 937.9Mb.

As nanopore reads contain systematic error in homopolymeric regions, we polished the consensus of the selected assembly three times with nanopore reads as input to the Racon [28] software (v1.3.2, git commit 5e2ecb7) and then three additional times using Illumina reads as input to the Pilon tool (v1.2.3, git commit be801ec) [29], as recommended in a previous study [3]. Both tools were used with default parameters (Table S3, Figures S2 and S3).

Long range genome assembly

The genome map assemblies of *B. napus* Darmor-bzh have been generated using the Bionano Solve Pipeline version 3.3 and the Bionano Access version 1.3 (Tables S4-S6). The assemblies were performed using the parameters “non haplotype without extend and split” and “add Pre-assembly”. This parameter allows to obtain a rough assembly used as reference for a second assembly. We filtered out molecules smaller than 180Kb and molecules with less than nine labeling sites (Tables S4 and S5). The Bionano scaffolding workflow was launched with the nanopore contigs and the two Bionano maps (DLE and BspQI). As already reported[2], we found in several cases that the nanopore contigs were overlapping (based on the optical map) and this overlap was not managed by the hybrid scaffolding procedure. We corrected these negative gaps using the BiSCoT software [30] with default parameters (Table S7 and Figure S3).

Validation and anchoring of the *B. napus* Darmor-bzh assembly

A total of 39,495 markers deriving from the Illumina 8K, 20K and 60K arrays were genetically mapped on the integrated map, totalling 2,777.7 cM. The sequence contexts of all SNP markers that were genetically mapped were blasted against our *B. napus* Darmor-bzh assembly in order to validate the quality of our assembly and to help ordering and orientating the scaffolds. Of these 39,495 genetically mapped markers, 36,391 were physically anchored on the final Darmor-bzh assembly (Figure 1 and Table S8). The genetic and physical positions were discordant for only 618 markers (0.02%) due to an inaccurate position on the genetic map (variation of a few centimorgans in almost all cases).

Identification of large misassembled inverted regions

In order to compare this new *B. napus* Darmor-bzh assembly (v10) to the first published version (v5) as well as to the recent *B. napus* ZS11 genome assembly [11], we performed whole chromosome alignments using nucmer from Mummer (version 3.23) [31], followed by automatic plotting using ggplot (version 3.3.0) in R (version 4.0.0) [32]. Nucmer was used with default parameters, followed by delta-filter with the `-i 98 -l 1000` parameters. Using the output of nucmer and delta-filter described above, we subsequently identified Large Misassembled Inverted (LMI) regions between the v5 and v10 genome assemblies. We used show-coords from Mummer (version 3.23) with the following parameters `-H -d -r -T`, followed by a specific method to extract LMIs. Since the show-coords output consists in many small alignments from which we need to infer LMIs, we used an iterative process based on merging the coordinates of small consecutive inversions in larger ones, followed by filtering out the small inversions. The parameters were validated by visually and manually checking the resulting LMIs coordinates against the chromosome by chromosome alignment scatter plots (Figure 2). More precisely, we extracted LMIs from the show-coords output, followed by `bedtools merge -d 100000` using bedtools (version 2.29.2) [33] to merge the coordinates of inversions separated by 100Kb or less, then we selected inversions of size superior to 100Kb, subsequently merged the inversions separated by 300Kb with bedtools, selected inversions larger than 700Kb, and finally merged inversions separated by 1Mb with bedtools.

Genome annotation

Detection of modified bases

The nanopore technology has the advantage of reading native DNA molecules and can distinguish modified from natural bases because they affect the electrical current flowing through the pore differently [34]. The nanopore reads were mapped using minimap2 [35] (version 2.17-r941 with the '-x map-ont' parameter). We called the 5-methylcytosine (5mC) in the CpG context of the whole genome of *B. napus* Darmor-bzh and computed frequencies using nanopolish version 0.12.5 with a minimum log-likelihood threshold of 2. CpG positions showing a frequency above 50% and covered by at least 4 reads were considered methylated. The proportion of methylated CpG per 100Kb across the genome is shown in Figure 1.

Correction of direct RNA reads

Direct RNA reads were corrected using TALC (version 1.0) [36] with default parameters (except -k=21) and Illumina reads from leaves and roots samples of *B. napus* downloaded from the EBI database (ERX397788 and ERX397800). Before the correction step, raw reads were masked using DustMasker (version 1.0.0 from the blast 2.10.0 package) [37], and we kept reads with at least 150 unmasked positions and at least 75% of unmasked bases. From 10,416,515 input reads, we retained 9,099,437 nanopore reads and TALC successfully corrected 8,523,238 reads (93.7%) with an average length of 645bp (Table S11).

Transposable elements annotation

Transposable elements (TE) were annotated using RepeatMasker (version 4.1.0 with default parameters) [38] and transposable element libraries from [8]. We masked nearly 54% of the genome and LTR Copia and Gypsy elements are the most abundant (15.5% and 13.0% respectively). The three Darmor-bzh assembly releases and the fourteen other *Brassica* genome assemblies were masked using the same procedure (Table 5).

Gene prediction

Gene prediction was performed using several proteomes: eight from other genotype of *B. napus* [11] (Westar, Zs11, QuintaA, Zheyou73, N02127, GanganF73, Tapidor3 and Shengli3), *Arabidopsis thaliana* (UP000006548), the *B. napus* pan-annotation [39], resistance gene analogs (RGA) from *Brassica* [40] and the 2014 annotation of Darmor-bzh

[8]. Low complexity in genomic sequences were masked with the DustMasker algorithms (version 1.0.0 from the blast 2.10.0 package) [37]. Proteomes were then aligned on the genome in a two-steps strategy. First, BLAT [41] (version 36 with default parameter) was used to fastly localize corresponding putative regions of these proteins on the genome. The best match and the matches with a score greater than or equal to 90% of the best match score have been retained. Second, alignments were refined using Genewise [42] (version 2.2.0 default parameters), which is more accurate for detecting intron/exon boundaries. Alignments were kept if more than 75% of the length of the protein is aligned on the genome. In addition, we used the error-corrected direct RNA reads produced using the PromethION device. As already reported [43], the detection of splicing sites using raw reads is difficult. For example, on a subset of 1,000 reads, the proportion of GT-AG splicing sites is 6% lower in the raw reads compared to the corrected reads (Table S12). Error-corrected reads were aligned on the genome in a two-steps strategy. First, BLAT (version 36 with default parameters) was used to fastly localize corresponding putative regions of these RNA reads on the genome. The best match for each read was retained and a second alignment was performed using Est2Genome [44] (version 5.2 default parameters), to detect exons boundaries. From the 8,523,238 corrected reads, we retained 4,651,118 alignments that covered at least 80% of the read with at least 95% identity. To reduce the redundancy, we clustered the alignments on the genomic sequence using bedtools (version v2.29.2-17-ga9dc5335) [33] and kept for each cluster the alignment with the highest score. The 70,904 resulting alignments (average identity of 97.5% and 4.07 exons per alignment) were used as input for the gene prediction.

All the transcriptomic and proteins alignments were combined using Gmove [45] which is an easy-to-use predictor with no need of pre-calibration step. Briefly, putative exons and introns, extracted from alignments, were used to build a graph, where nodes and edges represent respectively exons and introns. From this graph, Gmove extracts all paths from the graph and searches open reading frames (ORFs) which are consistent with the protein evidence. Finally, we decided to exclude single-exon genes predicted by RNA-Seq only and composed of more than 80% of untranslated regions (UTRs). Following this pipeline, we predicted 108,190 genes with 4.55 exons per gene on average. Quality of the gene prediction was estimated using the single-copy orthologous gene analysis from BUSCO v4 [46] with Brassicales version 10 which contains 4,596 genes (Table 1 and Figure S3).

Correspondence between the genes of the v5 and the v10 assemblies

We calculated a correspondence table between the predicted genes on the v5 and the v10 assemblies. To that purpose, we aligned all the v5 proteins against all the v10 proteins using diamond (version 0.9.24 with the following parameters: “--more-sensitive -e 0.00001”). The best reciprocal hits were selected if they came from the same chromosome in both assemblies, and used as anchors. We then enriched the anchored genes using synteny and by filtering hits based on percent identity (>80%) and sequence coverage (>80% of the target or the query). Using this strategy (M1), we were able to find a correspondence for 73,560 of the 101,040 genes of the v5 assembly (72.8%). Due to the importance of such information for the Brassica community, we decided to use a second method (M2) to increase the number of genes with a correspondence. For this latter method, we first performed a reciprocal BlastP (blastp v2.9.0 with the following parameters “-evalue 1e-20”) between the v5 and v10 proteins, then only conserved the blast hits with a minimum percentage of identity of 95%, an alignment length of 50%, and if they were on the same chromosome (scaffolds accepted). Using this second method, we identified about the same number of genes with a correspondence (72,314 v5 proteins). When comparing the results from these two methods, we observed that 85% were similar to both methods (62,514), about 14% were found in only one method (11,032 and 9,800 specifics to the first and second method respectively) and 0.6% were discordant (450). In this paper, we decided to provide the most complete correspondence table that derives from both analyses (82,441 proteins after removal of the 450 discordant results). In this supplementary table (Table S18), we indicate which method(s) (M1 and/or M2) allowed us to identify the correspondence between v5 and v10 proteins. Any updates to this table will be available here: http://www.genoscope.cns.fr/plants.

Localization and annotation of pericentromeric regions

We retrieved the markers flanking each centromere of *B. napus* chromosomes [47] and aligned them against the different assembly versions of *B. napus* Darmor-bzh (and also Zs11) to determine the improvement of the highly repeated pericentromeric regions. The (peri)centromere localization was also searched using six (peri)centromeric-specific repeat sequences of *Brassica* (CentBr1, CentBr2, CRB, PCRBr, TR238 and TR805) and by BLASTn[48] (version 2.9.0, with e-value: 10-6) against the Darmor-bzh v10 genome sequence. The length and number of genes present in the different versions were compared.

Annotation of the Resistance Genes Analogs

We identified the Resistance Genes Analogs (RGA) in Darmor-bzh v5 and v10 gene annotations using RGAugury (commit 57d58f887ad8c70e5d4c619a3d5e207158822819) [49] and the following command: `RGAugury.pl -p {peptides.fa} -e 1e-5 -c 10 -pfx {output}`. The latest versions of all the databases and tools used by RGAugury were downloaded as of April 2020 following the installation instructions provided in the RGAugury online repository [50].

Briefly, this pipeline enables to identify different major RGA families (NBS encoding; RLP: membrane associated receptor like proteins; RLK: surface localized receptor like protein kinases: RLK). Based on the presence or absence of some domain structures (CC: coiled-coil, TIR: Toll/Interleukin 1 Receptor, NB-ARC: nucleotide binding site activity regulated cytoskeleton; LRR: leucine rich repeat), the NBS encoding proteins were subdivided into eight different categories [51]. Finally, all the putative RGA proteins identified in Darmor-bzh v5 were blasted against those identified in this new assembly in order to establish a correspondence between these two versions, but also to identify newly annotated RGA genes in Darmor-bzh v10.

An improved version of the Darmor-bzh genome assembly

Comparison with existing assemblies and annotations

The two published releases of Darmor-bzh [8,9] were generated using 454 and Illumina reads and have a low contiguity (Table 1). These fragmented assemblies were difficult to organize at the chromosome level, and as a result only 553Mb and 690Mb of sequences were respectively anchored on the 19 chromosomes. In comparison, the 19 chromosomes of the ONT assembly contain 849Mb. The gene completion (BUSCO score) of the first release and of this one is similar (97.7% and 98.6%), showing that long-read assemblies mainly impact the repetitive compartment of the genome. However, 98.8% of the genes are now placed on their respective chromosomes rather than on unplaced scaffolds, in comparison only 80% of the predicted genes were located on pseudomolecules in Darmor-bzh v5 (Figure 3B). These improvements will greatly help the *Brassica* community to identify the genes underlying agronomic traits of interest found using quantitative genetics.

We compared the short- and long-read assemblies and observed large newly assembled regions comprising centromeric sequences (Figure 2, highlighted in orange on chromosome

A06). By aligning the flanking markers of *B. napus* centromeres [47], we were able to identify the positions of the approximative pericentromeric regions in Darmor-bzh v5 and the present Darmor-bzh v10 assembly (Table S14 and Figure 1). Thereafter, we compared the lengths and gene contents of these regions between Darmor-bzh v5 and v10 (Figure 3A). On average, we observed an increase of sequence assembly in pericentromeric regions by 80-fold, with some extreme cases like the A06 pericentromere that is 1,180 times larger in Darmor-bzh v10 compared to Darmor-bzh v5. Concerning pericentromeric gene content, we show an average increase of 24.4-fold, with the A06 pericentromere showing 290 times more genes in Darmor-bzh v10 compared to Darmor-bzh v5. There were only two exceptions (C01 and C05), where we identified a decrease in pericentromere size and gene content in Darmor-bzh v10 compared to Darmor-bzh v5. However, these results may in fact be attributable to misassembled inverted regions in Darmor-bzh v5, which changed the position of the markers on which our detection of pericentromeric regions is based. Indeed, comparison of the different *B. napus* Darmor-bzh assemblies allowed to notably identify 27 large misassembled inverted regions (LMIs: superior to 700 Kb) in Darmor-bzh v5 that are now correctly assembled in the Darmor-bzh v10 assembly. These misassembled regions were validated using the new ZS11 assembly [11] as a control (Figure 2, highlighted in green on the C07 chromosome). The 27 detected LMIs totalize 3,494 genes and 45Mb in Darmor-bzh v10, with the largest located on chromosome C01, measuring 6Mb and encompassing 276 genes. We provide a list of the large misassembled inverted regions detected in Darmor-bzh v5 and corrected in Darmor-bzh v10, alongside with the corresponding genes in these regions (Table S15).

All these analyses highlight the improvements of the Darmor-bzh v10 genome assembly thanks to the use of ONT long-reads sequencing. Overall, this high-quality genome assembly of *B. napus* Darmor-bzh will be particularly useful to the *Brassica* community to decipher genes underlying agricultural traits of interest.

Comparison of available *Brassica* genome assemblies

We downloaded the 14 available *Brassica* long-read assemblies (and annotations) and computed usual metrics. For each assembly, contigs were generated by splitting sequences at each gap and the gene completion was evaluated on predicted genes using BUSCO [46] and the conserved genes from the Brassicales database (N=4,596 genes). Not surprisingly the gene content is high, between 97.9% and 98.8% for all the ten *B. napus* assemblies (Tables 3 and S9), and is lower for the diploid species (due to the presence of a

single-genome), except for *B. rapa* Chiifu that has a surprising number of genes (>60k). Likewise, the repetitive content of these ten genomes is similar, between 54% for Darmor-bzh and 60% for no2127. Concerning the diploid genomes and as already reported [52], *B. oleracea* (C) genome assemblies contain more repetitive elements than the *B. rapa* (A) and *B. nigra* (B) genome assemblies (Table 5).

The main observed differences are the contig length that affects the number of gaps in each assembly (from 268 gaps for Darmor-bzh to 5,460 gaps for Zs11) and the number of anchored bases (from 765 Mb for Express617 to 961 Mb for Zs11). We further investigated these two differences that we believe to be related to the technologies used.

We observed a significant difference in contiguity when comparing ONT and PACBIO assemblies. The eleven *Brassica* genomes sequenced using PACBIO have a contig N50 between 1.4Mb and 3.6Mb whereas the four ONT assemblies have a contig N50 higher than 5.5Mb. The ability of the nanopore technology to sequence large fragments of DNA appears to be an advantage for assembling complex genomes. In the case of Darmor-bzh, we were able to obtain more than 50,000 reads longer than 100Kb (representing 6X of coverage). This dataset allowed us to generate a contiguous assembly with only 268 gaps. As a comparison, we found 5,460 gaps in the PACBIO assembly of the Zs11 genotype. This difference is observed in all the A and C genomes and subgenomes that have been sequenced using long reads, and may be directly related to the longer length of ONT reads (Figure 4). Although the number of gaps is higher in the PACBIO assemblies, the total number of N's is lower at least for the assemblies that have been organized with Hi-C data. Indeed, the Hi-C pipelines generally order contigs and add a fixed gap size between two contigs. We investigated the 500bp gaps in the Zs11 assembly and aligned their flanking regions on the Darmor-bzh assembly using blat (version 36 with default parameters) [41]. We applied stringent criteria (alignment on the same chromosome, score > 4000, alignment of at least 1 Kb of each of the flanking regions and alignment covering less than 100Kb on the Darmor-bzh assembly) and we found a location for 367 of the 5,460 gaps of Zs11. These 367 regions covered more than 19Mb of the Darmor-bzh assembly with an average size of 52 Kb. Even if these regions could be different between the two genotypes, we examined their content in transposable elements and found a high proportion of bases annotated as repetitive elements (82.9%) and a different distribution of the classes of elements compared to the whole genome (Table S13). We observed a higher proportion of Copia elements, but especially LINE and Satellite elements. For example, we observed large LINE elements

(>50Kb) as shown in Figure 5, where ultralong nanopore reads covered this element and avoided a contig breakage in this area.

Interestingly, A chromosomes seem more difficult to assemble (Figure 4). Even if A genome is smaller than C genome and contains less transposable elements (Table 5), the number of contigs (and consequently the number of gaps) in A genome, or subgenome, is twice the number of contigs in C genome, or subgenome (gap track on Figure 1 and Figure 4). Chromosomes A05, A06 and A09 appear to be the most challenging to assemble due to the high content of transposable elements in their centromeric regions. They alone contain 43% of the gaps (and 51% of the undetermined bases) present on the 19 chromosomes. However, these chromosomes have been shown to be highly variable in length across *B. rapa* genotypes [23].

The second difference observed between all these assemblies is the proportion of anchored sequence which is higher for the genome assembly that has been organized using Hi-C data (95.0%, Zs11) although it is the most fragmented (Table S10). Indeed, the organization of small contigs is complicated using optical or genetic maps because it requires a sufficient number of restriction sites or markers. This aspect probably explains the small proportion of the Express617 assembly (82.7%) that has been anchored on the 19 chromosomes. The other genomes have been organized using comparative genomics (synteny with the Zs11 assembly). Although it is a convenient method, it may generate false organization and the proportion of anchored bases is variable, from 89.1% to 92.7%, depending on the conservation between the two genomes. We think that if the assembly is highly contiguous, optical maps have the important advantage of estimating the size of gaps, which remains a limitation when using Hi-C data.

Sequencing of native RNA molecules

The sequencing of RNA is traditionally performed using the Illumina technology where the RNA molecules are isolated and then reverse transcribed (RT) to cDNA that is more stable and allows the amplification prior to the sequencing. The Oxford Nanopore technology is the first to propose the sequencing of native RNA molecules without the RT step. One of the main advantages is a better quantification of gene expression when compared to methods that require a RT [43]. Moreover the sequencing of native RNA molecules preserves modified bases and theoretically allows to detect them [53,54]. Here, we sequenced a mix of leaf and root samples using the PromethION device and generated 10,416,515 reads with

an average size of 559 bp. This dataset was corrected using TALC as described previously and corrected reads were used in the gene prediction workflow (Table S11).

Alternative splicing events

Independently, we detected alternative splicing (AS) events, as skipping exon and intron retentions. Aligning noisy reads makes it difficult to accurately detect splice sites and therefore 5’ and 3’ alternative splice sites. Raw reads were aligned on the genome in a two-steps strategy. First, BLAT (version 36 with default parameters) [41] was used to fastly localize corresponding putative regions of these RNA reads on the genome. The best match for each read was selected and a second alignment was performed using Est2Genome [43] (version 5.2 with default parameters). Alignments with an identity percent higher than 90% were retained and we used bedtools to extract intron retention and skipping exon. We searched for predicted introns that were entirely covered by an exon of a nanopore read. Using this conservative approach, we were able to identify 30,397 events distributed across 18,204 genes (16.8%). In Chalhoub et al. [8], they detected an intron retention in 29% of the 108,190 annotated genes. Using the same approach and a coverage of at least 10 Illumina reads, we found intron retention in 21.2% of the genes. This proportion increased to 24.4% and 30.3% if lower coverage was used (8 and 5 respectively). We compared the intron retention predicted by Nanopore and Illumina reads and found only 56% in common and therefore 44% of the events are specific to Nanopore reads. However, using lower coverage for prediction with Illumina data increased the proportion of common events (62% with coverage >8 and 73% with coverage >5) indicating that Nanopore reads can also detect rare events. However, the smaller number of events detected using the Nanopore reads may indicate that sequencing 10M reads is not sufficient and there is a need to sequence more deeply the RNA sample. In addition, we detected skipping exon (reads with one exon that is entirely covered by an intron, smaller than 3Kb to avoid mapping errors) in 3% of the 108,190 annotated genes. For example, by inspecting mutually exclusive exons, we were able to find an already described event that is conserved between *Brassica* and *Arabidopsis thaliana* [55] (Figure 6). It is obvious that the sequencing of RNA using long-reads will allow to detect co-occurrence of splicing events that is difficult if not impossible to do with short-reads (Figure S4).

Comparison of the Resistance Genes Analogs catalogs

Using the RGAugury pipeline [47], we annotated 2,788 and 2,952 Resistance Genes Analogs (RGA) in the gene catalogs of Darmor-bzh v5 and v10 respectively (Tables S16 and

S17). In Darmor-bzh v5 only 83.5% of the RGAs were located on anchored scaffolds, while we identify 99.4% of the RGAs on chromosomes in Darmor-bzh v10. On average 153±46 RGAs are located on each pseudomolecule in Darmor-bzh v10 compared to 122±33 in Darmor-bzh v5 (rounded mean±standard deviation). Overall, more RGAs were annotated in the Darmor-bzh v10 annotations in almost all categories, thanks to the long-reads sequencing. Most of the RGAs found in Darmor-bzh v5 were conserved in Darmor-bzh v10 (81,4%, Table 4 and Figure 7A). However, we observed that 108 RGAs annotation shifted from one RGA class to another between the two versions due to the identification of additional domains in these proteins (Figure 7A). Between the Darmor-bzh v5 and Darmor-bzh v10 annotations, the CN and NL RGA classes shifted the most to CNL (CC, NB-ARC and LRR domains, Figure 7B and 7C), which are displaying one additional domain compared to CN (CC and NB-ARC domains) or NL (NB-ARC and LRR domains) RGAs. In addition, we identified dozens of examples where few adjacent RGA genes in the Darmor-bzh v5 now correspond to a single gene in the Darmor-bzh v10.

As pericentromeric region assembly was greatly improved in Darmor-bzh v10, we compared the number of RGA in such regions between the two assemblies and found 74 RGAs in Darmor-bzh v10 compared to no RGAs in Darmor-bzh v5 (Figure 7D). To further highlight the interest of assembling such regions using ONT technologies, we compared the size and number (including RGA) of a resistance QTL to phoma stem canker overlapping the A01 centromere [47,50]. In this new assembly, we were able to identify 45 candidate RGA compared to 29 in Darmor-bzh v5 (Figure 7E). This finding opens new ways to understand the mechanism underlying this QTL.

Conclusions

In this study, we generated the most contiguous *B. napus* genome assembly thanks to the ONT long reads and Bionano optical maps. In addition, the ONT dataset allows the detection of modified bases, such as C5-methylcytosine (5mC), without any specific library preparation. We have shown that the generation of ultra-long reads is a game-changer for assembling complex regions, composed of transposable elements, common in plant genomes. Consequently, the combination of ultralong reads and optical maps is today a method of choice for generating assembly of complex genomes. Without forgetting that nanopore technology is in constant evolution, in particular base calling softwares, which makes it possible to improve an assembly starting from existing data. In addition, we have

predicted genes in this new assembly using direct RNA sequencing and have used this original dataset to detect splicing events and show that this technology can be used to discover complex events, such as the co-occurence of events as mutually exclusive exons. This improved version of the *B. napus* Darmor-bzh reference genome and annotation will be valuable for the *Brassica* community, which has now been working with the short-read version for almost six years, particularly to decipher genes underlying agricultural traits of interest.

Additional files

All the supporting data are included in six additional files which contain a) Tables S1-S13 and Figures S1-S4, b) Table S14, c) Table S15, d) Table S16, e) Table S17 and f) Table S18.

Acknowledgements

This work was supported by the Genoscope, the Commissariat à l'Energie Atomique et aux Énergies Alternatives (CEA) and France Génomique (ANR-10-INBS-09-08). We acknowledge the BrACySol BRC (INRA Ploudaniel, France) that provided us with the seeds of *B. napus* cv. Darmor-bzh. The authors are grateful to Oxford Nanopore Technologies Ltd for providing early access to the PromethION device through the PEAP, and we thank the staff of Oxford Nanopore Technologies Ltd for technical help.

Availability of supporting data

Darmor-bzh is a French winter variety, freely available in Plant Genetic Resources BrACySol (Rennes, France). The Illumina and PromethION sequencing data and the Bionano optical maps are available in the European Nucleotide Archive under the following projects PRJEB39416 and PRJEB39508. The genome assembly, gene predictions and a genome browser are freely available at http://www.genoscope.cns.fr/plants.

Competing interests

The authors declare that they have no competing interests. JMA received travel and accommodation expenses to speak at Oxford Nanopore Technologies conferences. JMA and CB received accommodation expenses to speak at Bionano Genomics user meetings.

Funding

This work was supported by the Genoscope, the Commissariat à l'Energie Atomique et aux Énergies Alternatives (CEA) and France Génomique (ANR-10-INBS-09-08). This project has received funding from the European Union's Horizon 2020 research and innovation programme under the Marie Sklodowska-Curie grant agreement No 791908 (J. Ferreira-de-Carvalho post-doc salary), from the ''Région Bretagne'' and INRAE (Biology and Plant Breeding Department) for F. Boideau Ph.D salary, and from the "Région Bretagne" for G. Richard post-doc salary (ref 0311/SAD18012/00057661).

Author's contributions

CF, GD and CC extracted the DNA and the RNA. CC and AL optimized and performed the sequencing and the optical maps. CF, RD and FB generated the different genetic maps for *Brassica napus*. BI, CB and JMA performed the genome assembly. CF, JM, JFdC, FB, MRG, CB and JMA performed the anchoring of the *B. napus* scaffolds. SE performed the detection of modified bases. CDS performed the error correction of direct RNA reads and the detection of alternative splicing events. JMA and CDS performed the gene prediction of the genome assembly. GR, MRG, JB, LM, BI, CB, CDS and JMA performed the bioinformatic analyses. MRG, GR, BI, CB and JMA wrote the article. MRG, AMC, FD, PW and JMA supervised the study.

Figure 1. Genome overview of the 19 chromosomes of *B. napus* Darmor-bzh v10 (the ten A chromosomes are in blue and the nine C chromosomes in orange). A. Localization of centromere from flanking markers identified by [47], **B.** Density of repeated pericentromeric-specific sequences of *Brassica* allowing more precise localization of centromeres. **C.** Density of Gypsy elements. **D.** Density of Copia elements. **E.** Density of DNA transposon elements. **F.** black boxes represent gaps in the Darmor-bzh assembly. **G.** Density of methylated CpG. **H.** Gene density. **I.** scatter plots of RGAs density. All densities are calculated in 100Kb windows, blue and red colors in density plots are for lower and higher values respectively.

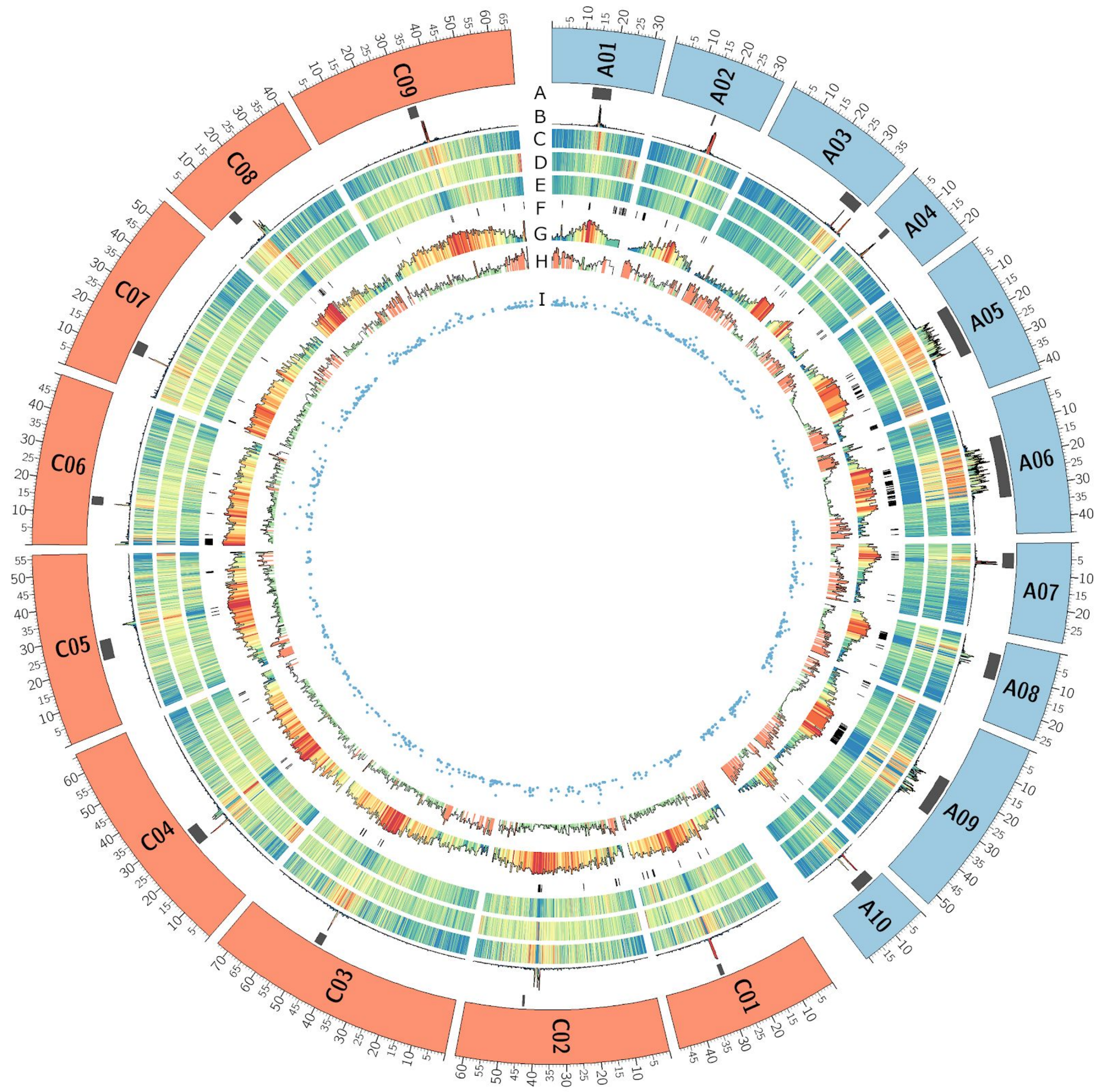

Figure 2. Genome-wide alignments of *B. napus* Darmor-bzh v10 (Y axis) with Darmor-bzh v5 and ZS11 genome assemblies (X axis). Each dot corresponds to syntenic regions of the genomes that are aligning with high confidence. Blue dots are corresponding to regions aligned in the correct orientation (+) while red dots represent regions aligned in an inverted orientation (-). (Peri)centromeric regions are displayed by black boxes on the X and Y axes. Some (peri)centromeres flanking markers were not found in Zs11 due to polymorphisms. The new assembly of a centromeric region in Darmor-bzh v10 compared to Darmor-bzh v5 is highlighted in orange for the A06 chromosome. An example of a large misassembled inverted region whose orientation has been corrected within the new Darmor-bzh v10 genome assembly is highlighted in green for the chromosome C07.

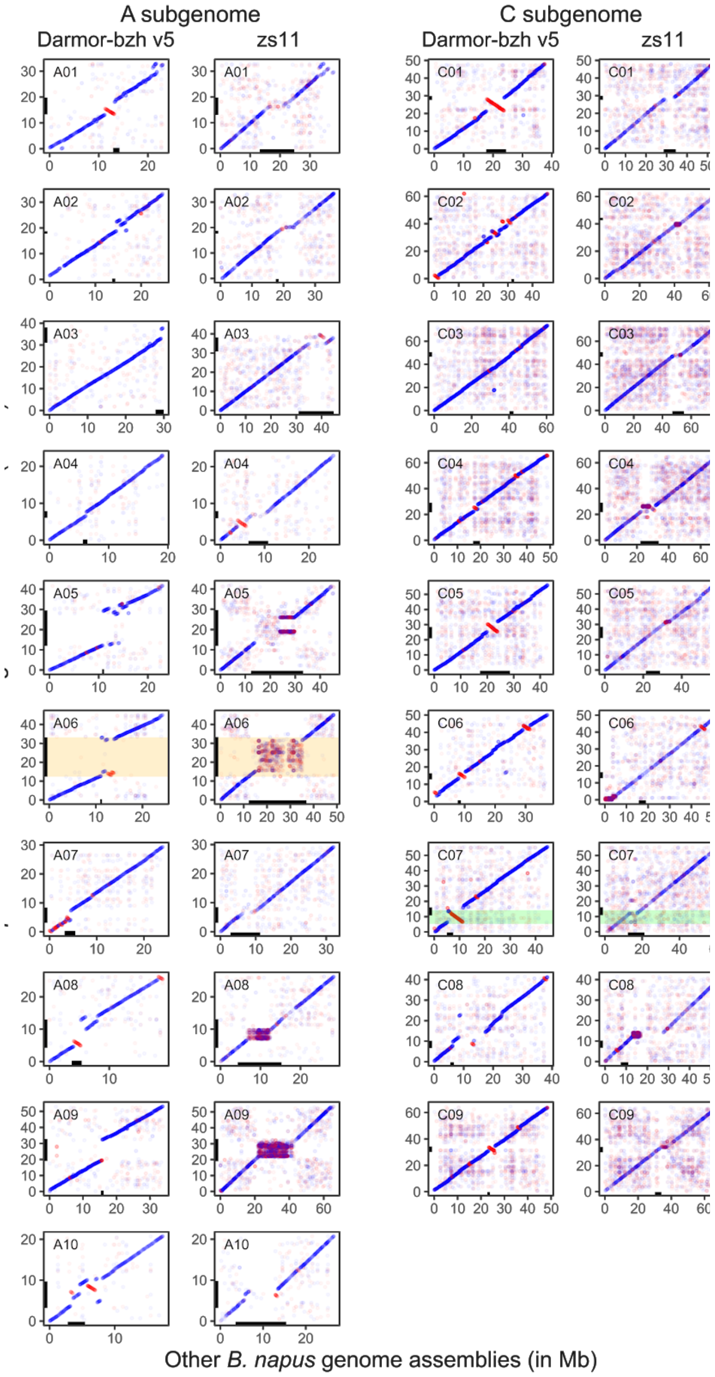

Figure 3. Improvements of the *B. napus* Darmor-bzh v10 genome assembly and annotation compared to Darmor-bzh v5 by the use of long reads sequencing. A. Genome-wide pericentromeres size (in Mb) and gene content using centromere flanking markers (Mason et al. 2016, refer to methods). The Darmor-bzh v10 genome assembly now harbours larger pericentromeres with more annotated genes than in the previous genome. **B.** Repartition of genes location on chromosome and unplaced scaffolds.

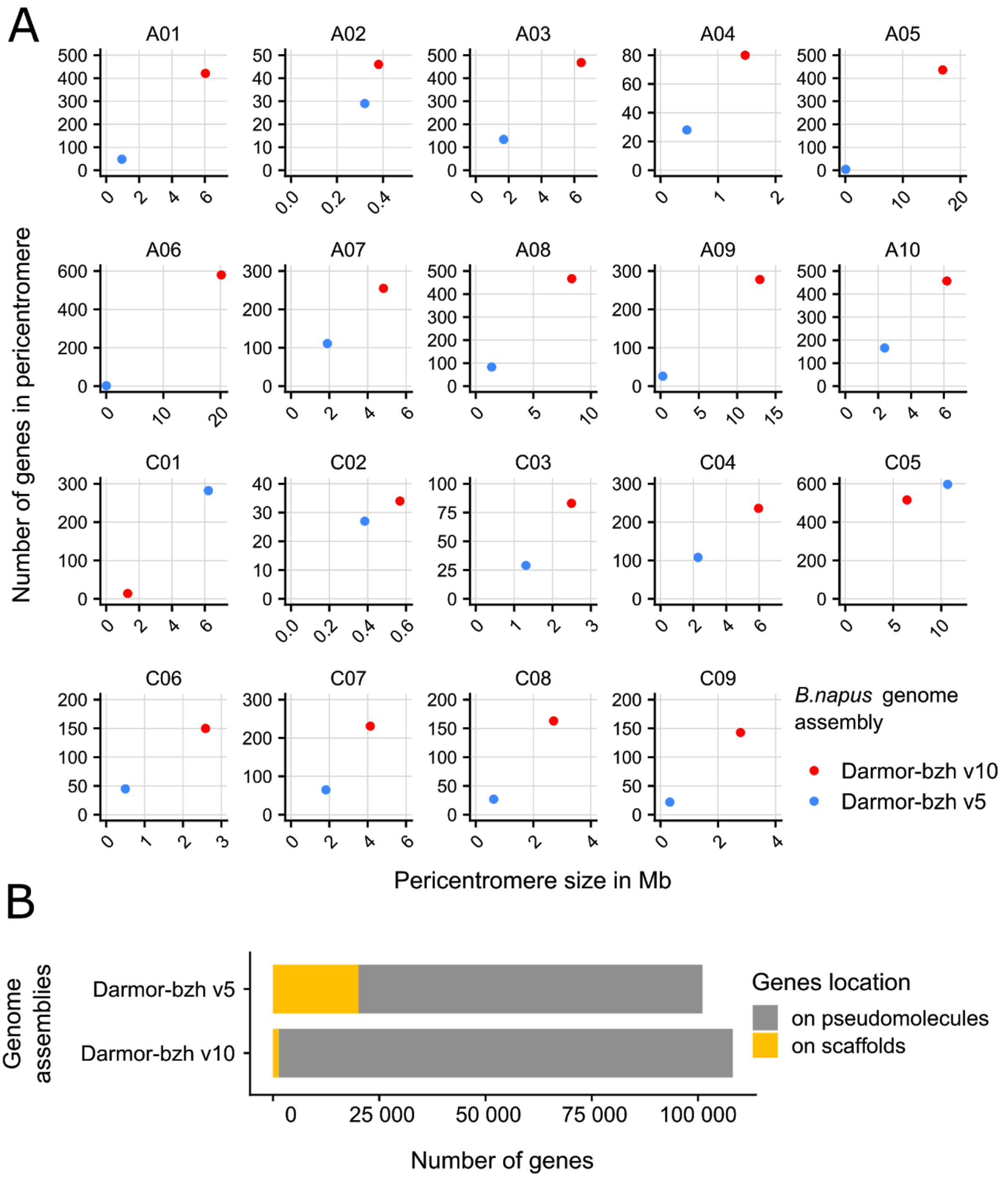

Figure 4. Distribution of the number of gaps per chromosome in *Brassica* genomes. Each dot represents the number of gaps in a given chromosome and genome assembly. PACBIO assemblies are in blue and ONT assemblies in orange.

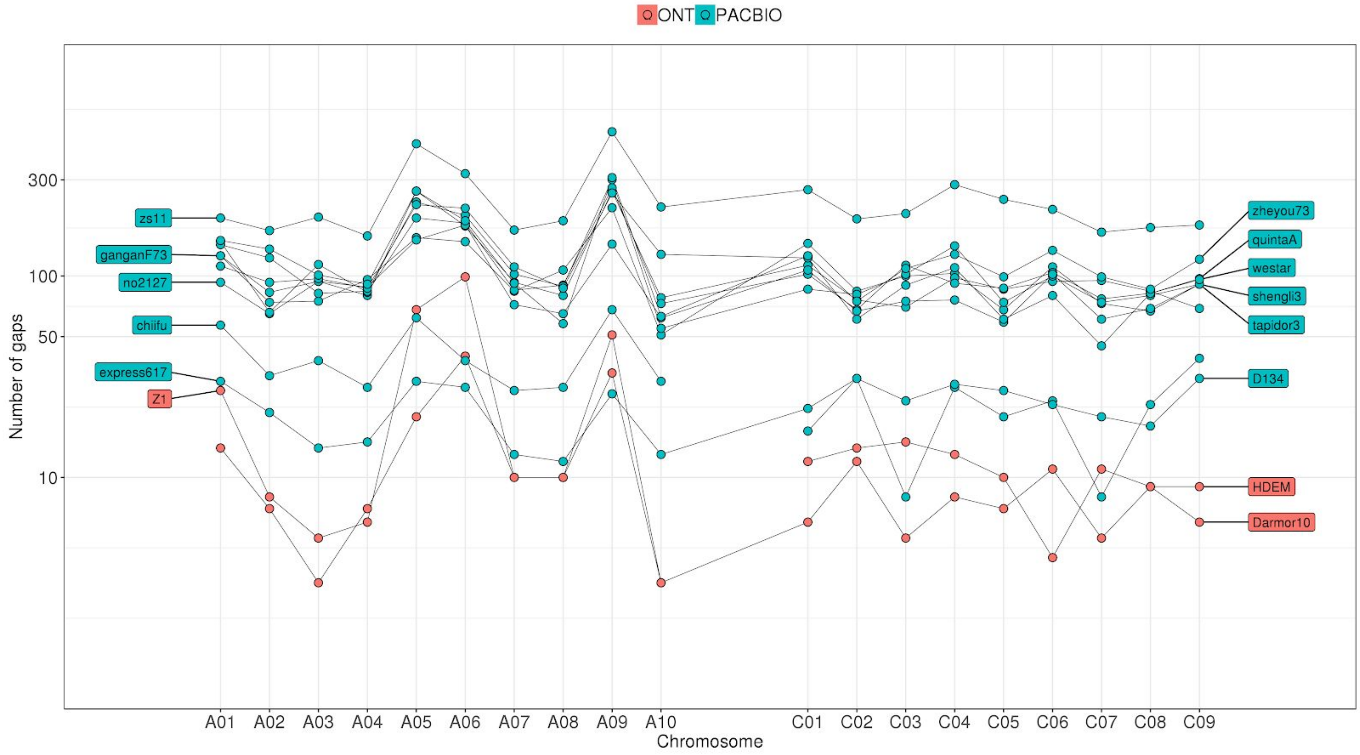

Figure 5. Example of a genomic region of *B. napus* Darmor-bzh assembly that corresponds to a region that contain a gap in the Zs11 genome assembly. First track represents the GC content. ONT reads (longer than 100Kb) are in the second track (blue boxes), and a 115 Kb read that spanned the whole region is surrounded by a black box. Transposable elements are shown as black boxes, and the region absent from Zs11 contain a large LINE element (80,707 bp). The alignment of the Zs11 sequence (20Kb around the 500bp gap) is represented by red boxes, with thin red lines representing missing sequences in the Zs11 genome assembly that are present in Darmor-bzh v10 (gaps). Predicted genes are in the last track, in purple.

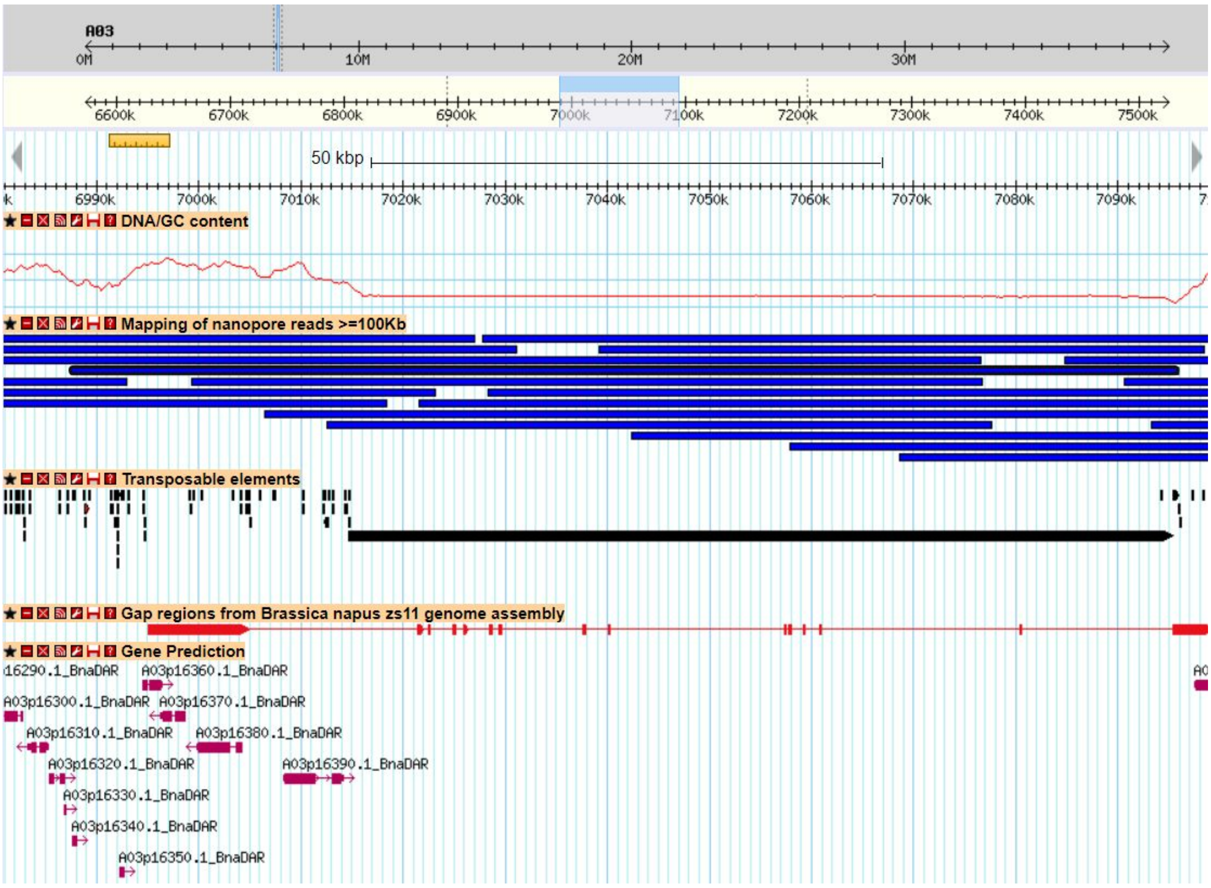

Figure 6. Example of a splicing event detected using long reads. The exon in the third introns of the gene prediction (C03p53740.1_Bna_DAR, Gene prediction track) is mutually exclusive with the third exon. This alternative splice form is detected by a single nanopore read (blue track), and maintained by the TALC error-correction (red track).

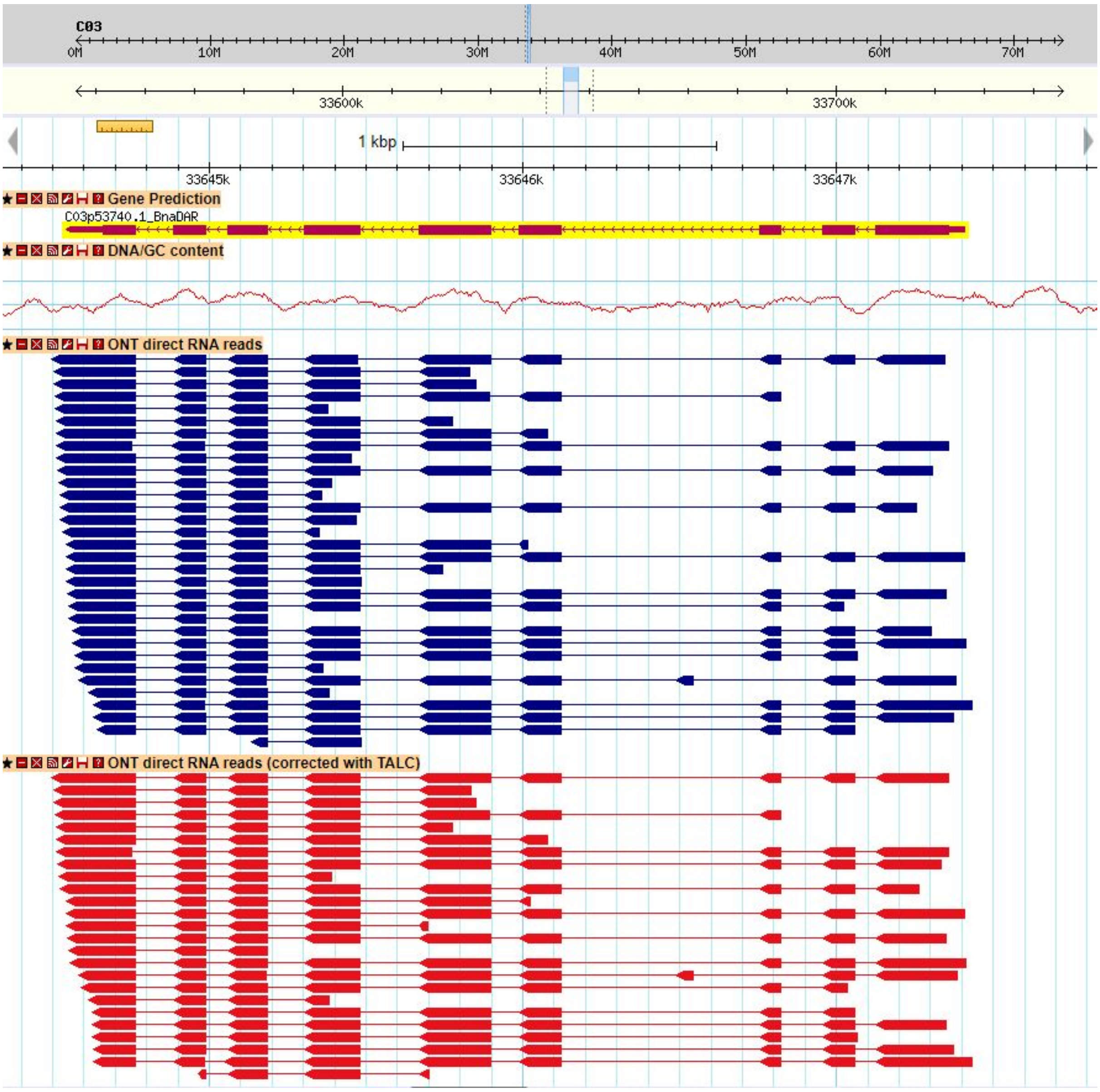

Figure 7. Resistance Genes Analogs (RGA) annotation improvement in *B. napus* Darmor-bzh v10 using long reads direct RNA sequencing. A. Conservation of the RGA categories between Darmor-bzh v5 and v10 genome annotations. Shifting RGAs are genes whose sequence is matching, but that have been annotated in a different RGA class by RGAugury between the v5 and v10 genome annotations. Such shifting RGAs only represent a small fraction of the annotated RGAs. **B.** Total number of RGA plotted against the ratio of shifting RGAs over total RGAs by RGA class. CN and NL RGAs are the categories whose annotation shifted the most between Darmor-bzh v5 and v10. A detail of the raw data used to compute the ratio is available on the right part of the plot. **C.** Detail of the RGA class shifts between Darmor-bzh v5 and v10 made using ggalluvial [56]. The size of the arcs is proportional to the number of shifting RGAs from one RGA class to another between the two annotations. **D.** Number of pericentromeric RGAs found in Darmor-bzh v10 compared to the v5 genome annotation. **E.** Genome browser snapshot using pyGenomeTracks [57] of the phoma canker resistance QTL (grey) in Darmor-bzh v5 (top) and Darmor-bzh v10 (bottom). RGA genes (blue) and pericentromeric regions (orange) are displayed.

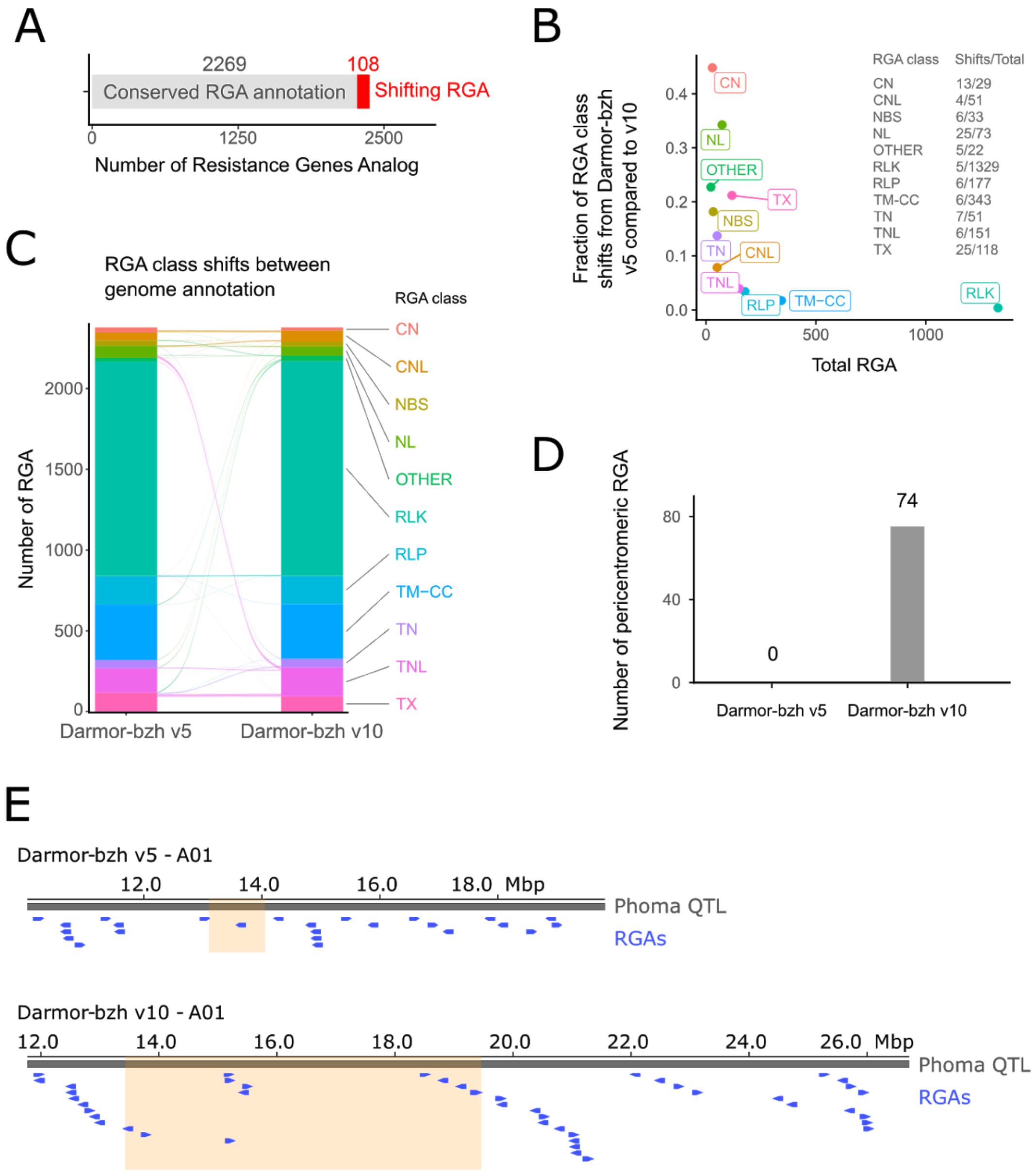

Tables

Table 1. Statistics of the Darmor-bzh assemblies.

	**version**	technology	contigs N50 (L50)	# contigs	Cumulative size	Number of genes	Number of anchored bases	Number of anchored [ACGT] bases	BUSCO scores n=4596
B. napus	10	ONT	11,486,274 (24)	505	924 Mb	108,190	867 Mb (93.84%)	849 Mb	C: 98.6% S: 7.0% D: 91.6% F: 0.1% M: 1.3%
B. napus	8	454	37,644 (5,517)	44,818	850 Mb	80,382	799 Mb (93.96%)	690 Mb	C: 89.6% S: 18.0% D: 71.6% F: 3.4% M: 7.0%
B. napus	5	454	37,644 (5,517)	44,837	850 Mb	101,040	645 Mb (75.90%)	553 Mb	C: 97.7% S: 9.9% D: 87.8% F: 0.5% M: 1.8%

Table 2. General information about the available *Brassica* long-reads and older Darmor-bzh assemblies.

	Subgenome	genotype	Principal sequencing technology	Release year	Reference
B. rapa	A	Z1	ONT	2018	Belser et al.[2]
B. rapa		Chiifu	PACBIO	2018	Zhang et al. [58]
B. nigra	B	NI100	ONT	2020	Perumal et al.[7]
B. oleracea	C	HDEM	ONT	2018	Belser et al.[2]
B. oleracea		D134	PACBIO	2020	Honghao et al.[5]
B. napus	AC	Darmor-bzh v5	454	2014	Chalhoub et al.[8]
B. napus		Darmor-bzh v8	454	2017	Bayer et al.[9,12]
B. napus		Darmor-bzh v10	ONT	2020	This study
B. napus		Westar	PACBIO	2020	Song et al.[11]
B. napus		Express617	PACBIO	2020	Lee et al.[12]
B. napus		Tapidor3	PACBIO	2020	Song et al.l.[11]
B. napus		Shengli3	PACBIO	2020	Song et al.l.[11]
B. napus		QuintaA	PACBIO	2020	Song et al.l.[11]
B. napus		No2127	PACBIO	2020	Song et al.l.[11]
B. napus		GanganF73	PACBIO	2020	Song et al.l.[11]
B. napus		Zheyou73	PACBIO	2020	Song et al.l.[11]
B. napus		Zs11	PACBIO	2020	Song et al.l.[11]

Table 3. Statistics of the *Brassica* long-reads assemblies (ordered by contigs N50 value, *B. napus* are in bold). Full BUSCO scores are available in Table S9.

	genotype	technology	contigs N50 (L50)	# contigs	Max contig size	# scaffolds	cumulative size	Number of genes	Complete BUSCO n=4596
B. nigra	NI100	ONT	11,504,526 (13)	187	32,581,047	58	506 Mb	59,851	97.2%
B. napus	**Darmor-bzh**	**ONT**	**11,486,274 (24)**	**505**	**46,624,681**	**237**	**924 Mb**	**108,190**	**98.6%**
B. oleracea	HDEM	ONT	9,491,203 (19)	264	26,712,175	129	555 Mb	61,279	96.2%
B. rapa	Z1	ONT	5,519,976 (17)	627	22,127,468	304	402 Mb	46,721	96.7%
B. oleracea	D134	PACBIO	3,591,417 (43)	229	15,061,548	9	530 Mb	43,868	95.1%
B. napus	**Westar**	**PACBIO**	**3,130,520 (93)**	**5,602**	**15,044,602**	**3,458**	**1,008 Mb**	**100,194**	**98.3%**
B. napus	**Express617**	**PACBIO**	**3,002,211 (78)**	**2,092**	**17,406,538**	**1,431**	**925 Mb**	**99,481**	**98.8%**
B. napus	**Tapidor3**	**PACBIO**	**2,855,025 (104))**	**5,318**	**11,486,821**	**3,566**	**1,014 Mb**	**105,409**	**98.4%**
B. napus	**Shengli3**	**PACBIO**	**2,825,656 (104)**	**6,024**	**14,590,814**	**3,802**	**1,002 Mb**	**103,920**	**98.1%**
B. napus	**QuintaA**	**PACBIO**	**2,801,289 (104)**	**5,835**	**14,779,276**	**3,722**	**1,004 Mb**	**98,755**	**98.4%**
B. napus	**No2127**	**PACBIO**	**2,704,645 (98)**	**5,787**	**12,981,515**	**3,733**	**1,012 Mb**	**105,894**	**98.0%**
B. napus	**GanganF73**	**PACBIO**	**2,696,026 (98)**	**6,887**	**18,404,683**	**4,930**	**1,032 Mb**	**106,567**	**98.4%**
B. napus	**Zheyou73**	**PACBIO**	**2,103,870 (126)**	**7,399**	**11,351,519**	**4,990**	**1,016 Mb**	**105,842**	**97.9%**
B. napus	**Zs11**	**PACBIO**	**1,506,624 (186)**	**8,773**	**10,640,036**	**3,332**	**1,010 Mb**	**107,233**	**98.5%**
B. rapa	Chiifu	PACBIO	1,386,548 (71)	1,507	9,417,752	1,099	353 Mb	60,609	99.6%

Table 4. Resistance Genes Analogs categories and repartition between chromosomes and unplaced scaffolds in *B. napus* Darmor-bzh v5 and v10 genome assemblies.

Annotation	RGA Location	RGA classes											Total
		NBS	CNL	TNL	CN	TN	NL	TX	Others	RLP	RLK	TM-CC	
Darmor-bzh v5 RGA	genome-wide	37	53	164	33	60	80	143	24	274	1502	418	2788
	pseudomolecules	27	45	144	27	54	67	112	22	231	1259	340	2328
	scaffolds	10	8	20	6	6	13	31	2	43	243	78	460
Darmor-bzh v10 RGA	genome-wide	45	71	189	22	58	85	128	29	285	1569	471	2952
	pseudomolecules	45	71	184	22	57	84	128	29	282	1561	471	2934
	scaffolds	0	0	5	0	1	1	0	0	3	8	0	18

Table 5. Repetitive element content of the *Brassica* long-read assemblies (ten polyploid *B. napus*, two diploid *B. rapa*, one diploid *B. nigra* and two diploid *B. oleracea* genomes).

	Total length (Mb)	Masked proportion	LTR Copia	LTR Gypsy	DNA CMC-EnSpm	LINE	RC Helitron	DNA TcMar	DNA MULE-MuDR
Darmor-bzh	924	53.86%	15.55%	13.05%	6.66%	6.37%	5.26%	2.05%	1.93%
Westar	1,008	58.91%	16.88%	13.38%	6.26%	6.92%	4.87%	1.92%	1.77%
Express617	925	56.32%	15.92%	12.46%	6.54%	6.63%	5.22%	2.05%	1.88%
Tapidor3	1,014	59.01%	16.49%	13.53%	6.17%	7.05%	4.86%	1.91%	1.76%
Shengli3	1,002	58.50%	16.15%	13.16%	6.16%	6.97%	4.86%	1.92%	1.78%
QuintaA	1,004	58.60%	16.40%	13.23%	6.29%	7.03%	4.93%	1.93%	1.80%
No2127	1,012	59.85%	17.08%	13.74%	6.23%	7.02%	4.77%	1.91%	1.72%
GanganF73	1,034	58.76%	16.60%	13.48%	6.23%	6.86%	4.82%	1.91%	1.75%
Zheyou73	1,016	59.06%	16.49%	13.59%	6.28%	7.04%	4.87%	1.91%	1.79%
Zs11	1,010	58.62%	16.59%	13.37%	6.36%	6.92%	4.88%	1.95%	1.78%
Z1	402	46.95%	13.22%	13.23%	2.88%	5.16%	4.37%	1.39%	1.53%
Chiifu	353	48.46%	12.89%	13.18%	3.29%	5.60%	5.15%	1.63%	1.82%
NI100	506	53.70%	16.51%	22.59%	4.08%	4.93%	3.17%	1.42%	1.79%
D134	530	57.88%	15.61%	13.23%	9.16%	6.24%	5.62%	2.56%	2.08%
HDEM	555	57.14%	16.06%	12.68%	8.74%	6.31%	5.42%	2.46%	1.99%

References

1. Jain M, Koren S, Miga KH, Quick J, Rand AC, Sasani TA, et al. Nanopore sequencing and assembly of a human genome with ultra-long reads. Nat Biotechnol [Internet]. Nat Biotechnol; 2018 [cited 2020 Jul 16];36. Available from: https://pubmed.ncbi.nlm.nih.gov/29431738/

2. Belser C, Istace B, Denis E, Dubarry M, Baurens FC, Falentin C, et al. Chromosome-scale assemblies of plant genomes using nanopore long reads and optical maps. Nature plants [Internet]. Nat Plants; 2018 [cited 2020 Jul 16];4. Available from: https://pubmed.ncbi.nlm.nih.gov/30390080/

3. -W. Schmidt MH, Vogel A, Denton AK, Istace B, Wormit A, van de Geest H, et al. De Novo Assembly of a New Solanum pennellii Accession Using Nanopore Sequencing. Plant Cell. American Society of Plant Biologists; 2017;29:2336–48.

4. Istace B, Friedrich A, d'Agata L, Faye S, Payen E, Beluche O, et al. de novo assembly and population genomic survey of natural yeast isolates with the Oxford Nanopore MinION sequencer. Gigascience [Internet]. Oxford Academic; 2017 [cited 2020 Jul 16];6. Available from: https://academic.oup.com/gigascience/article-pdf/6/2/giw018/25513794/giw018.pdf

5. Lv H, Wang Y, Han F, Ji J, Fang Z, Zhuang M, et al. A high-quality reference genome for cabbage obtained with SMRT reveals novel genomic features and evolutionary characteristics. Sci Rep. Nature Publishing Group; 2020;10:1–9.

6. CAAS_Brap_v3.01 - Genome - Assembly - NCBI [Internet]. [cited 2020 Jul 16]. Available from: https://www.ncbi.nlm.nih.gov/assembly/GCF_000309985.2

7. Perumal S, Koh CS, Jin L, Buchwaldt M, Higgins EE, Zheng C, et al. A high-contiguity Brassica nigra genome localizes active centromeres and defines the ancestral Brassica genome. Nat Plants. 2020;6:929–41.

8. Chalhoub B, Denoeud F, Liu S, Parkin IA, Tang H, Wang X, et al. Plant genetics. Early allopolyploid evolution in the post-Neolithic Brassica napus oilseed genome. Science [Internet]. Science; 2014 [cited 2020 Jul 16];345. Available from: https://pubmed.ncbi.nlm.nih.gov/25146293/

9. Bayer PE, Hurgobin B, Golicz AA, Chan CK, Yuan Y, Lee H, et al. Assembly and comparison of two closely related Brassica napus genomes. Plant Biotechnol J. Wiley-Blackwell; 2017;15:1602.

10. Bayer PE, Edwards D, Batley J. Bias in resistance gene prediction due to repeat masking. Nature Plants. Nature Publishing Group; 2018;4:762–5.

11. Song J-M, Guan Z, Hu J, Guo C, Yang Z, Wang S, et al. Eight high-quality genomes reveal pan-genome architecture and ecotype differentiation of Brassica napus. Nature Plants. Nature Publishing Group; 2020;6:34–45.

12. Lee H, Chawla HS, Obermeier C, Dreyer F, Abbadi A, Snowdon R. Chromosome-Scale Assembly of Winter Oilseed Rape Brassica napus. Front Plant Sci [Internet]. Frontiers; 2020 [cited 2020 Jul 16];11. Available from:

https://www.frontiersin.org/articles/10.3389/fpls.2020.00496/pdf

13. Verwoerd TC, Dekker BMM, Hoekema A. A small-scale procedure for the rapid isolation of plant RNAs [Internet]. Nucleic Acids Research. 1989. p. 2362–2362. Available from: http://dx.doi.org/10.1093/nar/17.6.2362

14. Alberti A, Poulain J, Engelen S, Labadie K, Romac S, Ferrera I, et al. Viral to metazoan marine plankton nucleotide sequences from the Tara Oceans expedition. Sci Data. 2017;4:170093.

15. fastxtend [Internet]. [cited 2020 Jul 16]. Available from: http://www.genoscope.cns.fr/externe/fastxtend/

16. Kim D, Song L, Breitwieser FP, Salzberg SL. Centrifuge: rapid and sensitive classification of metagenomic sequences. Genome Res. Cold Spring Harbor Laboratory Press; 2016;26:1721.

17. Arcade A, Labourdette A, Falque M, Mangin B, Chardon F, Charcosset A, et al. BioMercator: integrating genetic maps and QTL towards discovery of candidate genes. Bioinformatics. 2004;20:2324–6.

18. Delourme R, Falentin C, Fomeju BF, Boillot M, Lassalle G, André I, et al. High-density SNP-based genetic map development and linkage disequilibrium assessment in Brassica napus L. BMC Genomics. 2013;14:120.

19. Clarke WE, Higgins EE, Plieske J, Wieseke R, Sidebottom C, Khedikar Y, et al. A high-density SNP genotyping array for Brassica napus and its ancestral diploid species based on optimised selection of single-locus markers in the allotetraploid genome [Internet]. Theoretical and Applied Genetics. 2016. p. 1887–99. Available from: http://dx.doi.org/10.1007/s00122-016-2746-7

20. Kumar V, Paillard S, Fopa-Fomeju B, Falentin C, Deniot G, Baron C, et al. Multi-year linkage and association mapping confirm the high number of genomic regions involved in oilseed rape quantitative resistance to blackleg [Internet]. Theoretical and Applied Genetics. 2018. p. 1627–43. Available from: http://dx.doi.org/10.1007/s00122-018-3103-9

21. de Givry S, Bouchez M, Chabrier P, Milan D, Schiex T. CARHTA GENE: multipopulation integrated genetic and radiation hybrid mapping. Bioinformatics. 2005;21:1703–4.

22. Johnston JS, Pepper AE, Hall AE, Chen ZJ, Hodnett G, Drabek J, et al. Evolution of Genome Size in Brassicaceae. Ann Bot. Oxford Academic; 2005;95:229–35.

23. Boutte J, Maillet L, Chaussepied T, Letort S, Aury J-M, Belser C, et al. Large genomic variants reveal unexplored intraspecific diversity in Brassica rapa genomes [Internet]. bioRxiv. 2020 [cited 2020 Jul 17]. p. 2020.07.02.183640. Available from: https://www.biorxiv.org/content/10.1101/2020.07.02.183640v1.abstract

24. Ruan J, Li H. Fast and accurate long-read assembly with wtdbg2. Nat Methods. 2020;17:155–8.

25. ruanjue. ruanjue/smartdenovo [Internet]. GitHub. [cited 2020 Jul 16]. Available from: https://github.com/ruanjue/smartdenovo

26. Kolmogorov M, Yuan J, Lin Y, Pevzner PA. Assembly of long, error-prone reads using

repeat graphs. Nat Biotechnol. 2019;37:540–6.

27. rrwick. rrwick/Filtlong [Internet]. GitHub. [cited 2020 Jul 16]. Available from: https://github.com/rrwick/Filtlong

28. Vaser R, Sović I, Nagarajan N, Šikić M. Fast and accurate de novo genome assembly from long uncorrected reads. Genome Res. 2017;27:737–46.

29. Walker BJ, Abeel T, Shea T, Priest M, Abouelliel A, Sakthikumar S, et al. Pilon: an integrated tool for comprehensive microbial variant detection and genome assembly improvement. PLoS One. 2014;9:e112963.

30. Istace B, Belser C, Aury J-M. BiSCoT: Improving large eukaryotic genome assemblies with optical maps [Internet]. Available from: http://dx.doi.org/10.1101/674721

31. Kurtz S, Phillippy A, Delcher AL, Smoot M, Shumway M, Antonescu C, et al. Versatile and open software for comparing large genomes. Genome Biol. BioMed Central; 2004;5:1–9.

32. gtrichard. gtrichard/mummer-ggplot [Internet]. GitHub. [cited 2020 Jul 16]. Available from: https://github.com/gtrichard/mummer-ggplot

33. Quinlan AR, Hall IM. BEDTools: a flexible suite of utilities for comparing genomic features. Bioinformatics. 2010;26:841–2.

34. Xu L, Seki M. Recent advances in the detection of base modifications using the Nanopore sequencer. J Hum Genet. Nature Publishing Group; 2019;65:25–33.

35. Li H. Minimap2: pairwise alignment for nucleotide sequences. Bioinformatics [Internet]. Bioinformatics; 2018 [cited 2020 Jul 16];34. Available from: https://pubmed.ncbi.nlm.nih.gov/29750242/

36. Broseus L, Thomas A, Oldfield AJ, Severac D, Dubois E, Ritchie W. TALC: Transcript-level Aware Long Read Correction. Bioinformatics [Internet]. 2020 [cited 2020 Jul 21]; Available from: https://academic.oup.com/bioinformatics/article-pdf/doi/10.1093/bioinformatics/btaa634/33504044/btaa634.pdf

37. Morgulis A, Gertz EM, Schäffer AA, Agarwala R. A fast and symmetric DUST implementation to mask low-complexity DNA sequences. J Comput Biol. 2006;13:1028–40.

38. RepeatMasker Home Page [Internet]. [cited 2020 Jul 16]. Available from: http://repeatmasker.org

39. Hurgobin B, Golicz AA, Bayer PE, Chan C-KK, Tirnaz S, Dolatabadian A, et al. Homoeologous exchange is a major cause of gene presence/absence variation in the amphidiploid Brassica napus. Plant Biotechnol J. 2018;16:1265–74.

40. Dolatabadian A, Bayer PE, Tirnaz S, Hurgobin B, Edwards D, Batley J. Characterization of disease resistance genes in the Brassica napus pangenome reveals significant structural variation. Plant Biotechnol J. 2020;18:969–82.

41. Kent WJ. BLAT--the BLAST-like alignment tool. Genome Res. 2002;12:656–64.

42. Birney E, Clamp M, Durbin R. GeneWise and Genomewise. Genome Res.

2004;14:988–95.

43. Sessegolo C, Cruaud C, Da Silva C, Cologne A, Dubarry M, Derrien T, et al. Transcriptome profiling of mouse samples using nanopore sequencing of cDNA and RNA molecules [Internet]. Scientific Reports. 2019. Available from: http://dx.doi.org/10.1038/s41598-019-51470-9

44. Mott R. EST_GENOME: a program to align spliced DNA sequences to unspliced genomic DNA. Comput Appl Biosci. 1997;13:477–8.

45. Dubarry M, Noel B, Rukwavu T, Farhat S, Da Silva C, Seeleuthner Y, et al. Gmove a tool for eukaryotic gene predictions using various evidences. F1000Res [Internet]. 2016 [cited 2020 Jul 16];5. Available from: http://dx.doi.org/10.7490/f1000research.1111735.1

46. Seppey M, Manni M, Zdobnov EM. BUSCO: Assessing Genome Assembly and Annotation Completeness. Methods Mol Biol [Internet]. Methods Mol Biol; 2019 [cited 2020 Jul 16];1962. Available from: https://pubmed.ncbi.nlm.nih.gov/31020564/

47. Mason AS, Rousseau-Gueutin M, Morice J, Bayer PE, Besharat N, Cousin A, et al. Centromere Locations in Brassica A and C Genomes Revealed Through Half-Tetrad Analysis. Genetics. 2016;202:513–23.

48. Altschul SF, Gish W, Miller W, Myers EW, Lipman DJ. Basic local alignment search tool. J Mol Biol [Internet]. J Mol Biol; 1990 [cited 2020 Jul 16];215. Available from: https://pubmed.ncbi.nlm.nih.gov/2231712/

49. Li P, Quan X, Jia G, Xiao J, Cloutier S, You FM. RGAugury: a pipeline for genome-wide prediction of resistance gene analogs (RGAs) in plants. BMC Genomics. BioMed Central; 2016;17:1–10.

50. yaanlpc / RGAugury / wiki / Home — Bitbucket [Internet]. [cited 2020 Jul 16]. Available from: https://bitbucket.org/yaanlpc/rgaugury/wiki/Home

51. Li Y, Zhong Y, Huang K, Cheng Z-M. Genomewide analysis of NBS-encoding genes in kiwi fruit (Actinidia chinensis). J Genet. 2016;95:997–1001.

52. Perumal S, Waminal NE, Lee J, Lee J, Choi B-S, Kim HH, et al. Elucidating the major hidden genomic components of the A, C, and AC genomes and their influence on Brassica evolution [Internet]. Scientific Reports. 2017. Available from: http://dx.doi.org/10.1038/s41598-017-18048-9

53. Parker MT, Knop K, Sherwood AV, Schurch NJ, Mackinnon K, Gould PD, et al. Nanopore direct RNA sequencing maps the complexity of Arabidopsis mRNA processing and m6A modification. eLife Sciences Publications Limited; 2020 [cited 2020 Jul 16]; Available from: https://elifesciences.org/articles/49658

54. Workman RE, Tang AD, Tang PS, Jain M, Tyson JR, Razaghi R, et al. Nanopore native RNA sequencing of a human poly(A) transcriptome. Nat Methods. Nature Publishing Group; 2019;16:1297–305.

55. Darracq A, Adams KL. Features of evolutionarily conserved alternative splicing events between Brassica and Arabidopsis. New Phytol. 2013;199:252–63.

56. Brunson JC. Alluvial Plots in “ggplot2” [R package ggalluvial version 0.12.0].

Comprehensive R Archive Network (CRAN); [cited 2020 Jul 20]; Available from: https://CRAN.R-project.org/package=ggalluvial

57. Ramírez F, Bhardwaj V, Arrigoni L, Lam KC, Grüning BA, Villaveces J, et al. High-resolution TADs reveal DNA sequences underlying genome organization in flies. Nat Commun. Nature Publishing Group; 2018;9:1–15.

58. Zhang L, Cai X, Wu J, Liu M, Grob S, Cheng F, et al. Improved reference genome by single-molecule sequencing and chromosome conformation capture technologies. Hortic Res. 2018;5:50.

Click here to access/download
Supplementary Material
BrassicaNapus_genome_SupData.pdf

Supplementary Table S14

Click here to access/download
Supplementary Material
Table S14 - Darmor-bzh pericentromeric regions.xlsx

Click here to access/download
Supplementary Material
Table S15 - Darmor-bzh large misassembled regions.xlsx

Supplementary Table S16

Click here to access/download

Supplementary Material

Table S16 - Darmor-Bzh v5 RGA positions.xlsx

Supplementary Table S17

Click here to access/download

Supplementary Material

Table S17 - Darmor-Bzh v10 RGA positions.xlsx

Supplementary Table S18

Click here to access/download

Supplementary Material

Table S18 - Darmor-bzh v5 v10 correspondance.xlsx

Personal Cover Click here to access/download;Personal Cover;Cover Letter.pdf

Author's covering letter for resubmission

Dr Laurie Goodman
Editor-in-chief
Gigascience

18 September 2020

Dear Dr Goodman,

Please find enclosed our revised manuscript "Long-reads assembly of the *Brassica napus* reference genome, Darmor-bzh".

First of all, we would like to thank the three reviewers for their meticulous reading of the manuscript. We have taken into account their comments, and believe that this new version of the manuscript is now of better quality. The GigaDB repository has been enriched with numerous files, which should be of great interest to reviewers and readers.

During the first submission, we put the article on the BioRxiv server and received some feedback from the scientific community showing the strong interest in this work. We have received questions regarding the correspondence between genes of the v5 and v10 assemblies. We have decided to provide a correspondence table with this manuscript (Table S18) and have added a description of our method and results in a dedicated section of the manuscript.

We hope that this new version of the manuscript will be suitable for publication in Gigascience, and are certain that this most contiguous and complete genome assembly of *Brassica napus* will be a valuable resource for crop improvement and will facilitate the rapid selection of agronomically important traits.

The manuscript has been reviewed and approved by all listed authors and all the data are publicly available from the ENA database under the following accession numbers PRJEB39416 and PRJEB39508 or from our website : http://www.genoscope.cns.fr/plants

We look forward to hearing from you at your earliest convenience, and we welcome any comments that you and your reviewers may have.

Yours sincerely,

Jean-Marc Aury

Response to the reviewers Click here to access/download;Personal Cover;Response to reviewers.pdf

First of all, we would like to thank the three reviewers for their meticulous reading of the manuscript. We have taken into account their comments, and believe that this new version of the manuscript is now of better quality. During the first submission, we put the article on the BioRxiv server and received some feedback from the scientific community, especially regarding the correspondence between genes of the v5 and v10 assemblies. We have decided to provide a correspondence table with this manuscript (Table S18) and have described our method and results in a dedicated section of the manuscript.

Reviewer #1:

The authors described a new Brassica napus genome assembly and compared it to other Brassica genomes, particularly including the older version of the same genotype. The comparisons in terms of gaps, anchored markers, anchored BUSCOs, gene annotation and RGA not only show solid evidence of improvement, but also provide the Brassica community with important information to transit from the older assembly version.

The manuscript is well-written with logical flow of methods and conclusions. I particularly enjoyed the emphasis on detailed explanation of technical methods, including choice of parameters and filtering criteria, because they enable readers to easily decide whether the results make sense. It also shows that the analyses were done with careful thoughts and thorough optimization.

Below are some minor comments, as well as suggestions that may or may not be addressed by the authors:

1. How well do the Illumina reads (ERX397788 and ERX397800) align to the Darmor v10, since they originated from data of a few years ago? This information is not crucial because 1) they were not directly used for gene prediction, and 2) TALC-corrected reads mapped better, but could be useful to judge the correctness of TALC outcome.
We aligned the Illumina reads on the v5 and v10 genome assemblies and observed a higher proportion of aligned reads on the v10 genome: 89.15% vs 88.42% for ERX397788 and 89.62% vs 88.51% for ERX397800. The difference is slight but in favor of the v10 assembly.

2. Line "These misassembled regions were validated using the new ZS11 assembly as a control (Figure 2, highlighted in purple on the C07 chromosome)": it looks like green instead?
We replaced purple with green in the text.

3. Figure 2: positions where pericentromeric-flanking markers aligned could be indicated, to further highlight the additionally assembled/inversed regions.
We have added in Figure 2 the alignment positions of pericentromeric-flanking markers.

4. Are alternatively spliced transcripts provided? File "BnapusDarmor-bzh_annotation.gff" only contains primary transcripts.
The alternative transcripts were not predicted, at each loci a single transcript was annotated. However, we have now added requested files that contain alternative splicing events (intron retention and exon skipping) in the GigaDB repository.

5. Table S14 and S15: column names are missing
Column names of Tables S14 and S15 have been added.

6. Table S1: accession numbers are missing (probably pending, just a reminder)
Accession numbers have now been added to Table S1.

Reviewer #2:

In this Data Note, Rousseau-Gueutin et al. describe a new genome assembly and annotation of the allotetraploid Brassica napus Darmor-bzh. The assembly is based on Nanopore sequencing combined with an Bionano optical map. Previous assemblies are based on older technologies and issues with assembly and annotation quality make an upgraded reference genome and annotation a welcome and valuable resource for the Brassica research community.

I do have a number of comments, however, my copy of the manuscript does not have line numbers so I will refer to page numbers in the text.

Comments:
Pg 3 "Background" - the manuscript starts off with a well written background on the relationships of the Brassica genomes and a review of the genome sequencing of Brassica genomes, and status and issues of the B. napus Darmor-bzh v5 and v8 assemblies.
Thanks for your positive feedback.

Pg 5 "These trimming and removal steps were achieved using in-house-designed software based on the FastX package" - since the software is open source and in Github, just use its name (fastxtend) and parameters and version or commit id used for the analysis
We have changed the text accordingly and replaced "... were achieved using in-house-designed software based on the FastX package" with "... were achieved using Fastxtend tools".

Pg 6 "A taxonomical assignation was performed using Centrifuge [16] for each dataset to detect potential contaminations." - needs more information (version, parameters, database) and report the results from the centrifuge run.
We added the centrifuge version, parameters and database information, as well as an overview of the result in the corresponding section : "On each run, approximately 65% of the reads were unassigned and almost 35% were assigned to the Brassica genus. In addition, we detected a small proportion (<0.6%) of reads corresponding to various metazoa."

Pg 7 "In order to get the best possible genome assembly" -
I found this paragraph odd, especially in the context of a data note. There is no rationale for why these three assemblers were chosen other than "to get the best possible genome assembly" and of course, I could immediately think of another 6 assemblers you could have tried for achieve this goal. Flye is a fine choice, the results are excellent, so I would recommend modifying this paragraph and modifying the table to just report the assembly of the final contigs with Flye
We agree with the reviewer's comment regarding the formulation "to get the best possible genome assembly". We changed the sentence and replaced it with "We used three different assemblers…". However, we have decided to keep the results of the three assemblers in supplementary data as we believe it may be useful for readers and especially for people working on genome assembly.

Pg7 "As nanopore reads contain systematic error" - I'm surprised that the assembly was not also polished with a tool that specializes in polishing nanopore assemblies such as Nanopolish or Medaka, especially as Nanopolish was used for the calling of 5mC bases from the raw WGS reads.
By the time we did the assembly years ago, nanopolish was too slow to work on large genomes and medaka was at an early stage of development. That's why we decided to use classic polishing tools like Racon and Pilon.

Pg 9 "Detection of modified bases" - This section showcases the calling from 5mCs from raw nanopore reads, however the results seem only to summarized as a track on Figure 1. This is fine, but the raw data for the track generated with what I assume is nanopolish call-methylation and the data filtered as described should be included in the data repository accompanying the paper
We added two files to the GigaDB repository. The first is the raw file, a tabular file with eleven columns that contain the nanopolish results for each nanopore read. The second is a tabular file with four columns used for generating the graph in Figure 1.

"We analysed the electric signal to detect the" - odd phrasing, rewrite to state the method used
We agree with the reviewer and decided to delete this sentence which was not informative.

Pg 10 - Gene Prediction - the gene annotation utilized a range of protein evidence along with the direct RNA reads aligned to the genome (using some venerable "old school" aligners!). Gene models were created using gmove, a tool I was not familiar with, but appears to be similar in function to EVM or MAKER to generate a consensus gene model. I'd like the authors to comment on why a de novo approach to annotation was not taken and why the direct RNA sequencing was not leveraged to predict isoforms.
Indeed, genewise and est2genome are "old school" aligners, but we agree that they are still very efficient in terms of alignment quality. We decided not to use a de novo approach, as the number of biological resources was high and specific genes could have been annotated using RNA-Seq from the same genotype. We did not predict the isoforms because we were concerned that the corrected nanopore reads might contain unreal isoforms. We believe that isoform prediction using long noisy reads is not mature enough, but this will undoubtedly be an important point to exploit while relying on future methods.

Pg 11 - "Brassica (CentBr1, CentBr2, CRB, PCRBr, TR238 and TR805) which were blasted" - Changed 'blasted' to 'searched'
We have modified the text accordingly.

Pg 12 - Comparison with existing assemblies and annotations
Throughout the manuscript, BUSCO results are not reported using the summary string recommended by the BUSCO developers. Also, the short_summary.*.txt BUSCO results file for the genome and annotation is missing from the data repo. As B. napus is an allotetraploid, I would like to see how many BUSCOs are complete and duplicated vs single.
This is a good point, we have added the full summary string in Table 1 for the three genomes of Darmor-bzh. However, since Table 3 is already dense, we prefered to create a

new table in the supplementary Information for these additional results (Table S9). In addition, we have added all the BUSCO short_summary.*.txt in the GigaDB repository.

Pg 15 Alternative splicing events - The data describing the IR and skipped exon events in the results is not present in the data repo and should be added.
We have added requested files that contain alternative splicing events (intron retention and exon skipping) in the GigaDB repository.

General comment - Gigascience has set a high bar for reporting software tool versions and command line parameters to promote reproducibility. This information is missing throughout the manuscript and needs to be added. Data referred to in the results should also be added to the data repo accompanying the paper.
We have tried to be exhaustive, and we have added the version and parameters for each software we have used. In addition, the GigaDB repository now contains all the results generated for this publication.

Reviewer #3:

The authors present an improved version of the Darmor-bzh (Brassica napus) reference genome and annotation using a combination of long-reads sequencing data, optical and genetic maps. They report a detailed comparison of the new Darmor-bzh v10 genome assembly and annotation with previous releases of the same genome (evaluating contiguity, gene and repeat content, Resistance Genes Analogs) as well as with the other available Brassica assemblies. While they provide a valuable resource for the plant community, I have several major and minor comments.

Major comments

1) The assembly size of Darmor-bzh v10 reported in this paper (924 Mb) is larger than the previous releases v5 and v8 (850 Mb). Since the real size of the genome is unknown, it would be interesting to provide a genome size estimation based on k-mer analysis of Illumina reads or flow cytometry. The authors did not report the genome size parameter used to assemble the reads with Redbean and Flye. In Supplementary Table 1, they computed the coverage based on an estimated genome size of 1.2 Gb but it is unclear why they chose this value.

We chose the value of 1.2Gb because the genome size was first estimated at 1,132 Mb by Johnston *et al.* (https://doi.org/10.1093/aob/mci016) using flow cytometry. However, the k-mer spectrum (k=31) provides a lower estimate of 862Mb. We have added a paragraph that gives information on genome size estimation in the “Genome assembly” section.

2) Could you please provide more details about the criteria used to select the 'best' assembly? On page 7, the authors report that: 'Then, we selected the 'best' assembly based not only on contiguity metrics such as N50 but also cumulative size. The Flye assembler using the longest reads produced the most contiguous assembly and lead to a contig N50 of 10.0Mb.' My understanding is that the Flye assembly using the longest reads (ie only 30X instead of the 93X of data generated) was selected as the 'best' assembly because of its higher contig N50 and maybe also because of its cumulative size? (if yes, what criteria were used for the cumulative size?). Is that correct?

Yes you are right, the Flye (longest reads) and smartdenovo (all reads) assemblies were very similar in terms of contiguity (N50 and N90), but even though the N50 was slightly better using smartdenovo, we decided to keep the Flye assembly as its cumulative size was higher. We have now changed the text to explain our choice.

- The criteria to decide on a best assembly could include other metrics such as gene content and k-mer spectra. Have you estimated the BUSCO completeness for the different assemblers presented in Supplementary Table 2? A comparison of the k-mer spectrum of the assembly to the k-mer spectrum of the Illumina reads could be presented as well.

In general, the BUSCO score is a good metric for comparing assemblies, but in the case of unpolished assemblies the results are difficult to interpret. We added the BUSCO scores in Table S2, and the completeness scores were between 28.3% and 44.3% for wtdbg2, 54.2% and 90.9% for smartdenovo and 76.7% and 78.7% for Flye. In this case, the metrics reflect the accuracy of the polishing step of each assembler. For the same reason, the KAT plot is

biased due to the consensus accuracy of each assembler. We have added the KAT plot for the raw and polished Flye assemblies in Figure S2.

- With Smartdenovo, adding more coverage resulted in a larger assembly with a higher contig N50. The authors reported in Supplementary Table 2 that the Flye assembly using all the reads "crashed". It would be interesting to see the Flye assembly results using 50 or 60X coverage, if the full dataset cannot be assembled. The Flye parameter --asm-coverage could be used to reduce the memory requirements of the program during the initial contig extension stage.
Indeed, the input dataset can be sampled in different ways, but generating numerous assemblies is time consuming. When we started the project we decided to generate three subsets of reads and use three different assemblers. Out of curiosity we ran the flye assembler with 50X of ONT reads, and got an assembly with a contig N50 of 9.4Mb (31 contigs), a contig N90 of 321Kb (209 contigs) and a cumulative size of 963Mb. So even though the cumulative size is higher, the N90 is twice as low as the selected assembly.

3) Long read sequencing technologies are a powerful tool to study alternative splicing and this is an area of active development. The 'Alternative splicing events' section could include additional analysis or discussion.
We first generated the direct RNA dataset to improve gene prediction and not to examine splicing events although this type of data opens up new perspectives in this area. Here we have pooled RNAs from two different tissues (leaf and root) and have not generated replicates which limits the applicative interest of this dataset.

- Have you tried to assess the confidence in the splicing events detected using the ONT long reads? It could be interested to try an alternative method or to include a comparison with the splicing events identified with the Illumina short reads.
To date, there is no mature software available that deals with ONT reads to detect splicing events. However, we have used the illumina dataset and run the same type of analysis to compare the intron retention detected by the two technologies. We find a good overlap between the two methods by allowing a low coverage of Illumina reads (>5). We have modified the corresponding section.

- The reported intron retention percentage in Darmor-Bzh v10 (16%) is much smaller than what was detected in Darmor-Bzh v5 (62%, Chalhoub et al., 2014). Intron retention detection is challenging but this could be investigated or discussed.
In Chalhoub et al., 62% refers to the proportion of IR events among the gene having splicing events (48%), showing that IR events are in the majority. The correct numbers are 29% and 16%, and the smaller difference can be explained by the lower sequencing depth of the nanopore dataset. We have added the points described above in the corresponding section.

- Could you provide the list of splicing events as a supplementary table?
We have added requested files that contain alternative splicing events (intron retention and exon skipping) in the GigaDB repository.

Minor comments

- It would be helpful to provide a schematic diagram summarising the genome assembly, scaffolding and annotation steps and including all the datasets and tools used to obtain the final v10 assembly.
We have added as a Supplementary Figure (Figure S3), a schematic diagram describing the different steps for obtaining the final assembly and the gene prediction.

- p3: The GBS abbreviation should be explained.
We have modified the text accordingly.

- p5-6: Nanopore sequencing sections. Which flow cell type was used?
For genomic and RNA sequencing, we used R9.4.1 flowcells. This information has been added to the nanopore sequencing sections.

- p6: Which basecalling tool was used ? Please also indicate the version and parameters used.
Genomic reads were basecalled using the guppy basecaller (version 1.4.3) with the 'fast' configuration and default parameters. RNA-Seq reads were basecalled using the guppy basecaller (version 3.2.8) with the 'hac' configuration and default parameters. We have added the missing information in the corresponding sections.

- p7: Long reads genome assembly section. Which version of each assembler and polishing tool were used?
We have added the assemblers' version in the corresponding section.

- p7: Is there a reason for choosing to perform three iterations of Racon and three iterations of Pilon? For the selected assembly, it would be interesting to report the assembly metrics after each round of polishing including genome completeness estimation.
This is an interesting remark, we have followed the observation reported on the first plant genome assembled with nanopore data (https://dx.doi.org/10.1105%2Ftpc.17.00521). This study recommended performing three iterations of Racon, followed by three iterations of Pilon. We ran the BUSCO analysis on each intermediate assembly to look at the gain of each iteration. We have now added these results to Table S3.

- p8: "As already reported, we found in several cases that the nanopore contigs were overlapping (based on the optical map)". Please include a citation of where this has been already reported.
We now cite the following article : https://doi.org/10.1038/s41477-018-0289-4.

- p8-10: Please also include the versions used for the tools mentioned ie nucmer, TALC etc.
We have added the software's versions in the corresponding section.

- p8-9: Large Misassembled Inverted regions section: Is the script to identify the LMI provided?
We have added the script (get_LMIs.sh) to the GigaDB repository.

- p13: "(Figure 2, highlighted in purple on the C07 chromosome)". I cant see any purple in Fig2 in the C07 chromosome plot, do you mean green?
We replaced purple with green in the text.

- p13: 'the proportion of anchored sequence (from 765 Mb for Express617 to 961 Mb for Zs11)'. The term proportion is mentioned but the reported numbers correspond to sizes.
We changed the text, and replaced 'the proportion of anchored bases' with 'the number of anchored bases'.

- p13: "The eleven Brassica genomes sequenced using PACBIO have a contig N50 between 1.4Mb and 3.6Mb whereas all the ONT assemblies have a contig N50 higher than 5.5Mb." I would replace "all the ONT" by "the four ONT" assemblies.
We replaced "all the ONT" with "the four ONT" assemblies.

- p14: A word is missing in the sentence "As a comparison, we found 5,460 in the PACBIO assembly of the Zs11 genotype."
We have added the missing word "gaps".

- p14, p16. Interestingly, the authors reported an example of a large transposable element resolved thanks to ONT ultra long reads (as compared to a gap in the PacBio+HiC assembly). They also assembled a filtered subset of reads ('Filtlong reads' including 2X >100 kb). Have you tried to assemble the ONT data without including the ultra long reads at all (ie 6X >100 kb)? It would be interesting to see the impact of those ultra-long reads on the overall assembly quality, especially because it could be challenging to generate ultra long reads and this is a specificity of the ONT technology.
As recommended we ran an assembly using Flye but without the ultra-long reads, and got an 894Mb assembly composed of 4,590 contigs, with a contig N50 of 9.3Mb and N90 of 349Kb. The N50 is still very high, but the assembly contains almost 3 times the number of contigs of the assembly obtained with ultra-long reads (4,590 vs 1,594). The assembly at the scaffold level will necessarily contain a higher number of gaps indicating the importance of long-reads.

- page 15 "From the genomic alignments of raw reads, with an identity percent higher than 90%". Which strategy was used to align the reads ?
Alignments were performed with Est2Genome, we have added the following description in the corresponding section : "Raw reads were aligned on the genome in a two-steps strategy. First, BLAT (version 36 with default parameters) was used to fastly localize corresponding putative regions of these RNA reads on the genome. The best match for each read was selected and a second alignment was performed using Est2Genome (version 5.2 with default parameters)."

Tables
- p26-27. Tables 1 and 2. I would add a column in those tables to include the reference paper and the corresponding release date for each assembly.
Table 1 and 2 are already dense, we preferred to create a new table that includes global information like the reference paper and the release date.

Supplementary Tables
- Supplementary Table 1. The accession numbers are missing.
Accession numbers have now been added to Table S1.

- Supplementary Table 2:
-The authors reported that the Flye assembly using all the reads "crashed". Was it a memory issue due to the computing resources used?
No it was not a memory issue. In our experience, the earlier versions of Flye were not very stable and assemblies often crashed for no easily identifiable reasons. Flye is now much more stable and crashes are very rare thanks to the many updates that its authors have made.

-I would add the BUSCO completeness estimation for each of those assemblies.
Generally the BUSCO score is a great metric to compare assemblies, but in the case of unpolished assemblies the results are difficult to interpret. We added the BUSCO scores in Table S2, and the completeness scores were between 28.3% and 44.3% for wtdbg2, 54.2% and 90.9% for smartdenovo and 76.7% and 78.7% for Flye. In this case, the metrics reflect the accuracy of the polishing step of each assembler.

- Supplementary Table 8. Could you please add in the legend that 'Comp' refers to Comparative genomics (Long-range technology column)?
We added the following sentence in the legend : " In the long-range technology column, "Comp" refers to comparative genomics meaning that contigs have been organized using synteny with an existing assembly at the chromosome-level ."
