# Supplementary material for: Long-read assembly of the Brassica napus reference genome Darmor-bzh
Source: Gigascience. 2020 Dec 15;9(12):giaa137. doi: 10.1093/gigascience/giaa137 (PMC7736779; doi:10.1093/gigascience/giaa137)

Supplementary Table 1. Statistics of the sequencing dataset for *B. napus* Darmor-bzh

	ONT (6 PromethION runs)			Illumina
	Raw reads	Longest reads	Filtlong reads	1.5 lane of HS2500 Rapid Run
Cumulative size	111.9 Gb	36.1 Gb	36 Gb	97 Gb
# of reads	15,831,026	462,512	712,158	215,472,682
Coverage (estimated genome size 1.2Gb)	93X	30X	30x	81X
Coverage Reads >100Kb (estimated genome size 1.2Gb)	5.75X	5.75X	1.97X	NA
N50 (bp)	42,806	76,025	55,397	NA
Longest (bp)	1,084,288	1,084,288	994,661	250
Accession numbers	PRJEB39416	NA	NA	PRJEB39416

Supplementary Table 2. *B. napus* Darmor-bzh ONT assembly statistics.

Assembler	Subset of reads used	Cumulative Size	# contigs	N50 (L50)	N90 (L90)	CPU time (h)	BUSCO scores (n=1,614)
Smartdenovo	All reads	916,568,779	731	**10,218,000** (31)	597,059 (156)	7,483	C:90.9% S:52.0% D:38.9% F:4.0% M:5.1%
	Filtlong reads	835,563,225	**625**	4,161,078 (53)	597,575 (238)	2,079	C:87.6% S:54.0% D:33.6% F:5.2% M:7.2%
	Longest reads	819,736,553	773	2,041,018 (107)	448,924 (428)	2,863	C:54.2% S:47.0% D:7.2% F:18.8% M:27.0%
Redbean	All reads	1,870,584,980	53,028	63,466 (8,388)	14,710 (31,095)	243	C:28.3% S:25.8% D:2.5% F:17.0% M:54.7%
	Filtlong reads	1,476,953,275	27,745	109,469 (3,407)	23,825 (14,495)	**176**	C:44.3% S:38.8% D:5.5% F:18.6% M:37.1%
	Longest reads	1,273,533,804	21,319	127,500 (2,252)	127,500 (2,252)	184	C:40.2% S:36.5% D:3.7% F:18.0% M:41.8%
Flye	All reads	-	-	-	-	crashed	-
	Filtlong reads	927,471,373	1,516	8,067,213 (34)	**616,556** (165)	1,632	C:78.7% S:53.2% D:25.5% F:10.2% M:11.1%
	Longest reads	937,929,502	1,594	10,072,792 **(29)**	561,876 **(146)**	1,472	C:76.7% S:55.1% D:21.6% F:11.5% M:11.8%

Supplementary Table 3. Iterative polishing of the Flye assembly.

	Raw	Racon 1	Racon 2	Racon 3	Pilon 1	Pilon 2	Pilon 3
Cumul. (Mb)	937.9	930.0	929.6	929.4	929.8	929.5	929.4
N50 (L50)	10,072,792 (29)	9,979,042 (29)	9,973,949 (29)	9,973,407 (29)	9,974,728 (29)	9,973,208 (29)	9,972,764 (29)
N90 (L90)	561,876 (146)	555,231 (146)	555,820 (146)	557,055 (146)	558,600 (146)	553,038 (146)	553,072 (146)
Complete Busco	76.7%	96.4%	96.7%	96.9%	99.4%	99.5%	99.5%
Duplicated Busco	21.6%	60.0%	61.5%	61.6%	86.7%	91.6%	91.9%
Fragmented Busco	11.5%	1.2%	1.2%	0.7%	0.2%	0.1%	0.1%
Missing Busco	11.8%	2.4%	2.1%	2.4%	0.4%	0.4%	0.4%

Supplementary Table 4. *B. napus* Darmor-bzh bionano dataset (raw molecules)

	BspQI molecules	DLE-1 molecules
Number of molecules	2,124,074	16,646,860
Total length (Mb)	223,164	1,340,608
Average length (Kb)	105	81
Molecule N50 (Kb)	178	101
Label density (/100Kb)	7.7	16.6
Number of flowcells	2	1

Supplementary Table 5. *B. napus* Darmor-bzh bionano dataset (filtered molecules)

	BspQI molecules	DLE-1 molecules
Number of molecules	402,272	1,699,106
Total length (Mb)	101,656	397,015
Average length (Kb)	253	234
Molecule N50 (Kb)	257	229
Label density (/100Kb)	9.4	16.9

Supplementary Table 6. *B. napus* Darmor-bzh optical maps

	BspQI map	DLE-1 map
Number of maps	868	178
Total map length (Mb)	1,042	968
Map N50 (Mb)	1.7	18.2

Supplementary Table 7. *B. napus* Darmor-bzh hybrid scaffolding and polishing

	ONT polished assembly (contigs)	Optical maps integration (scaffolds)	BiSCoT (contigs)	BiSCoT (scaffolds)
Cumulative size (bp)	929,388,171	957,271,942	907,852,133	954,769,412
# sequences	1,869	1,432	1,581	1,378
N50 (L50)	9,972,764 (29)	22,450,564 (15)	11,241,391 (25)	22,666,588 (15)
N90 (L90)	553,072 (146)	2,973,865 (53)	718,812 (111)	3,083,366 (52)
Maximum size (bp)	34,824,884	48,039,605	46,624,681	47,917,037
Number of N's (%)	0%	28,143,795 (2.94 %)	0%	25,966,328 (2.72%)

Supplementary Table 8. *B. napus* Darmor-bzh chromosomal organization

	19 chromosomes	Unanchored scaffolds	A genome	C genome
Cumulative size (bp)	866,915,903	56,879,860	346,465,994	520,449,909
# sequences	19	218	10	9
N50 (L50)	53,549,824 (7)	652,631 (15)	39,685,748 (4)	62,297,340 (4)
# contigs	505	-	157	78
Contig N50 (L50)	11,486,274 (24)	-	9,804,805 (11)	15,347,254 (12)
N90 (L90)	29,390,524 (16)	93,503 (142)	23,101,716 (9)	48,239,360 (7)
Maximum size (bp)	73,669,886	4,834,143	53,549,826	73,669,886
Number of N's (%)	17,515,951 (2.20%)	8,475,880 (14.90%)	12,445,409 (3.59%)	5,070,542 (0.97%)

Supplementary Table 9. Statistics of the *Brassica* long-reads assemblies (ordered by contigs N50 value, *B. napus* are in bold).

	genotype	technology	contigs N50 (L50)	cumulative size	Number of genes	BUSCO scores n=4596
B. nigra	NI100	ONT	11,504,526 (13)	506 Mb	59,851	C:97.2% [S:87.1% D:10.1%] F:0.8% , M:2.0%
B. napus	**Darmor-bzh**	**ONT**	**11,486,274 (24)**	**924 Mb**	**108,190**	**C:98.6% [S:7.0% D:91.6%] F:0.1% , M:1.3%**
B. oleracea	HDEM	ONT	9,491,203 (19)	555 Mb	61,279	C:96.2% [S:87.2% D:9.0%] F:0.7% , M:3.1%
B. rapa	Z1	ONT	5,519,976 (17)	402 Mb	46,721	C:96.7% [S:89.2% D:7.5%] F:0.6% , M:2.7%
B. oleracea	D134	PACBIO	3,591,417 (43)	530 Mb	43,868	C:95.1% [S:86.7% D:8.4%] F:0.7% , M:4.2%
B. napus	**Westar**	**PACBIO**	**3,130,520 (93)**	**1,008 Mb**	**100,194**	**C:98.3% [S:6.5% D:91.8%] F:0.3% , M:1.4%**
B. napus	**Express617**	**PACBIO**	**3,002,211 (78)**	**925 Mb**	**99,481**	**C:98.8% [S:7.2% D:91.6%] F:0.3% , M:0.9%**
B. napus	**Tapidor3**	**PACBIO**	**2,855,025 (104))**	**1,014 Mb**	**105,409**	**C:98.4% [S:8.1% D:90.3%] F:0.2% , M:1.4%**
B. napus	**Shengli3**	**PACBIO**	**2,825,656 (104)**	**1,002 Mb**	**103,920**	**C:98.1% [S:8.1% D:90.0%] F:0.5% , M:1.4%**
B. napus	**QuintaA**	**PACBIO**	**2,801,289 (104)**	**1,004 Mb**	**98,755**	**C:98.4% [S:6.6% D:91.8%] F:0.3% , M:1.3%**
B. napus	**No2127**	**PACBIO**	**2,704,645 (98)**	**1,012 Mb**	**105,894**	**C:98.0% [S:12.7% D:85.3%] F:0.5% , M:1.5%**
B. napus	**GanganF73**	**PACBIO**	**2,696,026 (98)**	**1,032 Mb**	**106,567**	**C:98.4% [S:8.7% D:89.7%] F:0.3% , M:1.3%**
B. napus	**Zheyou73**	**PACBIO**	**2,103,870 (126)**	**1,016 Mb**	**105,842**	**C:97.9% [S:8.1% D:89.8%] F:0.4% , M:1.7%**
B. napus	**Zs11**	**PACBIO**	**1,506,624 (186)**	**1,010 Mb**	**107,233**	**C:98.5% [S:8.7% D:89.8%] F:0.3% , M:1.2%**
B. rapa	Chiifu	PACBIO	1,386,548 (71)	353 Mb	60,609	C:99.6% [S:71.5% D:28.1%] F:0.1% , M:0.3%

Supplementary Table 10. Statistics of the *B. napus* long-reads assemblies (ordered by contigs N50 value). In the long-range technology column, “Comp” refers to comparative genomics meaning that contigs have been organized using synteny with an existing assembly at the chromosome-level .

	**genotype**	Long-read technology	Contig N50 (L50)	# gaps	Average gaps size (bp)	Long-range technology	Number of anchored bases	% of anchored bases
B. napus	darmor-bzh	ONT	11,486,274 (24)	268	96,984	Optical maps	867 Mb	93.8%
B. napus	westar	PACBIO	3,130,520 (93)	2,165	500	Comp	935 Mb	92.7%
B. napus	express617	PACBIO	3,002,211 (78)	661	1,195	Optical maps	765 Mb	82.7%
B. napus	tapidor3	PACBIO	2,855,025 (104))	1,771	500	Comp	921 Mb	90.8%
B. napus	shengli3	PACBIO	2,825,656 (104)	2,241	500	Comp	908 Mb	90.6%
B. napus	quintaA	PACBIO	2,801,289 (104)	2,132	500	Comp	920 Mb	91.6%
B. napus	no2127	PACBIO	2,704,645 (98)	2,074	500	Comp	910 Mb	89.9%
B. napus	ganganF73	PACBIO	2,696,026 (98)	1,976	500	Comp	922 Mb	89.1%
B. napus	zheyou73	PACBIO	2,103,870 (126)	2,428	500	Comp	907 Mb	89.2%
B. napus	zs11	PACBIO	1,506,624 (186)	5,460	500	Hi-C	961 Mb	95.0%

Supplementary Table 11. Statistics of direct RNA nanopore reads.

	**Raw reads**	Filtered reads	Error corrected reads (TALC)
Number of reads	10,416,515	9,099,437	8,523,238
Cumulative size (bp)	5,819,930,143	5,673,021,329	5,497,587,252
Average size (bp)	559	623	645
N50 (bp)	737	745	763
% of reads (>1Kb)	12.3%	13.9%	14.9%

Supplementary Table 12. Mapping of a sample of 1,000 reads using blat and est2genome.

	Filtered reads	Error corrected reads (TALC)
% of mapped reads	99.45%	99.78%
Identity percent	90.8%	97.80%
Number of exons per model	2.04	2.05
% of GT-AG splice sites	90.43%	96.51%

Supplementary Table 13. Repetitive content of the Darmor-bzh genome assembly compared to the regions present in Darmor-bzh but absent from zs11.

	Total length (Mb)	Masked proportion	LTR Copia	LTR Gypsy	DNA CMC-EnSpm	LINE	Satellite
darmor-bzh	924	53.86%	15.55%	13.05%	6.66%	6.37%	1.66%
zs11 gaps	19.2	**82.89%**	**25.29%**	13.69%	5.15%	**16.64%**	**19.22%**

Supplementary Figure 1. Kmer spectrum of *Brassica napus* Darmor-bzh computed using Genomescope which report an estimated genome size of 862Mb.

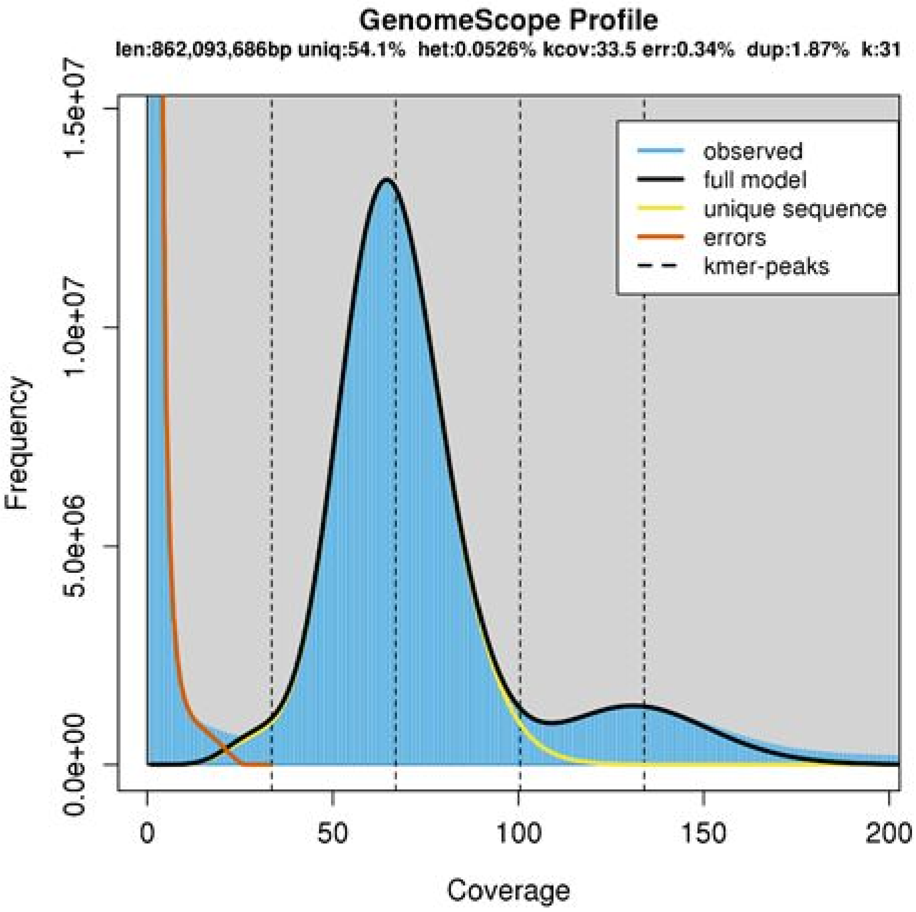

Supplementary Figure 2. KAT analysis of the *Brassica napus* Darmor-bzh assembly obtained using Flye. **A.** raw assembly. **B.** polished assembly.

A.

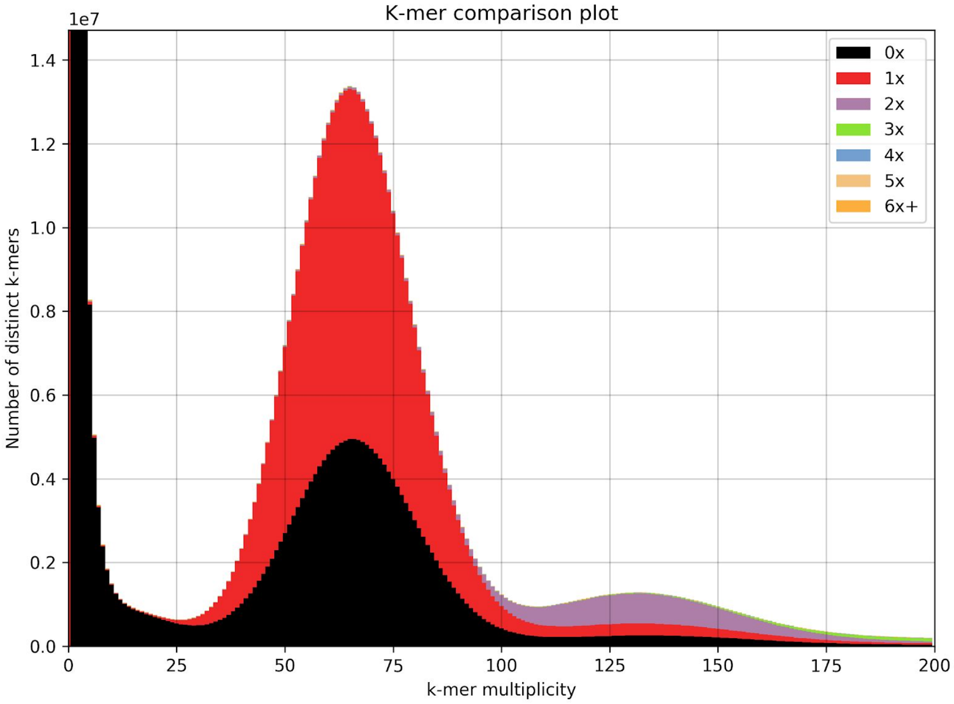

B.

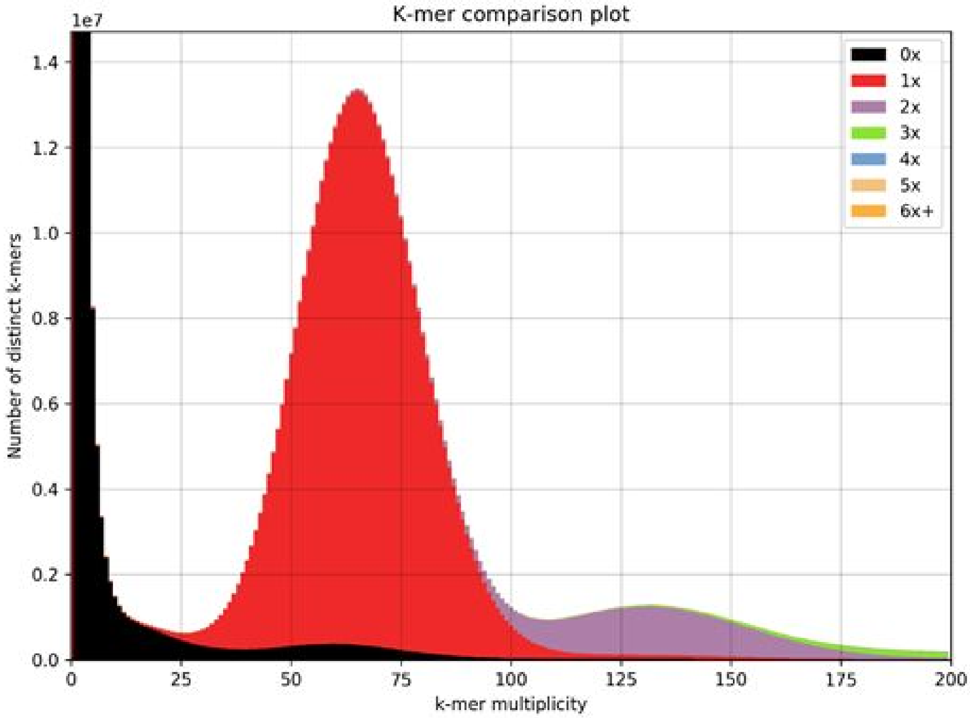

Supplementary Figure 3. Schematic diagram summarising the genome assembly, scaffolding and annotation steps.

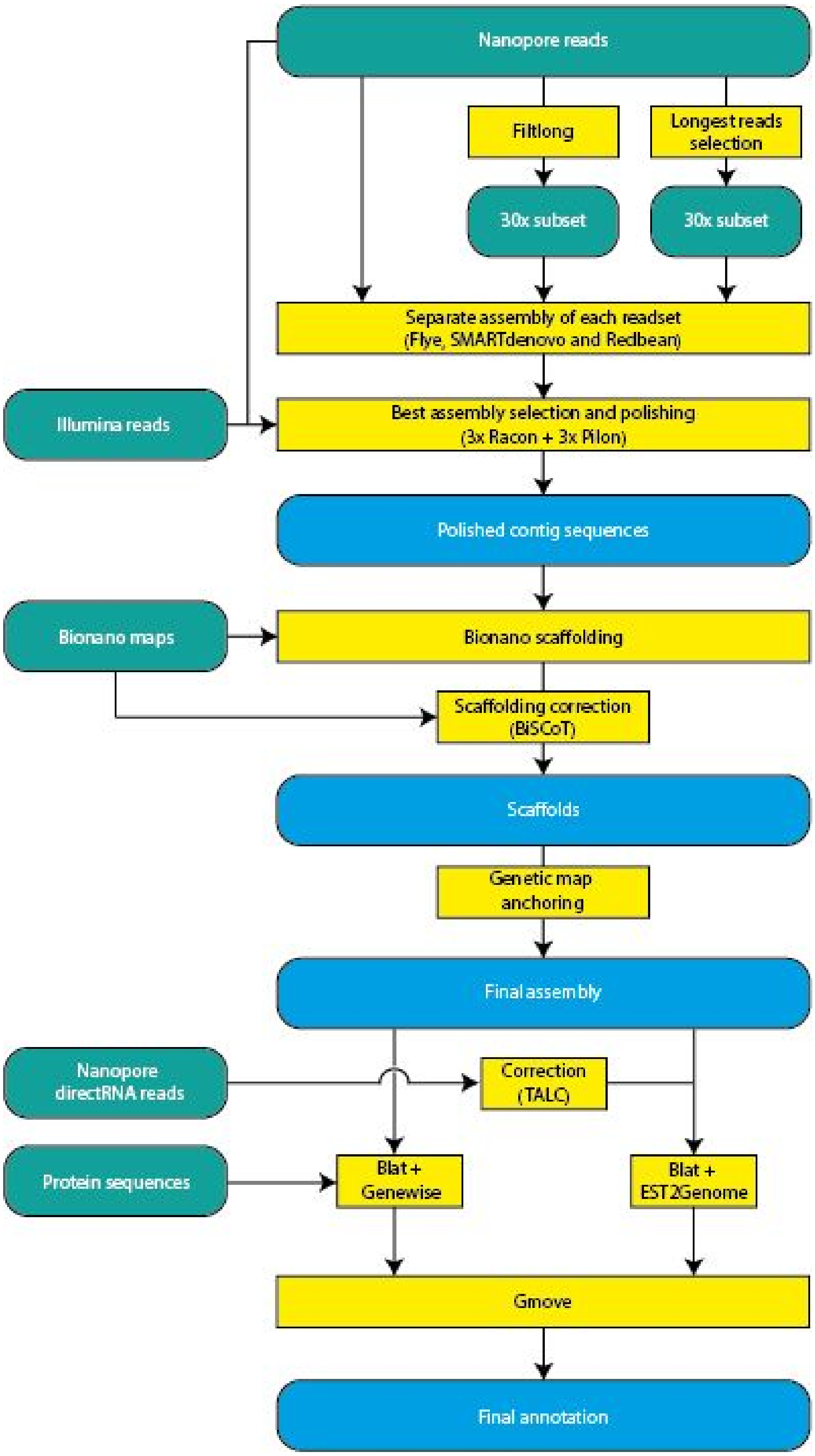

Supplementary Figure 4. Example of a difficult to detect transcript isoform with short-reads (orange track). Two nanopore long-reads (blue track) show a co-occurence of the presence of the second exon with the absence of the fourth exon.

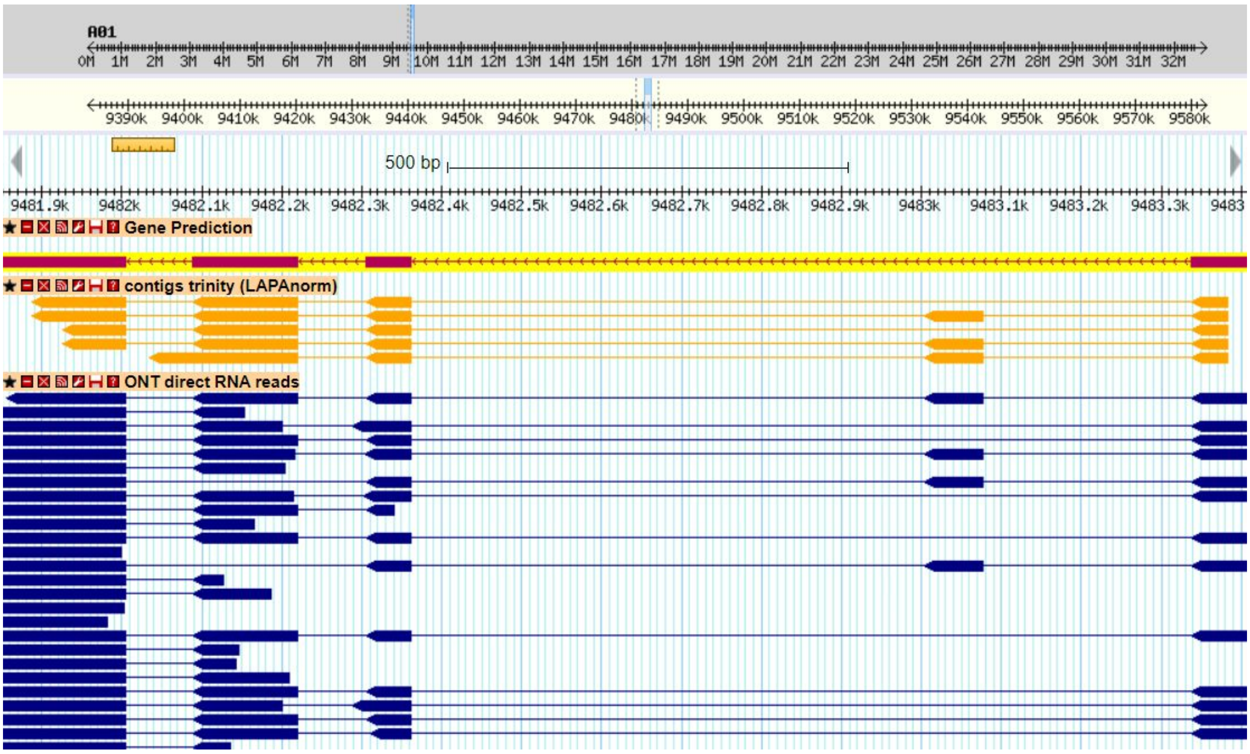

Supplement: giaa137_Supplemental_Files [file giaa137_supplemental_files.zip › BrassicaNapus_genome_SupData.pdf]
