# Supplementary material for: Long-read assembly of the Brassica napus reference genome Darmor-bzh
Source: Gigascience. 2020 Dec 15;9(12):giaa137. doi: 10.1093/gigascience/giaa137 (PMC7736779; doi:10.1093/gigascience/giaa137)
Supplement: giaa137_Response_to_Reviewer_Comments_Original_Submission [file giaa137_response_to_reviewer_comments_original_submission.pdf]

Author's Response To Reviewer Comments

Close

First of all, we would like to thank the three reviewers for their meticulous reading of the manuscript. We have taken into account their comments, and believe that this new version of the manuscript is now of better quality. During the first submission, we put the article on the BioRxiv server and received some feedback from the scientific community, especially regarding the correspondence between genes of the v5 and v10 assemblies. We have decided to provide a correspondence table with this manuscript (Table S18) and have described our method and results in a dedicated section of the manuscript.
Reviewer #1:
The authors described a new Brassica napus genome assembly and compared it to other Brassica genomes, particularly including the older version of the same genotype. The comparisons in terms of gaps, anchored markers, anchored BUSCOs, gene annotation and RGA not only show solid evidence of improvement, but also provide the Brassica community with important information to transit from the older assembly version.

The manuscript is well-written with logical flow of methods and conclusions. I particularly enjoyed the emphasis on detailed explanation of technical methods, including choice of parameters and filtering criteria, because they enable readers to easily decide whether the results make sense. It also shows that the analyses were done with careful thoughts and thorough optimization.

Below are some minor comments, as well as suggestions that may or may not be addressed by the authors:

1. How well do the Illumina reads (ERX397788 and ERX397800) align to the Darmor v10, since they originated from data of a few years ago? This information is not crucial because 1) they were not directly used for gene prediction, and 2) TALC-corrected reads mapped better, but could be useful to judge the correctness of TALC outcome.
We aligned the Illumina reads on the v5 and v10 genome assemblies and observed a higher proportion of aligned reads on the v10 genome: 89.15% vs 88.42% for ERX397788 and 89.62% vs 88.51% for ERX397800. The difference is slight but in favor of the v10 assembly.

2. Line "These misassembled regions were validated using the new ZS11 assembly as a control (Figure 2, highlighted in purple on the C07 chromosome)": it looks like green instead?
We replaced purple with green in the text.

3. Figure 2: positions where pericentromeric-flanking markers aligned could be indicated, to further highlight the additionally assembled/inversed regions.
We have added in Figure 2 the alignment positions of pericentromeric-flanking markers.

4. Are alternatively spliced transcripts provided? File "BnapusDarmor-bzh_annotation.gff" only contains primary transcripts.
The alternative transcripts were not predicted, at each loci a single transcript was annotated. However, we have now added requested files that contain alternative splicing events (intron retention and exon skipping) in the GigaDB repository.

5. Table S14 and S15: column names are missing
Column names of Tables S14 and S15 have been added.

6. Table S1: accession numbers are missing (probably pending, just a reminder)
Accession numbers have now been added to Table S1.

Reviewer #2:
In this Data Note, Rousseau-Gueutin et al. describe a new genome assembly and annotation of the allotetraploid Brassica napus Darmor-bzh. The assembly is based on Nanopore sequencing combined with an Bionano optical map. Previous assemblies are based on older technologies and issues with

assembly and annotation quality make an upgraded reference genome and annotation a welcome and valuable resource for the Brassica research community.

I do have a number of comments, however, my copy of the manuscript does not have line numbers so I will refer to page numbers in the text.

Comments:
Pg 3 "Background" - the manuscript starts off with a well written background on the relationships of the Brassica genomes and a review of the genome sequencing of Brassica genomes, and status and issues of the B. napus Darmor-bzh v5 and v8 assemblies.
Thanks for your positive feedback.

Pg 5 "These trimming and removal steps were achieved using in-house-designed software based on the FastX package" - since the software is open source and in Github, just use its name (fastxtend) and parameters and version or commit id used for the analysis
We have changed the text accordingly and replaced "... were achieved using in-house-designed software based on the FastX package" with "... were achieved using Fastxtend tools".

Pg 6 "A taxonomical assignation was performed using Centrifuge [16] for each dataset to detect potential contaminations." - needs more information (version, parameters, database) and report the results from the centrifuge run.
We added the centrifuge version, parameters and database information, as well as an overview of the result in the corresponding section : "On each run, approximately 65% of the reads were unassigned and almost 35% were assigned to the Brassica genus. In addition, we detected a small proportion (<0.6%) of reads corresponding to various metazoa."

Pg 7 "In order to get the best possible genome assembly" -
I found this paragraph odd, especially in the context of a data note. There is no rationale for why these three assemblers were chosen other than "to get the best possible genome assembly" and of course, I could immediately think of another 6 assemblers you could have tried for achieve this goal. Flye is a fine choice, the results are excellent, so I would recommend modifying this paragraph and modifying the table to just report the assembly of the final contigs with Flye
We agree with the reviewer's comment regarding the formulation "to get the best possible genome assembly". We changed the sentence and replaced it with "We used three different assemblers…". However, we have decided to keep the results of the three assemblers in supplementary data as we believe it may be useful for readers and especially for people working on genome assembly.

Pg7 "As nanopore reads contain systematic error" - I'm surprised that the assembly was not also polished with a tool that specializes in polishing nanopore assemblies such as Nanopolish or Medaka, especially as Nanopolish was used for the calling of 5mC bases from the raw WGS reads.
By the time we did the assembly years ago, nanopolish was too slow to work on large genomes and medaka was at an early stage of development. That's why we decided to use classic polishing tools like Racon and Pilon.

Pg 9 "Detection of modified bases" - This section showcases the calling from 5mCs from raw nanopore reads, however the results seem only to summarized as a track on Figure 1. This is fine, but the raw data for the track generated with what I assume is nanopolish call-methylation and the data filtered as described should be included in the data repository accompanying the paper
We added two files to the GigaDB repository. The first is the raw file, a tabular file with eleven columns that contain the nanopolish results for each nanopore read. The second is a tabular file with four columns used for generating the graph in Figure 1.

"We analysed the electric signal to detect the" - odd phrasing, rewrite to state the method used
We agree with the reviewer and decided to delete this sentence which was not informative.

Pg 10 - Gene Prediction - the gene annotation utilized a range of protein evidence along with the direct RNA reads aligned to the genome (using some venerable "old school" aligners!). Gene models were created using gmove, a tool I was not familiar with, but appears to be similar in function to EVM or MAKER to generate a consensus gene model. I'd like the authors to comment on why a de novo approach to annotation was not taken and why the direct RNA sequencing was not leveraged to predict isoforms.
Indeed, genewise and est2genome are "old school" aligners, but we agree that they are still very

efficient in terms of alignment quality. We decided not to use a de novo approach, as the number of biological resources was high and specific genes could have been annotated using RNA-Seq from the same genotype. We did not predict the isoforms because we were concerned that the corrected nanopore reads might contain unreal isoforms. We believe that isoform prediction using long noisy reads is not mature enough, but this will undoubtedly be an important point to exploit while relying on future methods.

Pg 11 - "Brassica (CentBr1, CentBr2, CRB, PCRBr, TR238 and TR805) which were blasted" - Changed 'blasted' to 'searched'
We have modified the text accordingly.

Pg 12 - Comparison with existing assemblies and annotations
Throughout the manuscript, BUSCO results are not reported using the summary string recommended by the BUSCO developers. Also, the short_summary.*.txt BUSCO results file for the genome and annotation is missing from the data repo. As B. napus is an allotetraploid, I would like to see how many BUSCOs are complete and duplicated vs single.
This is a good point, we have added the full summary string in Table 1 for the three genomes of Darmor-bzh. However, since Table 3 is already dense, we prefered to create a new table in the supplementary Information for these additional results (Table S9). In addition, we have added all the BUSCO short_summary.*.txt in the GigaDB repository.

Pg 15 Alternative splicing events - The data describing the IR and skipped exon events in the results is not present in the data repo and should be added.
We have added requested files that contain alternative splicing events (intron retention and exon skipping) in the GigaDB repository.

General comment - Gigascience has set a high bar for reporting software tool versions and command line parameters to promote reproducibility. This information is missing throughout the manuscript and needs to be added. Data referred to in the results should also be added to the data repo accompanying the paper.
We have tried to be exhaustive, and we have added the version and parameters for each software we have used. In addition, the GigaDB repository now contains all the results generated for this publication.

Reviewer #3:
The authors present an improved version of the Darmor-bzh (Brassica napus) reference genome and annotation using a combination of long-reads sequencing data, optical and genetic maps. They report a detailed comparison of the new Darmor-bzh v10 genome assembly and annotation with previous releases of the same genome (evaluating contiguity, gene and repeat content, Resistance Genes Analogs) as well as with the other available Brassica assemblies. While they provide a valuable resource for the plant community, I have several major and minor comments.

Major comments
1) The assembly size of Darmor-bzh v10 reported in this paper (924 Mb) is larger than the previous releases v5 and v8 (850 Mb). Since the real size of the genome is unknown, it would be interesting to provide a genome size estimation based on k-mer analysis of Illumina reads or flow cytometry. The authors did not report the genome size parameter used to assemble the reads with Redbean and Flye. In Supplementary Table 1, they computed the coverage based on an estimated genome size of 1.2 Gb but it is unclear why they chose this value.
We chose the value of 1.2Gb because the genome size was first estimated at 1,132 Mb by Johnston et al. (https://doi.org/10.1093/aob/mci016) using flow cytometry. However, the k-mer spectrum (k=31) provides a lower estimate of 862Mb. We have added a paragraph that gives information on genome size estimation in the “Genome assembly” section.

2) Could you please provide more details about the criteria used to select the 'best' assembly? On page 7, the authors report that: 'Then, we selected the 'best' assembly based not only on contiguity metrics such as N50 but also cumulative size. The Flye assembler using the longest reads produced the most contiguous assembly and lead to a contig N50 of 10.0Mb.' My understanding is that the Flye assembly using the longest reads (ie only 30X instead of the 93X of data generated) was selected as the 'best' assembly because of its higher contig N50 and maybe also because of its cumulative size? (if yes, what criteria were used for the cumulative size?). Is that correct?

Yes you are right, the Flye (longest reads) and smartdenovo (all reads) assemblies were very similar in terms of contiguity (N50 and N90), but even though the N50 was slightly better using smartdenovo, we decided to keep the Flye assembly as its cumulative size was higher. We have now changed the text to explain our choice.

- The criteria to decide on a best assembly could include other metrics such as gene content and k-mer spectra. Have you estimated the BUSCO completeness for the different assemblers presented in Supplementary Table 2? A comparison of the k-mer spectrum of the assembly to the k-mer spectrum of the Illumina reads could be presented as well.
In general, the BUSCO score is a good metric for comparing assemblies, but in the case of unpolished assemblies the results are difficult to interpret. We added the BUSCO scores in Table S2, and the completeness scores were between 28.3% and 44.3% for wtdbg2, 54.2% and 90.9% for smartdenovo and 76.7% and 78.7% for Flye. In this case, the metrics reflect the accuracy of the polishing step of each assembler. For the same reason, the KAT plot is biased due to the consensus accuracy of each assembler. We have added the KAT plot for the raw and polished Flye assemblies in Figure S2.

- With Smartdenovo, adding more coverage resulted in a larger assembly with a higher contig N50. The authors reported in Supplementary Table 2 that the Flye assembly using all the reads "crashed". It would be interesting to see the Flye assembly results using 50 or 60X coverage, if the full dataset cannot be assembled. The Flye parameter --asm-coverage could be used to reduce the memory requirements of the program during the initial contig extension stage.
Indeed, the input dataset can be sampled in different ways, but generating numerous assemblies is time consuming. When we started the project we decided to generate three subsets of reads and use three different assemblers. Out of curiosity we ran the flye assembler with 50X of ONT reads, and got an assembly with a contig N50 of 9.4Mb (31 contigs), a contig N90 of 321Kb (209 contigs) and a cumulative size of 963Mb. So even though the cumulative size is higher, the N90 is twice as low as the selected assembly.

3) Long read sequencing technologies are a powerful tool to study alternative splicing and this is an area of active development. The 'Alternative splicing events' section could include additional analysis or discussion.
We first generated the direct RNA dataset to improve gene prediction and not to examine splicing events although this type of data opens up new perspectives in this area. Here we have pooled RNAs from two different tissues (leaf and root) and have not generated replicates which limits the applicative interest of this dataset.

- Have you tried to assess the confidence in the splicing events detected using the ONT long reads? It could be interested to try an alternative method or to include a comparison with the splicing events identified with the Illumina short reads.
To date, there is no mature software available that deals with ONT reads to detect splicing events. However, we have used the illumina dataset and run the same type of analysis to compare the intron retention detected by the two technologies. We find a good overlap between the two methods by allowing a low coverage of Illumina reads (>5). We have modified the corresponding section.

- The reported intron retention percentage in Darmor-Bzh v10 (16%) is much smaller than what was detected in Darmor-Bzh v5 (62%, Chalhoub et al., 2014). Intron retention detection is challenging but this could be investigated or discussed.
In Chalhoub et al., 62% refers to the proportion of IR events among the gene having splicing events (48%), showing that IR events are in the majority. The correct numbers are 29% and 16%, and the smaller difference can be explained by the lower sequencing depth of the nanopore dataset. We have added the points described above in the corresponding section.

- Could you provide the list of splicing events as a supplementary table?
We have added requested files that contain alternative splicing events (intron retention and exon skipping) in the GigaDB repository.

Minor comments
- It would be helpful to provide a schematic diagram summarising the genome assembly, scaffolding and annotation steps and including all the datasets and tools used to obtain the final v10 assembly.
We have added as a Supplementary Figure (Figure S3), a schematic diagram describing the different steps for obtaining the final assembly and the gene prediction.

- p3: The GBS abbreviation should be explained.
We have modified the text accordingly.

- p5-6: Nanopore sequencing sections. Which flow cell type was used?
For genomic and RNA sequencing, we used R9.4.1 flowcells. This information has been added to the nanopore sequencing sections.

- p6: Which basecalling tool was used ? Please also indicate the version and parameters used.
Genomic reads were basecalled using the guppy basecaller (version 1.4.3) with the 'fast' configuration and default parameters. RNA-Seq reads were basecalled using the guppy basecaller (version 3.2.8) with the 'hac' configuration and default parameters. We have added the missing information in the corresponding sections.

- p7: Long reads genome assembly section. Which version of each assembler and polishing tool were used?
We have added the assemblers' version in the corresponding section.

- p7: Is there a reason for choosing to perform three iterations of Racon and three iterations of Pilon? For the selected assembly, it would be interesting to report the assembly metrics after each round of polishing including genome completeness estimation.
This is an interesting remark, we have followed the observation reported on the first plant genome assembled with nanopore data (https://dx.doi.org/10.1105%2Ftpc.17.00521). This study recommended performing three iterations of Racon, followed by three iterations of Pilon. We ran the BUSCO analysis on each intermediate assembly to look at the gain of each iteration. We have now added these results to Table S3.

- p8: "As already reported, we found in several cases that the nanopore contigs were overlapping (based on the optical map)". Please include a citation of where this has been already reported.
We now cite the following article : https://doi.org/10.1038/s41477-018-0289-4.

- p8-10: Please also include the versions used for the tools mentioned ie nucmer, TALC etc.
We have added the software's versions in the corresponding section.

- p8-9: Large Misassembled Inverted regions section: Is the script to identify the LMI provided?
We have added the script (get_LMIs.sh) to the GigaDB repository.

- p13: "(Figure 2, highlighted in purple on the C07 chromosome)". I cant see any purple in Fig2 in the C07 chromosome plot, do you mean green?
We replaced purple with green in the text.

- p13: 'the proportion of anchored sequence (from 765 Mb for Express617 to 961 Mb for Zs11)'. The term proportion is mentioned but the reported numbers correspond to sizes.
We changed the text, and replaced 'the proportion of anchored bases' with 'the number of anchored bases'.

- p13: "The eleven Brassica genomes sequenced using PACBIO have a contig N50 between 1.4Mb and 3.6Mb whereas all the ONT assemblies have a contig N50 higher than 5.5Mb." I would replace "all the ONT" by "the four ONT" assemblies.
We replaced "all the ONT" with "the four ONT" assemblies.

- p14: A word is missing in the sentence "As a comparison, we found 5,460 in the PACBIO assembly of the Zs11 genotype."
We have added the missing word "gaps".

- p14, p16. Interestingly, the authors reported an example of a large transposable element resolved thanks to ONT ultra long reads (as compared to a gap in the PacBio+HiC assembly). They also assembled a filtered subset of reads ('Filtlong reads' including 2X >100 kb). Have you tried to assemble the ONT data without including the ultra long reads at all (ie 6X >100 kb)? It would be interesting to see the impact of those ultra-long reads on the overall assembly quality, especially because it could be challenging to generate ultra long reads and this is a specificity of the ONT technology.
As recommended we ran an assembly using Flye but without the ultra-long reads, and got an 894Mb assembly composed of 4,590 contigs, with a contig N50 of 9.3Mb and N90 of 349Kb. The N50 is still

very high, but the assembly contains almost 3 times the number of contigs of the assembly obtained with ultra-long reads (4,590 vs 1,594). The assembly at the scaffold level will necessarily contain a higher number of gaps indicating the importance of long-reads.

- page 15 "From the genomic alignments of raw reads, with an identity percent higher than 90%". Which strategy was used to align the reads ?
Alignments were performed with Est2Genome, we have added the following description in the corresponding section : "Raw reads were aligned on the genome in a two-steps strategy. First, BLAT (version 36 with default parameters) was used to fastly localize corresponding putative regions of these RNA reads on the genome. The best match for each read was selected and a second alignment was performed using Est2Genome (version 5.2 with default parameters)."

Tables
- p26-27. Tables 1 and 2. I would add a column in those tables to include the reference paper and the corresponding release date for each assembly.
Table 1 and 2 are already dense, we preferred to create a new table that includes global information like the reference paper and the release date.

Supplementary Tables
- Supplementary Table 1. The accession numbers are missing.
Accession numbers have now been added to Table S1.

- Supplementary Table 2:
-The authors reported that the Flye assembly using all the reads "crashed". Was it a memory issue due to the computing resources used?
No it was not a memory issue. In our experience, the earlier versions of Flye were not very stable and assemblies often crashed for no easily identifiable reasons. Flye is now much more stable and crashes are very rare thanks to the many updates that its authors have made.

-I would add the BUSCO completeness estimation for each of those assemblies.
Generally the BUSCO score is a great metric to compare assemblies, but in the case of unpolished assemblies the results are difficult to interpret. We added the BUSCO scores in Table S2, and the completeness scores were between 28.3% and 44.3% for wtdbg2, 54.2% and 90.9% for smartdenovo and 76.7% and 78.7% for Flye. In this case, the metrics reflect the accuracy of the polishing step of each assembler.

- Supplementary Table 8. Could you please add in the legend that 'Comp' refers to Comparative genomics (Long-range technology column)?
We added the following sentence in the legend : " In the long-range technology column, "Comp" refers to comparative genomics meaning that contigs have been organized using synteny with an existing assembly at the chromosome-level ."

Close
