# Supplementary material for: Long-read assembly of the Brassica napus reference genome Darmor-bzh
Source: Gigascience. 2020 Dec 15;9(12):giaa137. doi: 10.1093/gigascience/giaa137 (PMC7736779; doi:10.1093/gigascience/giaa137)
Supplement: giaa137_Response_to_Reviewer_Comments_Revision_1 [file giaa137_response_to_reviewer_comments_revision_1.pdf]

Author's Response To Reviewer Comments

Close

Please find a revised version of our manuscript that meets editorial requests.

Close
