# Supplementary material for: Long-read assembly of the Brassica napus reference genome Darmor-bzh
Source: Gigascience. 2020 Dec 15;9(12):giaa137. doi: 10.1093/gigascience/giaa137 (PMC7736779; doi:10.1093/gigascience/giaa137)
Supplement: giaa137_Reviewer_1_Report_Original_Submission — Huey Tyng Lee -- 8/24/2020 Reviewed [file giaa137_reviewer_1_report_original_submission.pdf]

Reviewer Report

Title: Long-reads assembly of the Brassica napus reference genome, Darmor-bzh

Version: Original Submission **Date:** 8/24/2020

Reviewer name: Huey Tyng Lee

Reviewer Comments to Author:

The authors described a new Brassica napus genome assembly and compared it to other Brassica genomes, particularly including the older version of the same genotype. The comparisons in terms of gaps, anchored markers, anchored BUSCOs, gene annotation and RGA not only show solid evidence of improvement, but also provide the Brassica community with important information to transit from the older assembly version.

The manuscript is well-written with logical flow of methods and conclusions. I particularly enjoyed the emphasis on detailed explanation of technical methods, including choice of parameters and filtering criteria, because they enable readers to easily decide whether the results make sense. It also shows that the analyses were done with careful thoughts and thorough optimization.

Below are some minor comments, as well as suggestions that may or may not be addressed by the authors:

1. How well do the Illumina reads (ERX397788 and ERX397800) align to the Darmor v10, since they originated from data of a few years ago? This information is not crucial because 1) they were not directly used for gene prediction, and 2) TALC-corrected reads mapped better, but could be useful to judge the correctness of TALC outcome.
2. Line "These misassembled regions were validated using the new ZS11 assembly as a control (Figure 2, highlighted in purple on the C07 chromosome)": it looks like green instead?
3. Figure 2: positions where pericentromeric-flanking markers aligned could be indicated, to further highlight the additionally assembled/inversed regions.
4. Are alternatively spliced transcripts provided? File "BnapusDarmor-bzh_annotation.gff" only contains primary transcripts.
5. Table S14 and S15: column names are missing
6. Table S1: accession numbers are missing (probably pending, just a reminder)

Level of Interest

Please indicate how interesting you found the manuscript: Choose an item.

Quality of Written English

Please indicate the quality of language in the manuscript: Choose an item.

Declaration of Competing Interests

Please complete a declaration of competing interests, considering the following questions:

- Have you in the past five years received reimbursements, fees, funding, or salary from an organisation that may in any way gain or lose financially from the publication of this manuscript, either now or in the future?
- Do you hold any stocks or shares in an organisation that may in any way gain or lose financially from the publication of this manuscript, either now or in the future?
- Do you hold or are you currently applying for any patents relating to the content of the manuscript?
- Have you received reimbursements, fees, funding, or salary from an organization that holds or has applied for patents relating to the content of the manuscript?
- Do you have any other financial competing interests?
- Do you have any non-financial competing interests in relation to this paper?

If you can answer no to all of the above, write 'I declare that I have no competing interests' below. If your reply is yes to any, please give details below.

I declare that I have no competing interests.

I agree to the open peer review policy of the journal. I understand that my name will be included on my report to the authors and, if the manuscript is accepted for publication, my named report including any attachments I upload will be posted on the website along with the authors' responses. I agree for my report to be made available under an Open Access Creative Commons CC-BY license (http://creativecommons.org/licenses/by/4.0/). I understand that any comments which I do not wish to be included in my named report can be included as confidential comments to the editors, which will not be published.

Choose an item.

To further support our reviewers, we have joined with Publons, where you can gain additional credit to further highlight your hard work (see: https://publons.com/journal/530/gigascience). On publication of this paper, your review will be automatically added to Publons, you can then choose whether or not to claim your Publons credit. I understand this statement.

Yes Choose an item.
