# Supplementary material for: Long-read assembly of the Brassica napus reference genome Darmor-bzh
Source: Gigascience. 2020 Dec 15;9(12):giaa137. doi: 10.1093/gigascience/giaa137 (PMC7736779; doi:10.1093/gigascience/giaa137)
Supplement: giaa137_Reviewer_2_Report_Original_Submission — John Hamilton -- 8/24/2020 Reviewed [file giaa137_reviewer_2_report_original_submission.pdf]

Reviewer Report

Title: Long-reads assembly of the Brassica napus reference genome, Darmor-bzh

Version: Original Submission **Date:** 8/24/2020

Reviewer name: John Hamilton

Reviewer Comments to Author:

In this Data Note, Rousseau-Gueutin et al. describe a new genome assembly and annotation of the allotetraploid Brassica napus Darmor-bzh. The assembly is based on Nanopore sequencing combined with an Bionano optical map. Previous assemblies are based on older technologies and issues with assembly and annotation quality make an upgraded reference genome and annotation a welcome and valuable resource for the Brassica research community.

I do have a number of comments, however, my copy of the manuscript does not have line numbers so I will refer to page numbers in the text.

Comments:

Pg 3 "Background" - the manuscript starts off with a well written background on the relationships of the Brassica genomes and a review of the genome sequencing of Brassica genomes, and status and issues of the B. napus Darmor-bzh v5 and v8 assemblies.

Pg 5 "These trimming and removal steps were achieved using in-house-designed software based on the FastX package" - since the software is open source and in Github, just use its name (fastxtend) and parameters and version or commit id used for the analysis

Pg 6 "A taxonomical assignation was performed using Centrifuge [16] for each dataset to detect potential contaminations." - needs more information (version, parameters, database) and report the results from the centrifuge run.

Pg 7 "In order to get the best possible genome assembly" -

I found this paragraph odd, especially in the context of a data note. There is no rationale for why these three assemblers were chosen other than "to get the best possible genome assembly" and of course, I could immediately think of another 6 assemblers you could have tried for achieve this goal.

Flye is a fine choice, the results are excellent, so I would recommend modifying this paragraph and modifying the table to just report the assembly of the final contigs with Flye

Pg7 "As nanopore reads contain systematic error" - I'm surprised that the assembly was not also polished with a tool that specializes in polishing nanopore assemblies such as Nanopolish or Medaka, especially as Nanopolish was used for the calling of 5mC bases from the raw WGS reads.

Pg 9 "Detection of modified bases" - This section showcases the calling from 5mCs from raw nanopore reads, however the results seem only to summarized as a track on Figure 1. This is fine, but the raw data for the track generated with what I assume is nanopolish call-methylation and the data filtered as described should be included in the data repository accompanying the paper

"We analysed the electric signal to detect the" - odd phrasing, rewrite to state the method used

Pg 10 - Gene Prediction - the gene annotation utilized a range of protein evidence along with the direct RNA reads aligned to the genome (using some venerable "old school" aligners!). Gene models were

created using gmove, a tool I was not familiar with, but appears to be similar in function to EVM or MAKER to generate a consensus gene model. I'd like the authors to comment on why a de novo approach to annotation was not taken and why the direct RNA sequencing was not leveraged to predict isoforms.
Pg 11 - "Brassica (CentBr1, CentBr2, CRB, PCRBr, TR238 and TR805) which were blasted" -
Changed 'blasted' to 'searched'
Pg 12 - Comparison with existing assemblies and annotations
Throughout the manuscript, BUSCO results are not reported using the summary string recommended by the BUSCO developers. Also, the short_summary.*.txt BUSCO results file for the genome and annotation is missing from the data repo. As B. napus is an allotetraploid, I would like to see how many BUSCOs are complete and duplicated vs single.
Pg 15 Alternative splicing events - The data describing the IR and skipped exon events in the results is not present in the data repo and should be added.
General comment - Gigascience has set a high bar for reporting software tool versions and command line parameters to promote reproducibility. This information is missing throughout the manuscript and needs to be added. Data referred to in the results should also be added to the data repo accompanying the paper.

Level of Interest

Please indicate how interesting you found the manuscript: Choose an item.

Quality of Written English

Please indicate the quality of language in the manuscript: Choose an item.

Declaration of Competing Interests

Please complete a declaration of competing interests, considering the following questions:

- Have you in the past five years received reimbursements, fees, funding, or salary from an organisation that may in any way gain or lose financially from the publication of this manuscript, either now or in the future?
- Do you hold any stocks or shares in an organisation that may in any way gain or lose financially from the publication of this manuscript, either now or in the future?
- Do you hold or are you currently applying for any patents relating to the content of the manuscript?
- Have you received reimbursements, fees, funding, or salary from an organization that holds or has applied for patents relating to the content of the manuscript?
- Do you have any other financial competing interests?
- Do you have any non-financial competing interests in relation to this paper?

If you can answer no to all of the above, write 'I declare that I have no competing interests' below. If your reply is yes to any, please give details below.

I declare that I have no competing interests

I agree to the open peer review policy of the journal. I understand that my name will be included on my report to the authors and, if the manuscript is accepted for publication, my named report including any attachments I upload will be posted on the website along with the authors' responses. I agree for my report to be made available under an Open Access Creative Commons CC-BY license (http://creativecommons.org/licenses/by/4.0/). I understand that any comments which I do not wish to be included in my named report can be included as confidential comments to the editors, which will not be published.

Choose an item.

To further support our reviewers, we have joined with Publons, where you can gain additional credit to further highlight your hard work (see: https://publons.com/journal/530/gigascience). On publication of this paper, your review will be automatically added to Publons, you can then choose whether or not to claim your Publons credit. I understand this statement.

Yes Choose an item.
