# Supplementary material for: Long-read assembly of the Brassica napus reference genome Darmor-bzh
Source: Gigascience. 2020 Dec 15;9(12):giaa137. doi: 10.1093/gigascience/giaa137 (PMC7736779; doi:10.1093/gigascience/giaa137)
Supplement: giaa137_Reviewer_3_Report_Original_Submission — Valentine Murigneux, M.Sc. -- 9/1/2020 Reviewed [file giaa137_reviewer_3_report_original_submission.pdf]

Reviewer Report

Title: Long-reads assembly of the Brassica napus reference genome, Darmor-bzh

Version: Original Submission **Date:** 9/1/2020

Reviewer name: Valentine Murigneux, M.Sc.

Reviewer Comments to Author:

The authors present an improved version of the Darmor-bzh (Brassica napus) reference genome and annotation using a combination of long-reads sequencing data, optical and genetic maps. They report a detailed comparison of the new Darmor-bzh v10 genome assembly and annotation with previous releases of the same genome (evaluating contiguity, gene and repeat content, Resistance Genes Analogs) as well as with the other available Brassica assemblies. While they provide a valuable resource for the plant community, I have several major and minor comments.
Major comments
1) The assembly size of Darmor-bzh v10 reported in this paper (924 Mb) is larger than the previous releases v5 and v8 (850 Mb). Since the real size of the genome is unknown, it would be interesting to provide a genome size estimation based on k-mer analysis of Illumina reads or flow cytometry. The authors did not report the genome size parameter used to assemble the reads with Redbean and Flye. In Supplementary Table 1, they computed the coverage based on an estimated genome size of 1.2 Gb but it is unclear why they chose this value.
2) Could you please provide more details about the criteria used to select the 'best' assembly? On page 7, the authors report that: 'Then, we selected the 'best' assembly based not only on contiguity metrics such as N50 but also cumulative size. The Flye assembler using the longest reads produced the most contiguous assembly and lead to a contig N50 of 10.0Mb.' My understanding is that the Flye assembly using the longest reads (ie only 30X instead of the 93X of data generated) was selected as the 'best' assembly because of its higher contig N50 and maybe also because of its cumulative size? (if yes, what criteria were used for the cumulative size?). Is that correct?
- The criteria to decide on a best assembly could include other metrics such as gene content and k-mer spectra. Have you estimated the BUSCO completeness for the different assemblers presented in Supplementary Table 2? A comparison of the k-mer spectrum of the assembly to the k-mer spectrum of the Illumina reads could be presented as well.
- With Smartdenovo, adding more coverage resulted in a larger assembly with a higher contig N50. The authors reported in Supplementary Table 2 that the Flye assembly using all the reads "crashed". It would be interesting to see the Flye assembly results using 50 or 60X coverage, if the full dataset cannot be assembled. The Flye parameter --asm-coverage could be used to reduce the memory requirements of the program during the initial contig extension stage.
3) Long read sequencing technologies are a powerful tool to study alternative splicing and this is an area of active development. The 'Alternative splicing events' section could include additional analysis or discussion.
- Have you tried to assess the confidence in the splicing events detected using the ONT long reads? It

could be interested to try an alternative method or to include a comparison with the splicing events identified with the Illumina short reads.
- The reported intron retention percentage in Darmor-Bzh v10 (16%) is much smaller than what was detected in Darmor-Bzh v5 (62%, Chalhoub et al., 2014). Intron retention detection is challenging but this could be investigated or discussed.
- Could you provide the list of splicing events as a supplementary table?

Minor comments

- It would be helpful to provide a schematic diagram summarising the genome assembly, scaffolding and annotation steps and including all the datasets and tools used to obtain the final v10 assembly.
- p3: The GBS abbreviation should be explained.
- p5-6: Nanopore sequencing sections. Which flow cell type was used?
- p6: Which basecalling tool was used ? Please also indicate the version and parameters used.
- p7: Long reads genome assembly section. Which version of each assembler and polishing tool were used?
- p7: Is there a reason for choosing to perform three iterations of Racon and three iterations of Pilon? For the selected assembly, it would be interesting to report the assembly metrics after each round of polishing including genome completeness estimation.
- p8: "As already reported, we found in several cases that the nanopore contigs were overlapping (based on the optical map)". Please include a citation of where this has been already reported.
- p8-10: Please also include the versions used for the tools mentioned ie nucmer, TALC etc.
- p8-9: Large Misassembled Inverted regions section: Is the script to identify the LMI provided?
- p13: "(Figure 2, highlighted in purple on the C07 chromosome)". I cant see any purple in Fig2 in the C07 chromosome plot, do you mean green?
- p13: 'the proportion of anchored sequence (from 765 Mb for Express617 to 961 Mb for Zs11)'. The term proportion is mentioned but the reported numbers correspond to sizes.
- p13: "The eleven Brassica genomes sequenced using PACBIO have a contig N50 between 1.4Mb and 3.6Mb whereas all the ONT assemblies have a contig N50 higher than 5.5Mb." I would replace "all the ONT" by "the four ONT" assemblies.
- p14: A word is missing in the sentence "As a comparison, we found 5,460 in the PACBIO assembly of the Zs11 genotype."
- p14, p16. Interestingly, the authors reported an example of a large transposable element resolved thanks to ONT ultra long reads (as compared to a gap in the PacBio+HiC assembly). They also assembled a filtered subset of reads ('Filtlong reads' including 2X >100 kb). Have you tried to assemble the ONT data without including the ultra long reads at all (ie 6X >100 kb)? It would be interesting to see the impact of those ultra-long reads on the overall assembly quality, especially because it could be challenging to generate ultra long reads and this is a specificity of the ONT technology.
- page 15 "From the genomic alignments of raw reads, with an identity percent higher than 90%". Which strategy was used to align the reads ?

Tables

- p26-27. Tables 1 and 2. I would add a column in those tables to include the reference paper and the corresponding release date for each assembly.

Supplementary Tables

- Supplementary Table 1. The accession numbers are missing.
- Supplementary Table 2:
-The authors reported that the Flye assembly using all the reads "crashed". Was it a memory issue due to the computing resources used?
-I would add the BUSCO completeness estimation for each of those assemblies.
- Supplementary Table 8. Could you please add in the legend that 'Comp' refers to Comparative genomics (Long-range technology column)?

Level of Interest

Please indicate how interesting you found the manuscript: Choose an item.

Quality of Written English

Please indicate the quality of language in the manuscript: Choose an item.

Declaration of Competing Interests

Please complete a declaration of competing interests, considering the following questions:

- Have you in the past five years received reimbursements, fees, funding, or salary from an organisation that may in any way gain or lose financially from the publication of this manuscript, either now or in the future?
- Do you hold any stocks or shares in an organisation that may in any way gain or lose financially from the publication of this manuscript, either now or in the future?
- Do you hold or are you currently applying for any patents relating to the content of the manuscript?
- Have you received reimbursements, fees, funding, or salary from an organization that holds or has applied for patents relating to the content of the manuscript?
- Do you have any other financial competing interests?
- Do you have any non-financial competing interests in relation to this paper?

If you can answer no to all of the above, write 'I declare that I have no competing interests' below. If your reply is yes to any, please give details below.

I declare that I have no competing interests

I agree to the open peer review policy of the journal. I understand that my name will be included on my report to the authors and, if the manuscript is accepted for publication, my named report including any attachments I upload will be posted on the website along with the authors' responses. I agree for my report to be made available under an Open Access Creative Commons CC-BY license

(http://creativecommons.org/licenses/by/4.0/). I understand that any comments which I do not wish to be included in my named report can be included as confidential comments to the editors, which will not be published.

Choose an item.

To further support our reviewers, we have joined with Publons, where you can gain additional credit to further highlight your hard work (see: https://publons.com/journal/530/gigascience). On publication of this paper, your review will be automatically added to Publons, you can then choose whether or not to claim your Publons credit. I understand this statement.

Yes Choose an item.
